# A new paradigm for designing ring construction strategies for green organic synthesis: implications for the discovery of multicomponent reactions to build molecules containing a single ring

**DOI:** 10.3762/bjoc.12.236

**Published:** 2016-11-16

**Authors:** John Andraos

**Affiliations:** 1 CareerChem, 504-1129 Don Mills Road, Toronto, ON M3B 2W4 Canada

**Keywords:** atom economy, green organic synthesis, integer partitioning, reactions, probability, retrosynthetic analysis, ring construction strategy

## Abstract

A new way of developing novel synthesis strategies for the construction of monocyclic rings found in organic molecules is presented. The method is based on the visual application of integer partitioning to chemical structures. Two problems are addressed: (1) the determination of the total number of possible ways to construct a given ring by 2-, 3-, and 4-component couplings; and (2) the systematic enumeration of those possibilities. The results of the method are illustrated using cyclohexanone, pyrazole, and the Biginelli adduct as target ring systems with a view to discover new and greener strategies for their construction using multicomponent reactions. The application of the method is also extended to various heterocycles found in many natural products and pharmaceuticals.

## Introduction

The ring motif is a key feature in chemical structures that has long attracted the attention of synthetic organic chemists in their quest to implement novel synthesis strategies. Since ring construction poses significant challenges, it brings forth chemists’ ingenuity and creativity in posing efficient synthetic routes to important target molecules. This is particularly true for complex ring systems found in natural products, such as the celebrated strychnine scaffold, and in pharmaceuticals that typically contain one of several kinds of nitrogen-containing heterocyclic rings. Synthetic organic chemists engaged both in methodology development for the discovery of new transformations and in natural product synthesis to new complex target molecules are now adopting principles of green chemistry. Such principles combine the goals of optimizing reactions to desired products and inventing novel reactions [1–6]. Central to these objectives is the design of highly atom-economical reactions [7–8] that maximize the transfer of atoms found in reactant starting materials to the final desired products. Recently, a measure of the associated probability of achieving reaction intrinsic “greenness” based on simple reaction yield (RY) and atom economy (AE) threshold constraints was advanced [9]. That work demonstrated that both of these metrics, which define “intrinsic greenness”, were critical in influencing whether or not chemical reactions could achieve a minimum standard of overall greenness, regardless of how much auxiliary material (solvents, etc.) was used. Optimization toward overall greenness was best achieved by first maximizing atom economy and reaction yield as far as possible before minimization of auxiliary material consumption. Two points need to be made clear in this discussion. It needs to be emphasized that the design and invention of “intrinsically green” reactions based on high atom economies requires significant chemical ingenuity compared with the simpler task of reducing, replacing, or eliminating solvent usage while maintaining the same chemistry. Furthermore, the bulk of waste from reactions originates from solvents used in work-up extraction and chromatographic purification stages, and not from solvents used in actually carrying out a reaction. Reduction of waste originating from the former group of solvents, however, can present challenges in process chemistry with respect to thermal control, solubility, mixing, and product separation issues when reactions are carried out in very large scale. The idea of “intrinsic greenness” as a core principle based on reaction design was applied to a database of named organic reactions [10] and multicomponent reactions (MCRs) [11–90], written out in a general structural format using Markush structures, to ascertain the fraction of reactions in an organic chemist’s toolbox that meet modest conditions of achieving reaction greenness; namely, reactions having minimum atom economies (AE(min)) above 60%. Once these privileged reactions were selected, probabilities of achieving intrinsic reaction greenness were determined based on satisfying simultaneously the criteria that AE(min) > 60% and RY > 80%. Additionally, since the experimental reaction yield quantity is fractional, the analysis interpreted it as a probability of reaction occurrence to a given product given a set of reaction conditions and starting materials. Reaction outcomes with high yields mean that the probability that they occur is high; conversely those with low yields mean that the probability that they occur is low. Probability versus AE(min) distribution curves were generated for reactions producing various ring containing products according to ring size and types of ring systems. It was found that 5- and 6-membered monocyclic rings are most commonly made by [2 + 2 + 1] and [3 + 2 + 1] cycloadditions where 57% and 76% of them, respectively, have a 100% chance of being intrinsically green from a design perspective. A survey of over 2000 MCRs used to synthesize specific types of heterocyclic rings showed that benzimidazoles, Biginelli adducts, dihydropyridines, furans, pyrans, pyridinones, and thiophenes had a high representation of intrinsic greenness; whereas, a high proportion of MCRs producing chromene-4-ones, coumarins, indoles, and pyrazoles had low probabilities of achieving intrinsic greenness.

In research practice, synthetic organic chemists rely on a combination of retrosynthetic analysis [91–97], similarity and analogy patterning to known reactions, bond dissociation energy and bond polarity analysis (forward and umpolung), chemical intuition, and random occurrences of serendipity to design novel ways to assemble given ring target structures. A favourite example of a serendipitous discovery is when the solvent of a reaction unexpectedly participates as a bone fide reactant rather than behaving as an innocent bystander. Often researchers will tap into their vast library of reactions that they are familiar with from personal experience or through their readings of the literature. Extending known reaction strategy and bond forming-bond breaking themes by analogy is a very useful method. Though retrosynthetic analysis is a powerful tool in the arsenal, its implementation relies entirely on knowledge of known transformations. Similarity and analogy patterning is limited to known reactions as starting points. Serendipity is purely based on accidental occurrences, which are rare, but can be capitalized to advantage by astute researchers. Reliance on all of these strategies to discover new ways to assemble rings is therefore somewhat limiting. There is also the widely held belief in the synthetic organic community that the magnitude of the chemical space of possible transformations that are possible in the forward sense from a finite set of starting building blocks is essentially infinite [98–102] and that despite amassing a database of 1000 or more named reactions and functional group transformations over a period of almost three centuries, synthetic organic chemists have barely scratched the surface in exploring and eventually discovering what transformations are possible in that vast chemical space. In addition, graph theoretical methods have been used to quantify various aspects of synthesis planning and efficiency including codification of construction reactions [103–105], connectivity analysis [106], complexity analysis [107–116], and the creation of encoded synthesis databases that purportedly assist chemists in proposing optimum syntheses to known target molecules subject to constraints, notably number of steps, and cost and availability of commercially available starting materials [117–122]. Despite these advances, these computer-assisted techniques are not routinely adopted by practicing synthetic organic chemists in their everyday work. Instead, they rely on the familiar and tractable methods described earlier.

Given this scenario we sought to address the question of ring construction strategy from a very different perspective that is rooted in an entirely different scientific field which also has enjoyed an even longer track record of research, namely combinatorics and enumeration. Specifically, we exploit the subject of integer partitioning [123–125], which is based on the idea of decomposing a given positive integer into smaller positive integers that add up to it. This topic was first investigated by Leonhard Euler in his book *Introductio in Analysin Infinitorum* published in 1748. This problem is akin to the analogous one considered by ancient Greek mathematicians, namely Eratosthenes, of prime factorization, which is based on decomposing a given positive integer into smaller (prime) numbers in a multiplicative sense. Integers already play prominent roles in chemistry. For example, they appear in molecular formulas of compounds, as stoichiometric coefficients in balanced chemical equations, as oxidation states of elements, as Miller indices and space groups in X-ray crystallography, as quantum numbers in atomic orbitals, as exponents in concentration terms in rate laws, as topological indices in knot theory applied to polymers, and as peak ratios and multiplicities in the characterization of functional groups in NMR peaks. They are also the basis of graph theoretical methods including deducing the expected minimum number of rings and unsaturations for a given molecular formula [126], counting and enumerating all possible structural isomers for a given molecular formula of a hydrocarbon [127–128], and parameterizing chemical properties with topological indices [129–131]. However, none of these integer applications involves partitioning of those integers. In this work we apply the concept of integer partitioning to retrosynthetic analysis of ring structures to systematically decompose given ring frameworks via 2-, 3-, and 4-component couplings akin to decomposing an integer into 2-, 3-, and 4-partitions. In this way we may explore the full spectrum of possible ring fragmentations and assess each possibility with respect to our previously published analysis on determining the likelihood of intrinsic greenness. This work is the first time that integer partitioning has been applied in a chemistry context. In fact, as we will demonstrate later, the ring construction problem posed by a synthetic chemist turns out to be an ideal visual representation of the algebraic, more abstract, problem of integer partitioning. There are two central questions that are considered in integer partitioning. The first is the determination of the total number of 2-, 3-, and 4-partitions of a ring system. In this presentation, we focus exclusively on monocyclic rings. This will lead directly to the total number of possible multicomponent coupling assemblages or fragmentations of a given ring skeleton. The second is the enumeration of those partitions in a systematic manner so that a list of unique combinations for each type of ring partition can be obtained. Simple formulas are used to answer the first question; however, they are unable to enumerate each pathway or possibility pictorially as would be needed to solve the chemical problem of finding assemblages using smaller fragment starting materials to build up a complex ring system, which would be of obvious practical significance to a synthetic chemist. Nevertheless, the tedious task of enumeration can be automated by a simple counting procedure that also eliminates any redundancies. The essential trajectory of tasks presented in this paper is as follows. For a given ring framework, we first obtain the total number of possible 2-, 3-, and 4-partitions. These correspond to all possible 2-, 3-, and 4-component coupling assemblages. The 3- and 4-component couplings are commonly referred to as multicomponent reactions (MCRs). Next, we list and draw out each of these partitions in the form of target bond dissection maps, which highlight the target bonds made in the ring as bolded lines. Then, we permute these maps onto a specific type of ring to list all possible fragmentations of that ring according to a given partition type. This is done simply by overlaying the maps onto the target structure and rotating the fragment framework around the ring, either in a clockwise or anti-clockwise sense. The number and list of permutations of these dissection maps defines the chemical space of possibilities for building up a specified ring framework and is the precise visual representation of the integer partitioning exercise. For example, for a generalized 6-membered ring we find that there are only three possible 3-partitions; namely, [4 + 1 + 1], [3 + 2 + 1], and [2 + 2 + 2]. If we choose a pyridine ring as a target we permute each of these partitions to determine all possible unique [4 + 1 + 1], [3 + 2 + 1], and [2 + 2 + 2] partitions given the symmetry elements of the pyridine ring depending on its substitution pattern. Hence, for 2- or 3-substituted pyridines we have six [4 + 1 + 1], twelve [3 + 2 + 1], and two [2 + 2 + 2] target bond dissection maps; whereas, for 4-substituted pyridines the corresponding numbers are three, six, and one, where the number of each type of fragmentation is reduced by half. In general, rings that contain internal planes of symmetry have significantly fewer possible ring fragmentation patterns for a given partition type. This observation will figure prominently when we extend our present analysis to heterocycles considered in the last section of this paper. Having in hand the list of permutations for a given kind of partition applied to a given kind of ring allows a chemist to sift which ones have been documented in the literature and which ones have not. Of the possibilities that have not been documented a chemist is then forced to ask why this is the case: is it because that assemblage was never considered, or is it a non-viable option due to incompatible mechanistic, kinetic, or thermodynamic considerations. Clearly, if a possibility has not been considered before and is chemically viable, then it would be worth pursuing as a novel synthesis strategy to build up that ring. The integer partitioning method applied to rings therefore is a direct way to identify gaps in synthesis strategies and hence is a valuable aid for the discovery of new reaction assemblages. Since it does not pre-suppose knowledge of existing reactions it is an unbiased procedure. The privileged list of viable possible partitions of a given type on a given ring may be further assessed according to the probability of intrinsic greenness once particular reactant structures are considered as precursors to the desired ring product structure. This allows the attainment of an elite list of “green” options for synthesizing a given target ring structure that satisfies the criteria of “intrinsic” greenness as defined by the inequality conditions imposed on the key metrics atom economy and reaction yield discussed earlier. The final arbiter of whether such options are indeed realizable is, of course, experimental verification.

The structure of the paper is as follows. We first elaborate on the integer partitioning analysis and apply it directly to the construction of monocyclic rings. As a proof of principle exercise, we use the consequences of that analysis to develop novel syntheses of cyclohexanone based on 2-partitions ([5 + 1], [4 + 2], and [3 + 3]) and 3-partitions ([4 + 1 + 1], [3 + 2 + 1], and [2 + 2 + 2]). Next, we apply all possible 3-partitions to the 5-membered pyrazole ring and compare them to what has been done in the literature. Finally, we apply all possible 3-partitions to the 6-membered Biginelli adduct to identify new assemblages for this structure that have potentially high probabilities of intrinsic greenness that exceed the material performance of the traditional way this heterocycle is synthesized from urea, aldehydes, and 1,3-diketones. The method described in this work is quite general and can be applied to any monocyclic structure. The Supporting Information contains an atlas of target bond dissection maps applied to 27 kinds of heterocyclic structures found in natural and pharmaceutical products.

## Results and Discussion

### Multicomponent motifs to build single rings

In this section we consider the partitioning of 3- to 12-membered monocyclic rings according to two-, three-, and four-component couplings since these ring sizes and partitions have immediate applications in synthetic organic chemistry. The formulas for these described partitions can of course be applied to any ring size in a mathematical sense. An interesting observation is that the total number of unique *n*-partitions is given by a polynomial of order *n* – 1. Hence, 2-, 3-, and 4-partitions lead to linear, quadratic, and cubic expressions, respectively. An important point in this analysis is that even- and odd-membered rings are treated separately since no one set of formulas applies to all ring sizes. Tables S1–S4 in Supporting Information File 1 give key ladder patterns and generating sequences of digits that facilitate the determination of the total number of partitions. Also, simple algorithms for enumerating the individual 3- and 4-partitions are given along with worked examples.

#### (i) two-component couplings

Equation 1 gives the relationships for the number of unique two-partitions of monocyclic rings.

**Figure 1:**
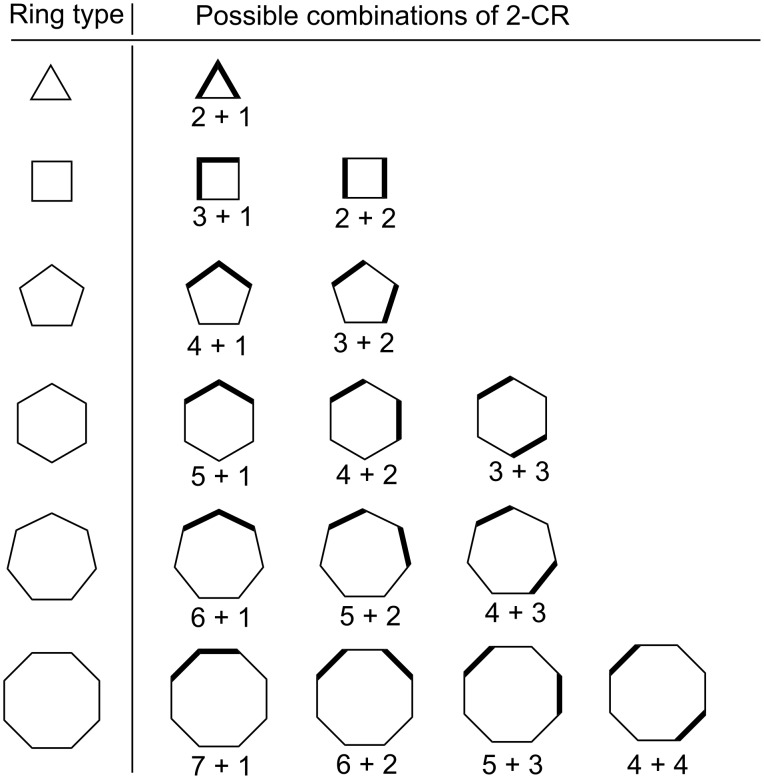
Possible two-component couplings for various monocyclic rings frequently encountered in organic molecules. Synthesis bonds are shown as bolded bonds.

[1]
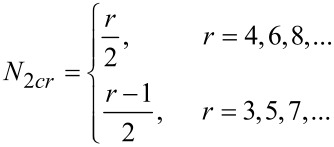


where *r* is the ring size. Table 1 and Table 2 enumerate the possible 2-partitions for even- and odd-membered rings, respectively. Figure 1 shows the corresponding target bond dissection maps for three to eight-membered rings.

**Table 1:** Possible combinations of two-component couplings for various common even-membered monocyclic rings.

Ring size	Possible combinations						Number of combinations

4	3,1	2,2					2
6	5,1	4,2	3,3				3
8	7,1	6,2	5,3	4,4			4
10	9,1	8,2	7,3	6,4	5,5		5
12	11,1	10,2	9,3	8,4	7,5	6,6	6

**Table 2:** Possible combinations of two-component couplings for various common odd-membered monocyclic rings.

Ring size	Possible combinations					Number of combinations

3	2,1					1
5	4,1	3,2				2
7	6,1	5,2	4,3			3
9	8,1	7,2	6,3	5,4		4
11	10,1	9,2	8,3	7,4	6,5	5

#### (ii) three-component couplings

**Figure 2:**
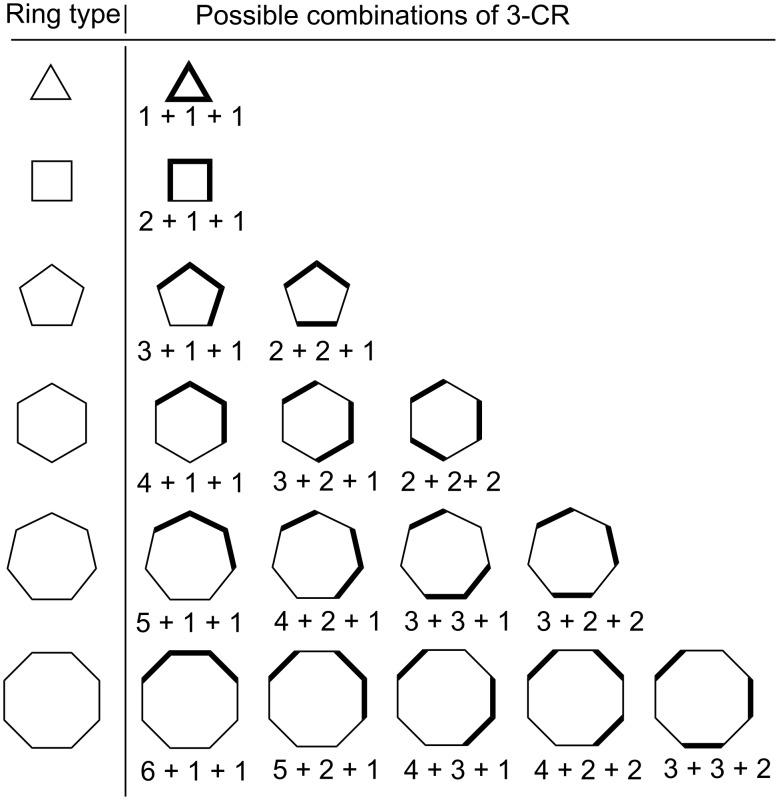
Possible three-component couplings for various monocyclic rings frequently encountered in organic molecules. Synthesis bonds are shown as bolded bonds.

Equation 2 and Equation 3 give the relationships for the number of unique three-partitions of even and odd monocyclic rings, respectively. Table 3 and Table 4 enumerate the possible 3-partitions for even- and odd-membered rings, respectively. Figure 2 shows the corresponding target bond dissection maps for three to eight-membered rings. A key observation about 3-partitions of a ring is that the order of the partition numbers is invariant. For example, a (3,2,1) partition of a six-membered ring framework results in an identical dissection map as a (2,3,1), (2,1,3), (1,2,3), (1,3,2), or (3,1,2) partition. Hence, for any 3-partition arranged in a circle, its algebraic representation is indistinguishable from its ring dissection map representation.

[2]
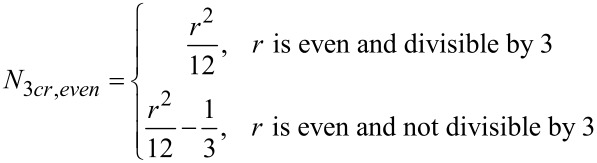


where *r* is the ring size (4, 6, 8, …).

[3]
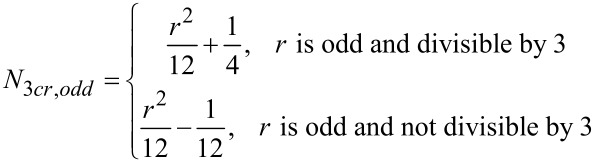


where *r* is the ring size (3, 5, 7, …).

**Table 3:** Possible combinations of three-component couplings for various common even-membered monocyclic rings.

Ring size	Possible combinations					Number of combinations

4	2,1,1					1
6	4,1,1	3,2,1 2,2,2				3
8	6,1,1	5,2,1 4,2,2	4,3,1 3,3,2			5
10	8,1,1	7,2,1 6,2,2	6,3,1 5,3,2 4,3,3	5,4,1 4,4,2		8
12	10,1,1	9,2,1 8,2,2	8,3,1 7,3,2 6,3,3	7,4,1 6,4,2 5,4,3 4,4,4	6,5,1 5,5,2	12

**Table 4:** Possible combinations of three-component couplings for various common odd-membered monocyclic rings.

Ring size	Possible combinations					Number of combinations

3	1,1,1					1
5	3,1,1	2,2,1				2
7	5,1,1	4,2,1 3,2,2	3,3,1			4
9	7,1,1	6,2,1 5,2,2	5,3,1 4,3,2 3,3,3	4,4,1		7
11	9,1,1	8,2,1 7,2,2	7,3,1 6,3,2 5,3,3	6,4,1 5,4,2 4,4,3	5,5,1	10

#### (iii) four-component couplings

**Figure 3:**
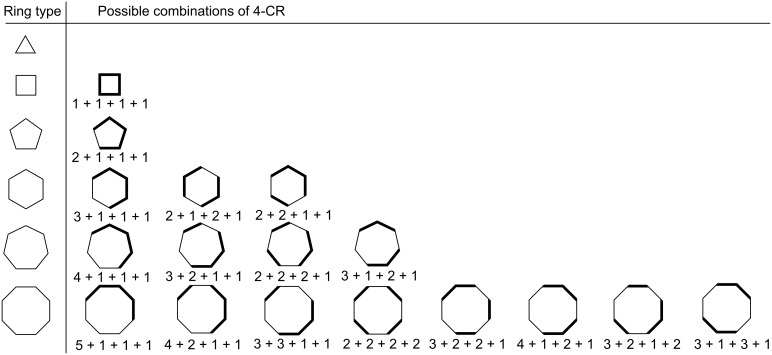
Possible four-component couplings for various monocyclic rings frequently encountered in organic molecules. Synthesis bonds are shown as bolded bonds.

Equation 4 and Equation 5 give the relationships for the number of unique four-partitions of even and odd monocyclic rings, respectively. Table 5 and Table 6 enumerate the possible 4-partitions for even- and odd-membered rings, respectively. Figure 3 shows the corresponding target bond dissection maps for three to eight-membered rings. Unlike 3-partitions, the order of partition elements does matter for any 4-partition. Hence, algebraic representations of 4-partitions are distinguishable from their ring dissection map representations. For example, a (2,2,1,1) partition results in a different dissection map when drawn out in a ring format than a (2,1,2,1) partition though both partitions consist algebraically of the same kinds of fragment elements; namely, two 2s and two 1s. The fourth entry in Figure 3 shows the visual distinction between partitioning a 6-membered ring via [2 + 2 + 1 + 1] and [2 + 1 + 2 + 1] cycloadditions. Similarly, [3 + 2 + 1 + 1] and [3 + 1 + 2 + 1] partitions for a 7-membered ring are distinguishable; and for an 8-membered ring [4 + 2 + 1 + 1] and [4 + 1 + 2 + 1] are distinguishable as are [3 + 2 + 2 + 1] and [3 + 2 + 1 + 2], and [3 + 3 + 1 + 1] and [3 + 1 + 3 + 1].

[4]
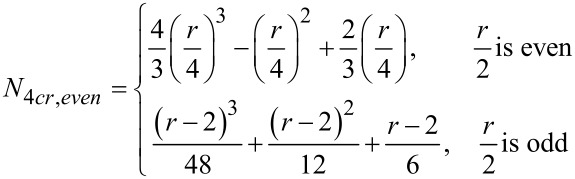


[5]
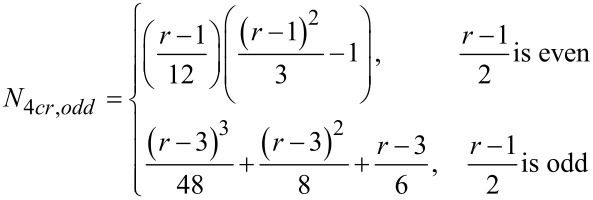


where *r* is the ring size.

**Table 5:** Possible combinations of four-component couplings for various common even-membered monocyclic rings.

Ring size	Possible combinations					Number of combinations

4	1,1,1,1					1
6	3,1,1,1 2,2,1,1	2,1,2,1				3
8	5,1,1,1 4,2,1,1 3,3,1,1	4,1,2,1 3,2,2,1	3,2,1,2	3,1,3,1	2,2,2,2	8
10	7,1,1,1 6,2,1,1 5,3,1,1 4,4,1,1	6,1,2,1 5,2,2,1 4,3,2,1 4,3,1,2 5,2,1,2	4,2,3,1 3,3,3,1	5,1,3,1 4,1,4,1	4,2,2,2 3,3,2,2 3,2,3,2	16
12	9,1,1,1 8,2,1,1 7,3,1,1 6,4,1,1 5,5,1,1	8,1,2,1 7,2,2,1 6,3,2,1 5,4,2,1 7,2,1,2 6,3,1,2	7,1,3,1 6,2,3,1 5,3,3,1 4,4,3,1 5,3,1,3	6,2,2,2 5,3,2,2 4,4,2,2	6,1,4,1 5,2,4,1 4,3,4,1 5,2,3,2 4,3,2,3 5,2,1,4 5,1,5,1 4,2,4,2 4,2,3,3 3,3,3,3	29

**Table 6:** Possible combinations of four-component couplings for various common odd-membered monocyclic rings.

Ring size	Possible combinations					Number of combinations

3						0
5	2,1,1,1					1
7	4,1,1,1 3,2,1,1	3,1,2,1 2,2,2,1				4
9	6,1,1,1 5,2,1,1 4,3,1,1	5,1,2,1 4,2,2,1 3,3,2,1 4,2,1,2	4,1,3,1 3,2,3,1	3,2,2,2		10
11	8,1,1,1 7,2,1,1 6,3,1,1 5,4,1,1	7,1,2,1 6,2,2,1 5,3,2,1 4,4,2,1 6,2,1,2 5,3,1,2	6,1,3,1 5,2,3,1 4,3,3,1 4,3,1,3	5,2,2,2 4,3,2,2	5,1,4,1 4,2,4,1 4,2,3,2 3,3,3,2	20

### Case studies

#### Cyclohexanone

**Figure 4:**
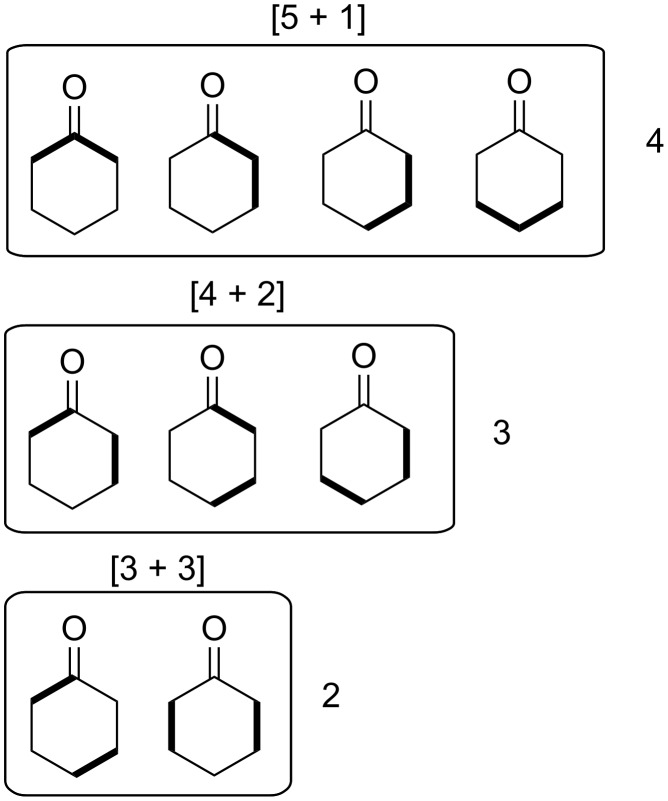
Permutations of two-component coupling patterns for synthesizing the cyclohexanone ring. Synthesis bonds are shown as bolded bonds.

**Scheme 1:**
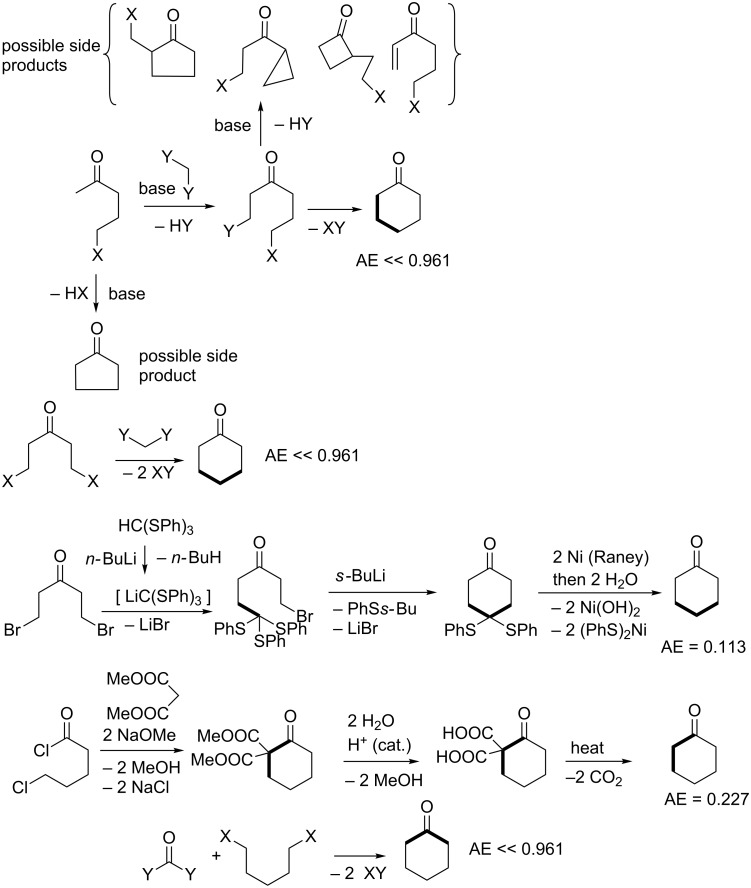
Conjectured syntheses of cyclohexanone via [5 + 1] strategies.

**Scheme 2:**
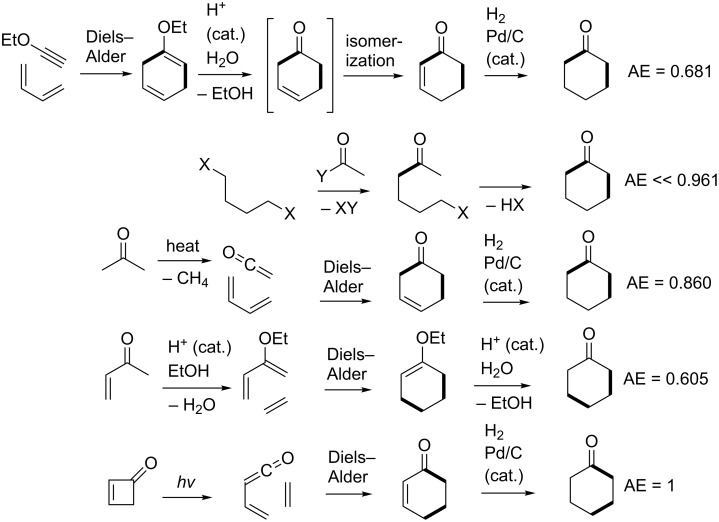
Conjectured syntheses of cyclohexanone via [4 + 2] strategies.

**Scheme 3:**
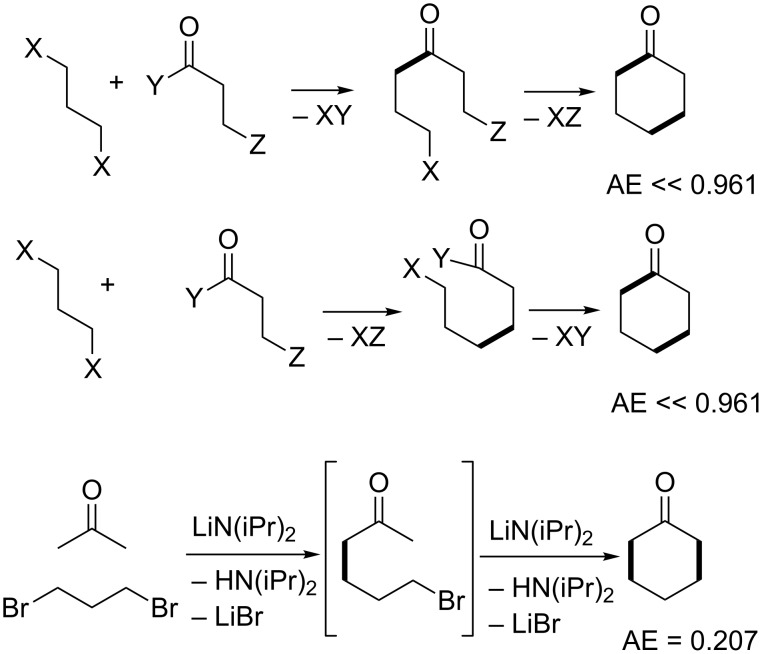
Conjectured syntheses of cyclohexanone via [3 + 3] strategies.

Cyclohexanone is a 6-membered ring containing one asymmetric feature, namely the electrophilic carbonyl group. This molecule is made industrially from precursors that already have the 6-membered ring preformed [132]. Example routes include dehydrogenation of cyclohexanol, which in turn is made either by catalytic hydrogenation of phenol, catalytic hydration of cyclohexene, or catalytic air oxidation of cyclohexane. However, as an intellectual exercise and proof of principle, we may be able to use the results described for integer partitioning to devise creative syntheses for cyclohexanone which involve actual ring construction via 2-component ([5 + 1], [4 + 2], and [3 + 3]) couplings not considered before. Figure 4 shows all permutations of the respective 2-partition target bond dissection maps onto the cyclohexanone ring framework. From these diagrams it is possible to conjecture syntheses according to these partition patterns. These are shown in Schemes 1 to 3. Also included in these schemes are atom economy values for each synthetic sequence. From these suggestions, we find that the [5 + 1] strategies produce the lowest atom economies and give rise to significant side product issues as evidenced by the number of additional unwanted possible side reaction pathways suggested by the analysis. The introduction of a single carbon atom in a ring in a nucleophilic sense may be achieved using dilithiomethane [133–135], tris(phenylthio)methyllithium [136], other reagents using established organolithium chemistry [137–139], or malonate diesters via Claisen condensations followed by hydrolysis and decarboxylation. The greenest routes appear in the [4 + 2] strategy. The three-step route beginning with photochemical ring opening of cyclobutenone to give vinylketene, followed by Diels–Alder addition to ethylene leading to cyclohexenone, followed by hydrogenation is 100% atom economical yielding no byproducts. The next best route with an 86% atom economy is the Diels–Alder addition of ketene, generated by pyrolysis of acetone, to 1,3-butadiene to give cyclohex-3-enone, which upon hydrogenation yields cyclohexanone. The only byproduct of that route is methane, which is produced in the fragmentation of acetone [140].

**Figure 5:**
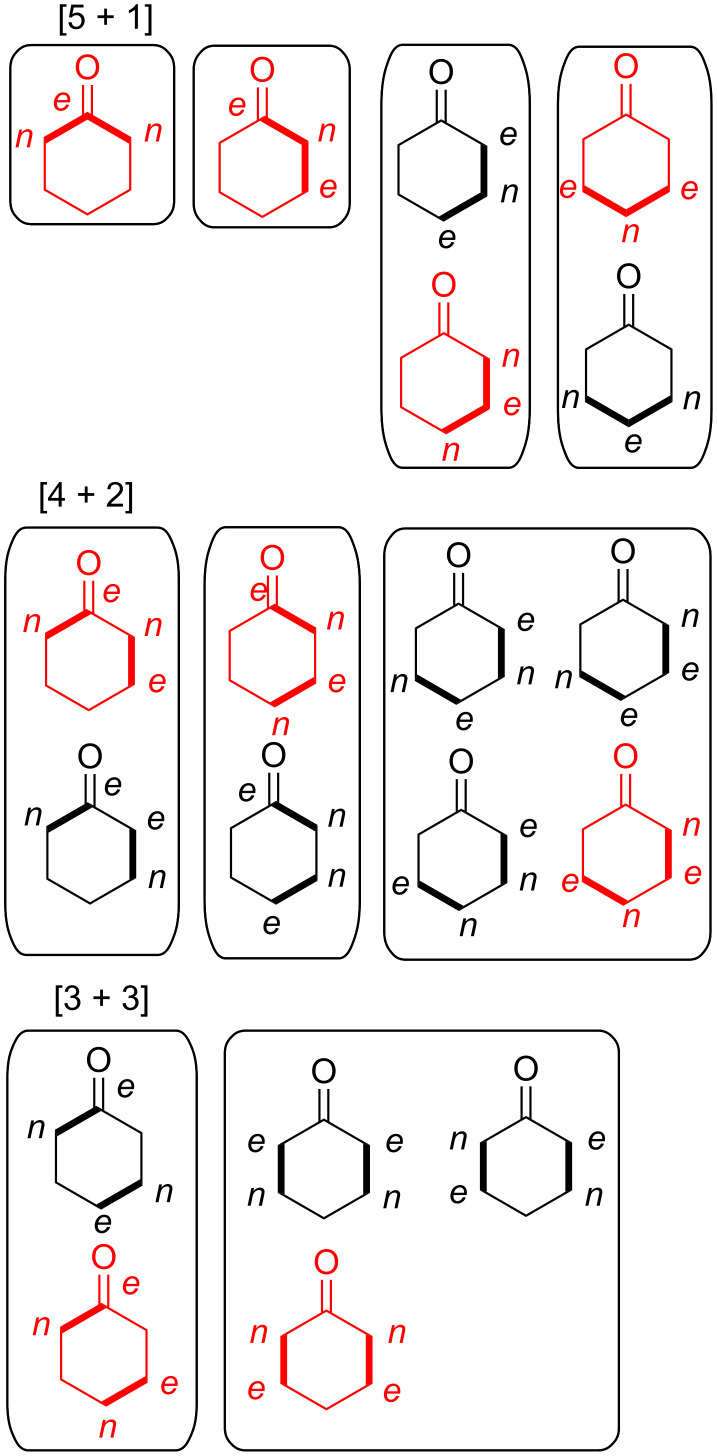
Permutations of two-component coupling patterns for synthesizing the cyclohexanone ring overlayed with nucleophilic (*n*) and electrophilic (*e*) labels at the termini of partition fragments. Synthesis bonds are shown as bolded bonds. Red structures correspond to target templates that form the basis of conjectured syntheses shown in Schemes 1 to 3.

It should be emphasized that the given conjectured routes are a subset of a full spectrum of possible solutions to the problem of making bond connections between nucleophilic and electrophilic centres in the partition fragments. However, since cyclohexanone has only one atom in the ring that is electrophilic, this dictates certain restrictions in the overall possible patterns of how various 2-partition fragments come together to form bonds via ionic connections. Based on [5 + 1], [4 + 2], and [3 + 3] partitions shown in Figure 4, Figure 5 shows the overlay of nucleophilic and electrophilic labels on termini of partition fragments. The combinations considered to create the routes shown in Schemes 1 to 3 are shown in red. Other routes to cyclohexanone may be conjectured based on the remaining combinations. A thorough literature search using Reaxys indicates that cyclohexanone has been made by [5 + 1] and [4 + 2] cycloaddition strategies thus validating the patterns of assembly shown in the third and fourth entries of Scheme 1 and the fourth entry of Scheme 2. The [5 + 1] literature examples involved insertion either of carbon monoxide [141–143], carbon dioxide [144], dichloromethoxymethane [145–146], or methyl methylthiomethyl sulfoxide [147–149] as one-carbon fragments. The only literature example of a [4 + 2] strategy applied to cyclohexanone derivatives involved reaction of but-3-en-2-one with a cyclic enamine followed by reductive elimination of the amine moiety using lithium in liquid ammonia [150].

**Figure 6:**
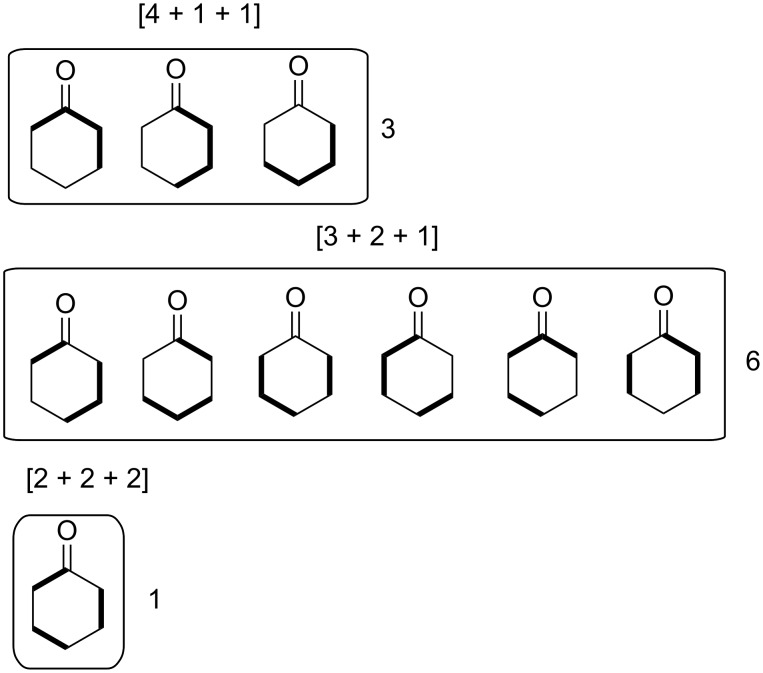
Permutations of three-component coupling patterns for synthesizing the cyclohexanone ring. Synthesis bonds are shown as bolded bonds.

We may be able to repeat the exercise now using all possible 3-partition target bond dissection maps shown in Figure 6. For brevity the results are given in Supporting Information File 1 in Schemes S1 to S3. Among these options the [2 + 2 + 2] strategy of coupling ketene and two equivalents of ethylene is by far the most efficient with an atom economy of 86%. This route matches the closely related second-best performing [4 + 2] route shown in the third entry of Scheme 2. Again, we can superimpose nucleophilic and electrophilic labels on the termini of 3-partition fragments as before to scope out a complete list of possible connection combinations via ionic bond forming processes. These combinations are shown in Supporting Information File 1, Figure S1. Unlike the two-component assemblies, there are no documented literature examples of constructing cyclohexanone by assembly of three fragments. For comparison we also show a similar nucleophilic–electrophilic centre analysis for the synthesis of piperidine by 2- and 3-partitions in Figures S2 and S3, whose structure is also made up of a six-membered ring but contains a pivoting nucleophilic centre instead of an electrophilic one. These results may be contrasted with the analogous five-membered ring compounds cyclopentanone and pyrrolidine in Figures S4 to S7 in Supporting Information File 1. The nucleophilic–electrophilic connectivity patterns for cyclohexanone are the inverse of those for piperidine. The same observation is made when the patterns for cyclopentanone and pyrrolidine are compared. We may conclude that the fewer nucleophilic or electrophilic centres exist in a ring, the more bonding possibilities there are to consider for each partition type. Hence, the construction of hydrocarbon skeletons, containing no pivoting nucleophilic or electrophilic atoms, yields the highest range of possible assemblies and hence the greatest opportunities for creativity and novelty in synthesis design. This explains why such target compounds have attracted the greatest attention among leading synthetic organic chemists [94]. Another feature of key importance that dictates both the partition types and the range of possible assemblies for each partition type is whether the target ring is even- or odd-membered. Even-membered rings often lead to alternating nucleophilic-electrophilic connectivities and hence more direct synthesis routes; whereas, odd-membered rings often lead to connectivities between pairs of nucleophilic termini or pairs of electrophilic termini. This latter situation indicates that an extra redox reaction is a required operation on one of the like centres in order to ligate them. Hence, in order to link two nucleophilic centres, one of them must be oxidized to an electrophilic centre so that it can bond with its nucleophilic partner. Similarly, linking two electrophilic centres requires one of them to be reduced to a nucleophilic centre before bonding can take place. Such additional corrective operations reduce the overall material efficiencies of syntheses of odd-membered rings compared to even-membered rings. This point will be made more evident when we examine literature multicomponent syntheses of pyrazole, a five-membered heterocycle containing two adjacent nitrogen atoms.

#### Pyrazole

**Figure 7:**
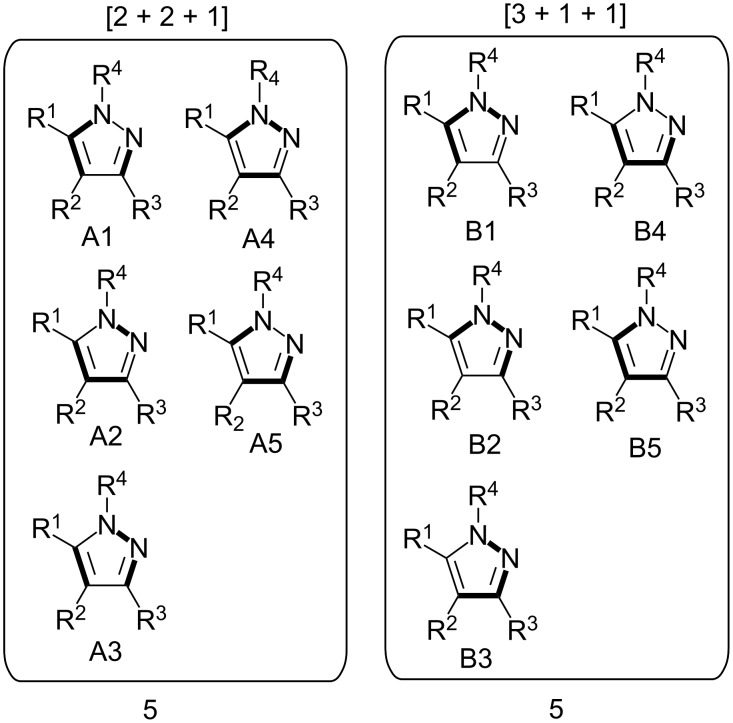
Permutations of three-component coupling patterns for synthesizing the pyrazole ring via [2 + 2 + 1] (A strategies) and [3 + 1 + 1] (B strategies). Synthesis bonds are shown as bolded bonds.

**Scheme 4:**
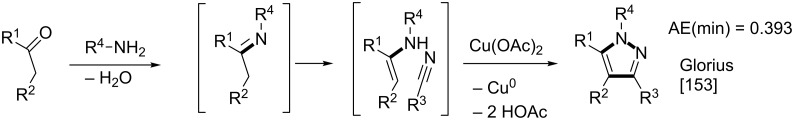
Literature method for constructing the pyrazole ring via the A4 [2 + 2 + 1] strategy.

**Scheme 5:**
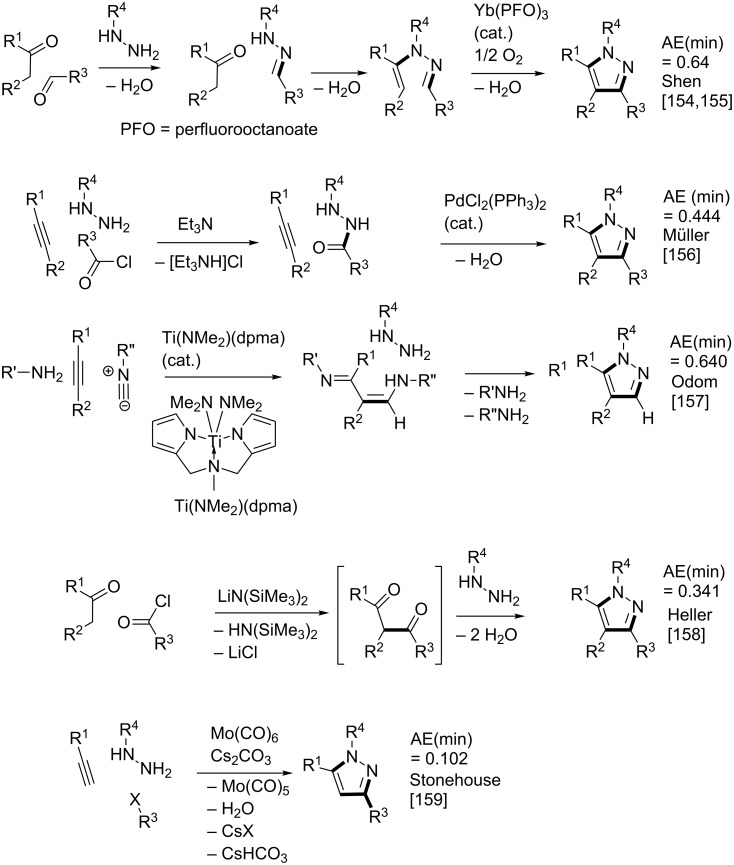
Literature methods for constructing the pyrazole ring via the A5 [2 + 2 + 1] strategy.

**Scheme 6:**
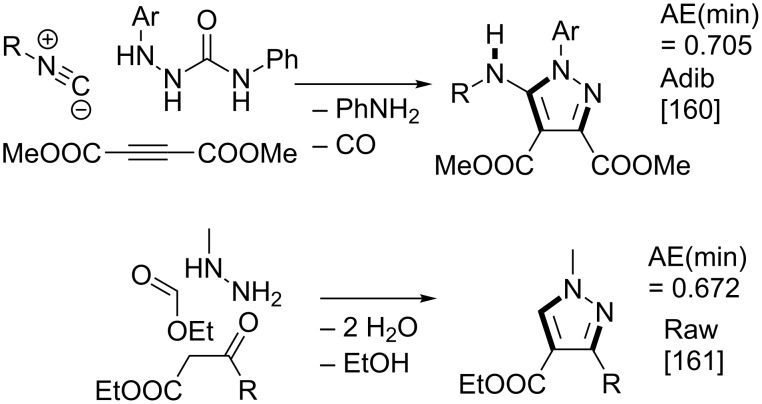
Literature methods for constructing the pyrazole ring via the A1 [2 + 2 + 1] strategy.

**Scheme 7:**
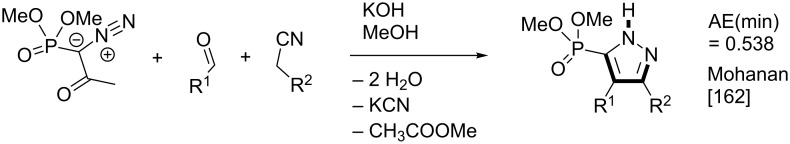
Literature methods for constructing the pyrazole ring via the B4 [3 + 1 + 1] strategy.

**Figure 8:**
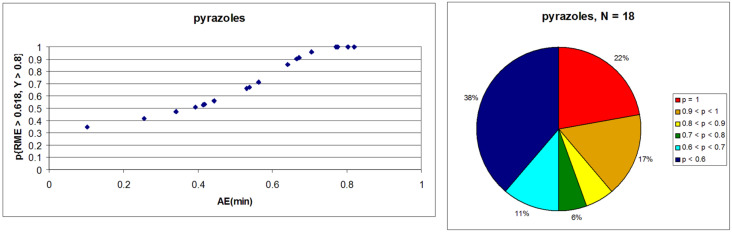
Intrinsic green performance of documented pyrazole syntheses according to [2 + 2 + 1] and [3 + 1 + 1] three-component couplings.

**Scheme 8:**
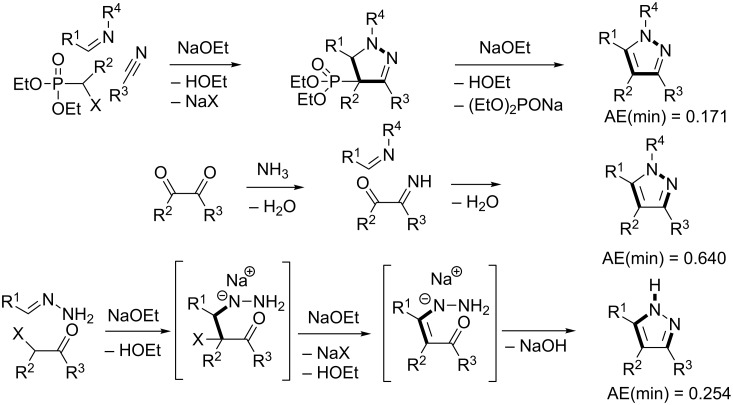
Conjectured reactions for constructing the pyrazole ring via the A2 and A3 [2 + 2 + 1] strategies.

**Scheme 9:**
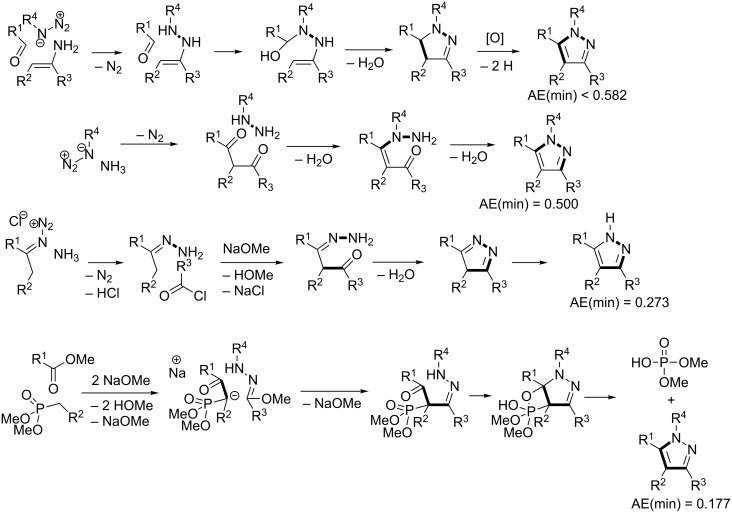
Conjectured reactions for constructing the pyrazole ring via the B1, B2, B3, and B4 [3 + 1 + 1] strategies.

Pyrazole is a well-studied 5-membered heterocycle that has been traditionally synthesized either via the Knorr [151] (1,3-diketone and hydrazine) or von Pechmann [152] (olefin and diazomethane followed by oxidation) strategies. Figure 7 shows the five possible [2 + 2 + 1] (designated as “A” strategies) and five possible [3 + 1 + 1] (designated as “B” strategies) target bond dissection maps for constructing this ring via three-component coupling strategies. Schemes 4 to 6 show literature examples of how this ring was made according to the A4, A5, and A1 [2 + 2 + 1] strategies. Glorius [153] followed the A4 strategy; Shen [154–155], Müller [156], Odom [157], Heller [158], and Stonehouse [159] followed the A5 strategy; and Adib [160] and Raw [161] followed the A1 strategy. Scheme 7 shows a literature example of how this ring was made according to the B4 [3 + 1 + 1] strategy, which was followed by Mohanan [162]. The green performances of these syntheses and others are summarized in Figure 8. About 40% of the documented examples have a better than 90% probability of meeting a moderate level of greenness; however, 40% of them have less than 60% probability of meeting the same criteria. The winning plans are the Adib and Raw strategies since they have high AE(min) thresholds of 71 and 67%, respectively. These performances are closely followed by the Shen and Odom strategies each with AE(min) values of 64%. The Glorius strategy deserves particular comment since it was claimed to have been discovered fortuitously as a result of solvent incorporation into the product structure when the reaction of amines and ketones with α-hydrogens was carried out in acetonitrile. The present partitioning method advanced in this work clearly shows that such a combination of fragments is entirely predictable on the basis of a simple combinatorial analysis that does not violate mechanistic requirements when these fragments come together in an electophilic and nucleophilic sense. The discovery of reaction conditions that experimentally verify the prediction is therefore gratifying and demonstrates that the method is indeed a useful tool in discovering new ways to assemble known rings. Following from the preceding discussion about synthesizing odd-membered rings, the Glorius, Shen, and Stonehouse examples involve redox chemistries in order to complete the ligation to the five-membered pyrazole ring. A literature search revealed that there were no documented examples of the A2, A3, B1, B2, B3, and B5 strategies, which indicates that the chemical space for three-component coupling reactions to pyrazole has not yet been fully explored experimentally. Scheme 8 shows conjectured [2 + 2 + 1] A2 and A3 multicomponent options that appear to be chemically viable. The second entry in Scheme 8 potentially has the highest minimum atom economy among these conjectured couplings. Scheme 9 shows similarly conjectured [3 + 1 + 1] B1, B2, B3, and B5 strategies.

#### Biginelli adduct

**Figure 9:**
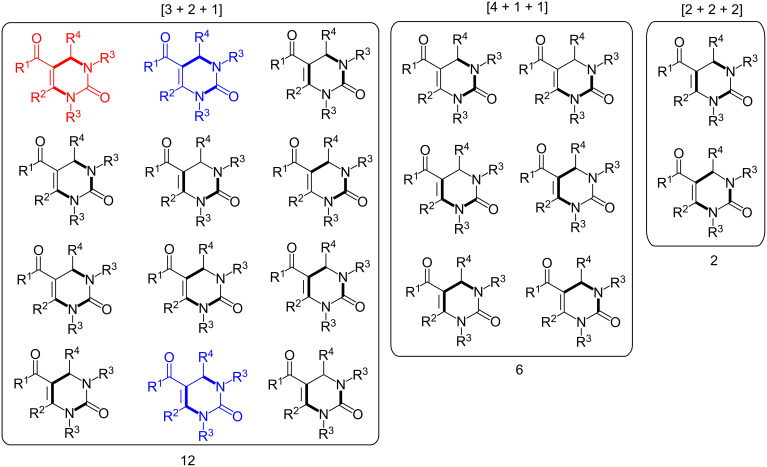
Permutations of three-component coupling patterns for synthesizing the Biginelli ring adduct. Synthesis bonds are shown as bolded bonds.

**Scheme 10:**
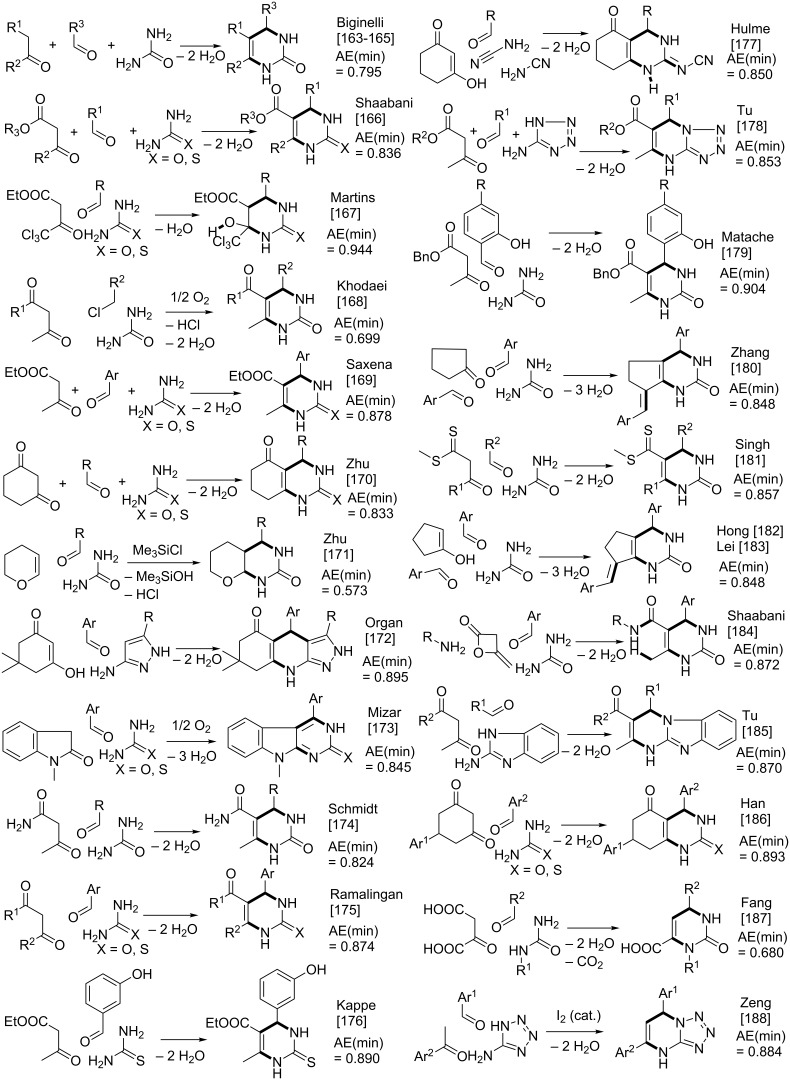
Reported syntheses of the Biginelli adduct via the traditional [3 + 2 + 1] mapping strategy.

**Scheme 11:**
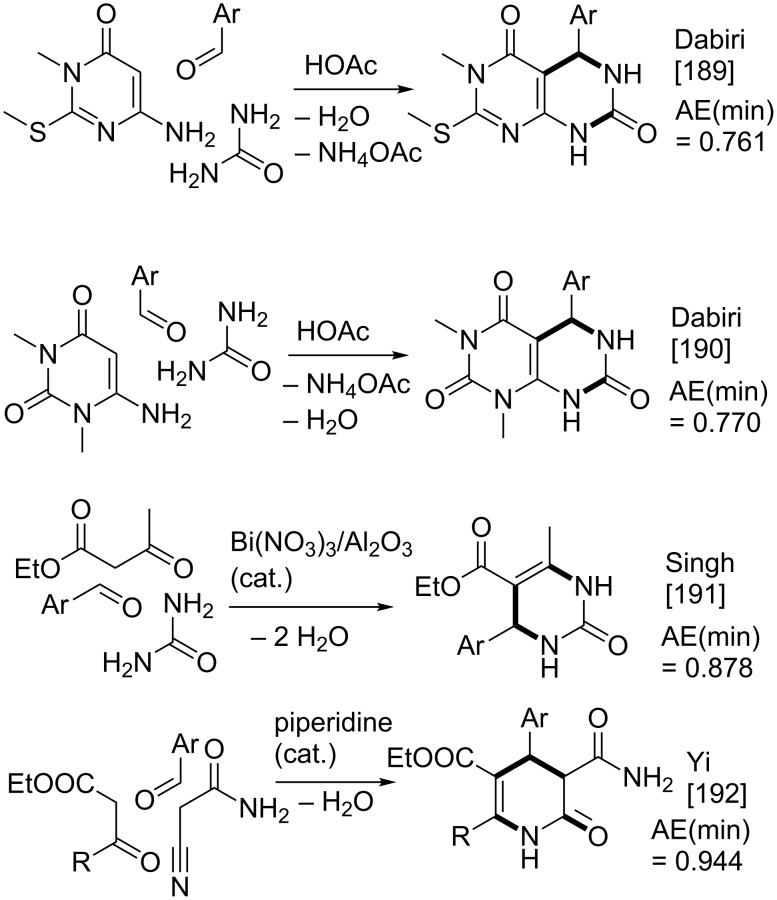
Reported syntheses of the Biginelli adduct via new [3 + 2 + 1] mapping strategies.

**Scheme 12:**
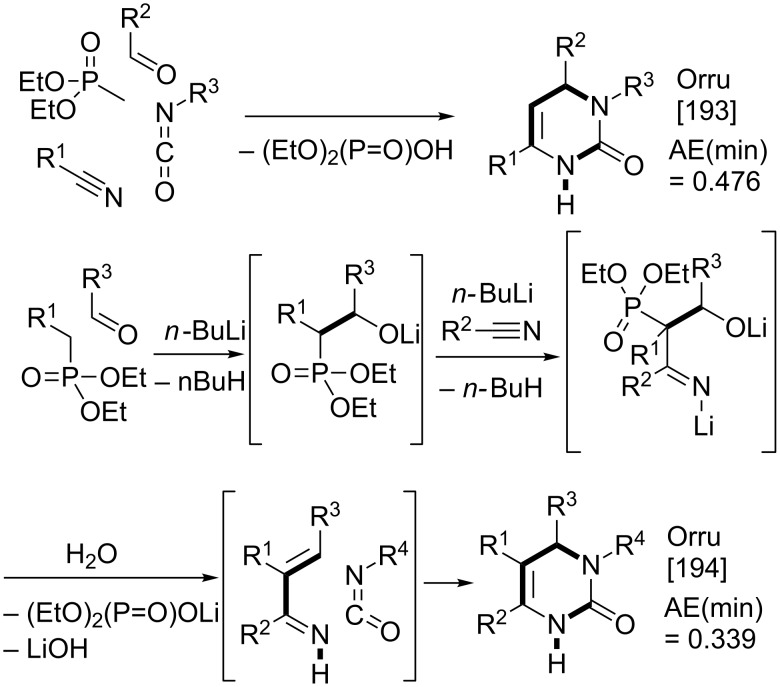
Reported syntheses of the Biginelli adduct via a new [2 + 2 + 1 + 1] mapping strategy.

**Scheme 13:**
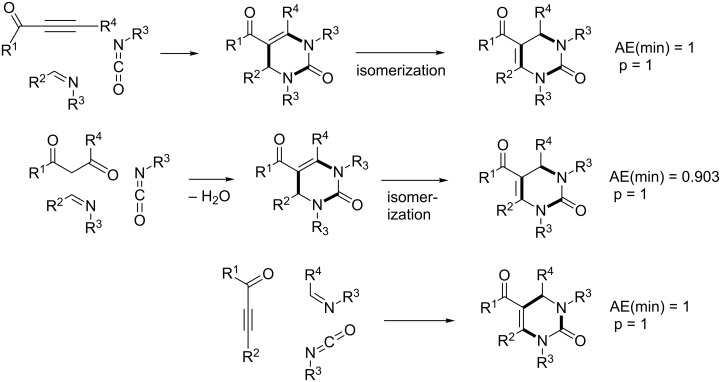
Conjectured syntheses of the Biginelli adduct via new [2 + 2 + 2] mapping strategies.

**Scheme 14:**
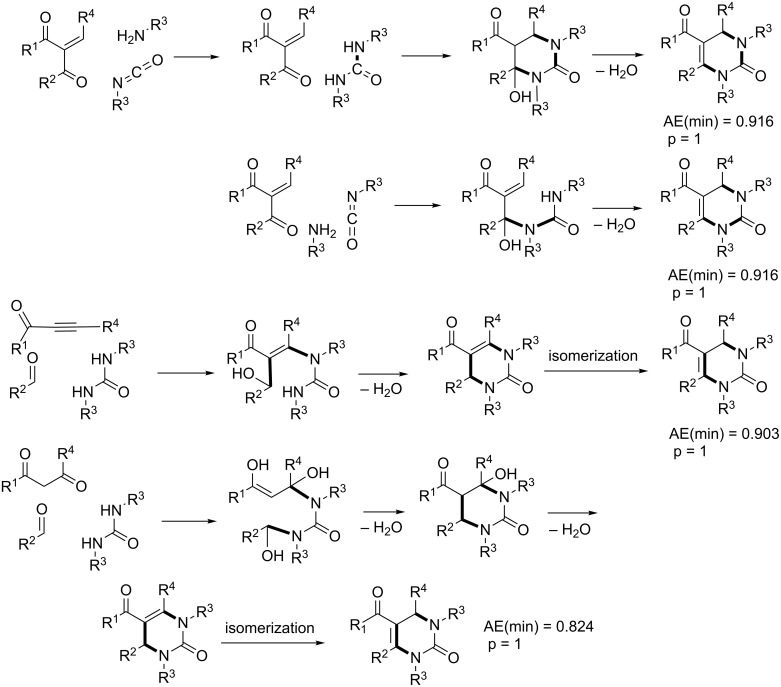
Conjectured syntheses of the Biginelli adduct via new [3 + 2 + 1] mapping strategies.

**Figure 10:**
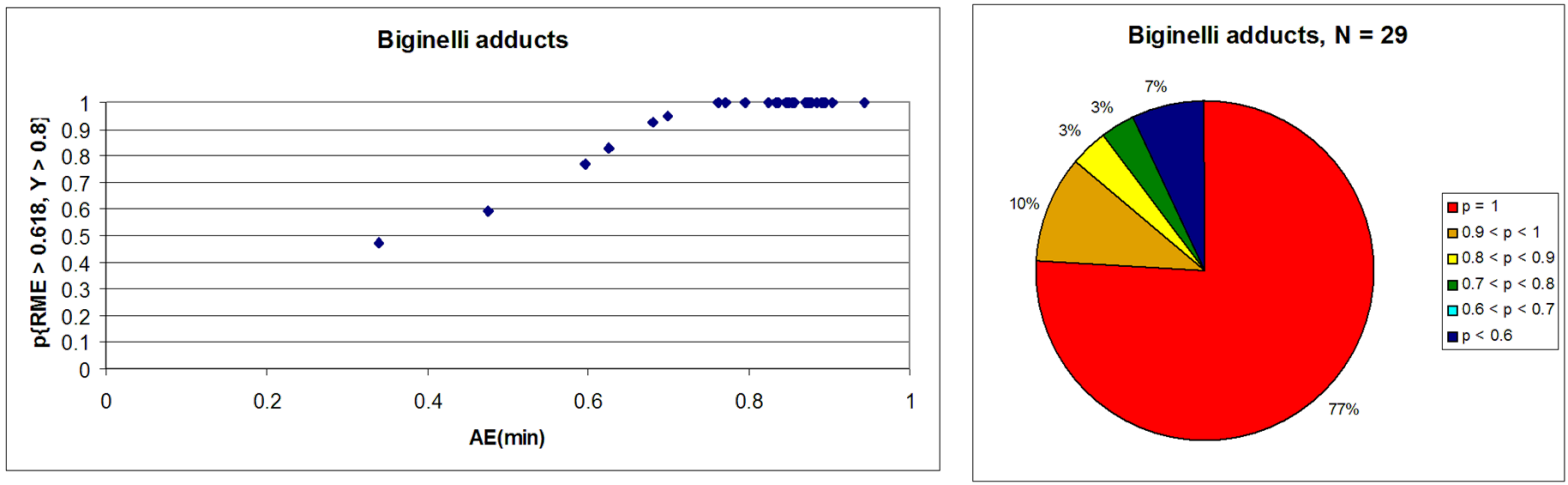
Intrinsic green performance of documented Biginelli adduct syntheses according to [3 + 2 + 1] three-component couplings.

**Figure 11:**
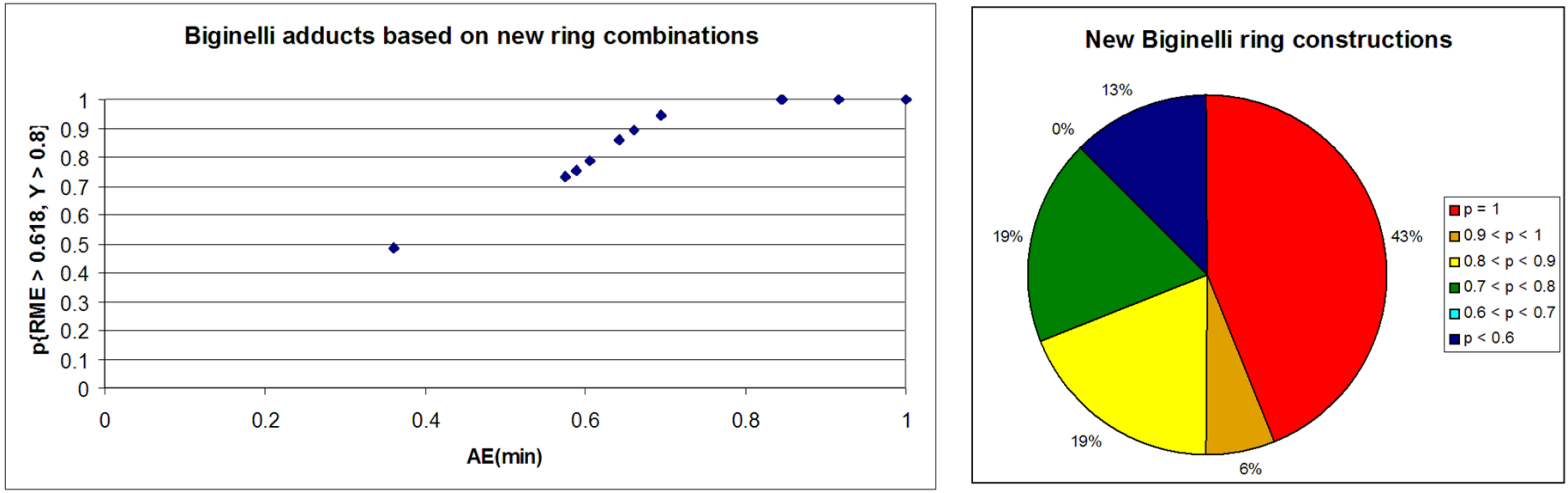
Intrinsic green performance of newly conjectured Biginelli adduct syntheses according to [4 + 1 + 1], [3 + 2 + 1], and [2 + 2 + 2] three-component couplings.

The Biginelli reaction [163–165] is by far the most studied multicomponent reaction since its discovery in 1891 with nearly 2000 citations in the literature. The 3,4-dihydro-1*H*-pyrimidin-2-one adduct has been made essentially by one [3 + 2 + 1] strategy via condensation of 1,3-diketones, urea, and aldehydes. A full integer partitioning and target bond dissection mapping analysis for three-component couplings of this heterocycle, as shown in Figure 9, indicates that the chemical space consists of twelve [3 + 2 + 1], six [4 + 1 + 1], and two [2 + 2 + 2] possible strategies. The traditional mapping is shown in red and only 2 out of 18 novel mappings shown in blue have been reported recently. Scheme 10 shows the following literature examples of [3 + 2 + 1] cycloadditions following the traditional mapping (red structure shown in Figure 9): Biginelli [163–165], Shaabani [166], Martins [167], Khodaei [168], Saxena [169], Zhu [170], Zhu [171], Organ [172], Mizar [173], Schmidt [174], Ramalingan [175], Kappe [176], Hulme [177], Tu [178], Matache [179], Zhang [180], Singh [181], Hong [182], Lei [183], Shaabani [184], Tu [185], Han [186], Fang [187], and Zeng [188]. Scheme 11 shows the following literature examples of [3 + 2 + 1] cycloadditions following novel mappings (blue structures shown in Figure 9): Dabiri [189–190], and Singh [191]. The Yi [192] example follows the traditional coupling using a nitrile instead of a urea precursor, which ultimately leads to a heterocyclic ring that contains only one nitrogen atom instead of two. Scheme 12 shows a novel [2 + 2 + 1 + 1] four-component strategy by Orru [193–194]. Since the Biginelli adduct is an even-membered ring with alternating nucleophilic and electrophilic centres, all but two of the cited examples do not involve redox chemistry and are simply characterized as condensation or coupling reactions. The exceptions, Khodaei and Mizar plans, involve substrates which require a corrective oxidation state change that fortunately do not require additional oxidizing agents beyond oxygen from the air. A full exploration of the remaining target bond dissection maps shown in Figure 9 reveals that there exist potentially new highly atom economical reactions that can lead to the Biginelli adduct. Scheme 13 and Scheme 14 list the most promising candidate reactions employing [2 + 2 + 2] and [3 + 2 + 1] cycloadditions, respectively along with their associated AE(min) estimates and probabilities of intrinsic greenness. In Supporting Information File 1, Schemes S4 and S5 list lesser performing candidates following [3 + 2 + 1] and [4 + 1 + 1] strategies. As was found for the cyclohexanone example, the new [2 + 2 + 2] strategies outperform all others. Figure 10 and Figure 11 show the intrinsic green performances of the literature and newly conjectured syntheses of the Biginelli adduct, respectively. About 90% of literature Biginelli-type syntheses based on one strategy have a better than 90% chance of meeting the intrinsic greenness criterion compared to half of the newly conjectured reactions, based on a much broader range of strategies, found by a thorough partitioning analysis. These findings indicate that there are far more opportunities to pursue novel ways to assemble this product that have not yet been explored.

#### Extension to other monocyclic heterocycles

The methodology presented in this work can in principle be extended to any monocyclic heterocyclic ring system without restriction. In order to motivate synthetic chemists to further explore opportunities to discover new 3-component coupling reactions, an atlas of template target bond 3-partition dissection maps for 27 commonly found heterocyclic rings is given in the Supporting Information File 1 (see Schemes S6 to S32). These include benzimidazole, 2,3-dihydro-1*H*-benzo[*b*][1,4]diazepine, benzofuran, benzopyran, chromen-4-one, coumarin, cyclopent-2-enone, furan, Hantzsch dihydropyridine, hydantoin, imidazole, indole, isoquinoline, isoxazole, oxazole, 4*H*-pyran, pyrazine, pyridazine, pyridine, pyridinone, pyrimidine, pyrimidone, pyrrole, 3*H*-quinazolin-4-one, quinoline, 1*H*-quinolin-4-one, and thiophene. For heterocycles that are composed of a fused aromatic ring, such as benzimidazole, the 3-partitions that do not include the fused junction bond in the set of target synthesis bonds are the ones that have greatest potential for exploration. Essentially choosing starting materials that already contain the aromatic moieties will lead to more efficient and green syntheses. These special structural cases are highlighted in red in the atlas wherever they appear. The Supporting Information File 2 and Supporting Information File 3 also contain an extensive listing in Excel format of literature MCRs for the 27 heterocyclic ring types catalogued along with their AE(min) and intrinsic probability performances. As discussed in the introduction, each of these 3-partition maps needs to be vetted by a thorough literature search to identify those that have not been documented. This set of maps will therefore form the basis of any new avenues of research in synthesis methodology that may be pursued in a meaningful, targeted, and systematic fashion. However, the current structure of literature databases such as SciFinder or Reaxys do not allow for facilitated structure searches based on synthesis strategy maps. What would be needed is for a user to input a target heterocyclic structure highlighting a particular 3- or other partition, rather than just inputting the structure itself. A similarity search would be conducted based on inputted target bond dissection maps already encoded in the database. Essentially each literature citation currently in any search engine, which reports syntheses of ring containing compounds, needs to have their associated target bond maps for products synthesized already included as part of the database in order for the map-to-map similarity search to be implemented. Hence, the present investigation also suggests the creation of a new kind of literature database based on synthesis strategy. The existence of such a powerful tool would have far reaching implications for researchers in synthetic chemistry. Since these scientists are always engaged in inventing novel new assemblages of either new or well-known structures, such a tool can easily sift what strategies have already been documented and allow a chemist to focus his or her efforts on new assemblages of rings not considered before. This will guarantee an answer to the oft-asked question of novelty of a planned synthesis. Furthermore, when coupled with the goals of optimizing syntheses that satisfy atom economical and intrinsic greenness probability thresholds, it can also satisfy the aim of inventing both novel and green syntheses of ring containing compounds.

## Conclusion

The present study advances a new methodology of synthesis planning for ring containing compounds that combines the concept of retrosynthesis with integer partitioning. The determination of the total number of possible 2-, 3-, and 4-partitions of monocyclic rings of any ring size has been worked out. Simple algorithms for their precise enumeration have also been reported for ring sizes commonly encountered in natural products and pharmaceuticals (three to twelve-membered). Target bond dissection maps based on 3-partitions have been applied to syntheses of cyclohexanone, pyrazole, and the Biginelli adduct to identify potentially new three-component coupling reactions for their synthesis. These conjectured reactions were examined for their intrinsic greenness potential based on threshold atom economy and reaction yield values. The application of this methodology was extended to several kinds of monocyclic and fused aromatic heterocyclic rings. We will report on the application of the integer partition algorithm to fused bicyclic and bridged bicyclic ring frameworks elsewhere.

## Supporting Information

Tables S1 to S4 (ladder patterns and generating sequences for determining the total number of unique 3- and 4-partitions of monocyclic rings); simple algorithms for enumerating the sets of unique 3- and 4-partitions of monocyclic rings; Schemes S1 to S3 showing [4 + 1 + 1], [3 + 2 + 1], and [2 + 2 + 2] coupling strategies to synthesize cyclohexanone; Figure S1 to S7 showing nucleophilic-electrophilic centre labels on 3-partition fragment possibilities for cyclohexanone, 2- and 3-partition fragment possibilities for piperidine, 2- and 3- partition fragment possibilities for cyclopentanone, and 2- and 3-partition fragment possibilities for pyrrolidine; Schemes S4 and S5 showing new [3 + 2 + 1] and [4 + 1 + 1] strategies to synthesize the Biginelli adduct; Schemes S6 to S32 showing superposition of 3-partition templates for various heterocycles.

File 1 Application of integer partitioning algorithm to monocyclic rings.

File 2 Excel file of an MCR database of literature routes to various heterocycles.

File 3 Excel file of statistics AE(min) and probability of intrinsic greenness for heterocyclic MCRs.
