# Supplementary material for: A new paradigm for designing ring construction strategies for green organic synthesis: implications for the discovery of multicomponent reactions to build molecules containing a single ring
Source: Beilstein J Org Chem. 2016 Nov 16;12:2420–42. doi: 10.3762/bjoc.12.236 (PMC5238618; doi:10.3762/bjoc.12.236)
Supplement: File 1 — Application of integer partitioning algorithm to monocyclic rings. [file Beilstein_J_Org_Chem-12-2420-s001.pdf]

Supporting Information

for

A new paradigm for designing ring construction strategies for green organic synthesis: implications for the discovery of multicomponent reactions to build molecules containing a single ring

John Andraos

Address: CareerChem, 504-1129 Don Mills Road, Toronto, ON M3B 2W4 Canada

Email: John Andraos - c1000@careerchem.com

*Corresponding author

Application of integer partitioning algorithm to monocyclic rings

Contents

Table S1. Ladder pattern for determining the total number of 3-partitions of even-membered monocyclic rings

Table S2. Ladder pattern for determining the total number of 3-partitions of odd-membered monocyclic rings

Table S3. Ladder pattern for determining the total number of 4-partitions of even-membered monocyclic rings

Table S4. Ladder pattern for determining the total number of 4-partitions of odd-membered monocyclic rings

Enumeration of 3-partitions of monocyclic rings

Enumeration of 4-partitions of monocyclic rings

Schemes S1 to S3 showing three-component coupling strategies to synthesize cyclohexanone.

Figure S1. Nucleophilic-electrophilic labelling of centres in 3-partition fragments of cyclohexanone.

Figure S2. Nucleophilic-electrophilic labelling of centres in 2-partition fragments of piperdine.

Figure S3. Nucleophilic-electrophilic labelling of centres in 3-partition fragments of piperdine.

Figure S4. Nucleophilic-electrophilic labelling of centres in 2-partition fragments of cyclopentanone.

Figure S5. Nucleophilic-electrophilic labelling of centres in 3-partition fragments of cyclopentanone.

Figure S6. Nucleophilic-electrophilic labelling of centres in 2-partition fragments of pyrrolidine.

Figure S7. Nucleophilic-electrophilic labelling of centres in 3-partition fragments of pyrrolidine.

Schemes S4 to S5 showing new three-component coupling strategies to synthesize the Biginelli adduct.

Schemes S6 to S32 showing superposition of 3-partition templates for various heterocycles.

Table S1. Ladder pattern for determining the total number of 3-partitions of even-membered monocyclic rings

Number of 3-partitions	Ring size					
1	**4**	1				
3	**6**	3				
5	**8**	5				
8	**10**	7	1			
12	**12**	9	3			
16	**14**	11	5			
21	**16**	13	7	1		
27	**18**	15	9	3		
33	**20**	17	11	5		
40	**22**	19	13	7	1	
48	**24**	21	15	9	3	
56	**26**	23	17	11	5	
65	**28**	25	19	13	7	1
75	**30**	27	21	15	9	3
85	**32**	29	23	17	11	5

Generating sequence:

$$1, 3, 5, 7, \ldots, (r-3)$$

where r is the ring size (r = 4, 6, 8, 10, …).

Example:
A 16-membered ring has 13 + 7 + 1 = 21 possible three-partitions.

Table S2. Ladder pattern for determining the total number of 3-partitions of odd-membered monocyclic rings

Number of 3-partitions	Ring size					
1	3	1				
2	5	2				
4	7	4				
7	9	6	1			
10	11	8	2			
14	13	10	4			
19	15	12	6	1		
24	17	14	8	2		
30	19	16	10	4		
37	21	18	12	6	1	
44	23	20	14	8	2	
52	25	22	16	10	4	
61	27	24	18	12	6	1
70	29	26	20	14	8	2
80	31	28	22	16	10	4

Generating sequence:

$$2, 4, 6, 8, \ldots, (r-3)$$

where r is the ring size (r = 5, 7, 9, 11, …).

Note that for r = 3 the sequence term is 1.

Example:
A 15-membered ring has 12 + 6 + 1 = 19 possible three-partitions.

Table S3. Ladder pattern for determining the total number of 4-partitions of even-membered monocyclic rings

Number of 4-partitions	Ring size					
1	4	1				
3	6	3				
8	8	7	1			
16	10	13	3			
29	12	21	7	1		
47	14	31	13	3		
72	16	43	21	7	1	
104	18	57	31	13	3	
145	20	73	43	21	7	1
195	22	91	57	31	13	3

Generating sequence:

$$1, 3, 7, 13, 21, \ldots, \left(\frac{r^2}{4} - \frac{3}{2} r + 3 \right),$$

where r is the ring size (r = 4, 6, 8, 10, …).

Example:
A 12-membered ring has 21 + 7 + 1 = 29 possible four-partitions.

Table S4. Ladder pattern for determining the total number of 4-partitions of odd-membered monocyclic rings

Number of 4-partitions	Ring size						
0	3	0					
1	5	1					
4	7	4					
10	9	9	1				
20	11	16	4				
35	13	25	9	1			
56	15	36	16	4			
84	17	49	25	9	1		
120	19	64	36	16	4		
165	21	81	49	25	9	1	
220	23	100	64	36	16	4	
286	25	121	81	49	25	9	1
364	27	144	100	64	36	16	4

Generating sequence:

$$0,1,4,9,16,\ldots,\left(\frac{r^2}{4}-\frac{3}{2}r+\frac{9}{4}\right),$$

where r is the ring size ($r = 3, 5, 7, 9, \ldots$).

Example:
A 15-membered ring has 36 + 16 + 4 = 56 possible four-partitions.

Enumeration of 3-partitions of monocyclic rings:

Step 1: For a given ring size begin with a horizontal list of 2-partitions (*n*, *m*), where *n* is always larger than *m*.

Step 2. Under each (*n*, *m*), write out all 2-partitions of *n* in descending order in a column as follows: (*k*, *l*), *m*, where $k \geq l$.

Step 3: The unique 3-partitions in the array are given by (*k*, *l*, *m*) such that $k \geq l \geq m$.

Example 1:

For a ring size of 12 we have the following array.

2-partitions	11,1	10,2	9,3	8,4	7,5	6,6
3-partitions	(10,1),1					
	(9,2),1	(9,1),2				
	(8,3),1	(8,2),2	(8,1),3			
	(7,4),1	(7,3),2	(7,2),3	(7,1),4		
	(6,5),1	(6,4),2	(6,3),3	(6,2),4	(6,1),5	
		(5,5),2	(5,4),3	(5,3),4	(5,2),5	(5,1),6
				(4,4),4	(4,3),5	(4,2),6
						(3,3),6

The 3-partitions highlighted in yellow are the unique 3-partitions of a 12-membered ring.

Note that partitions of the form $(a,b,c),(b,c,a),(c,a,b),(c,b,a),(a,c,b),$ and (b,a,c) are equivalent due to the inherent cyclic nature of the ring read in clockwise and anti-clockwise senses. For example, (9,2,1) is equivalent to (9,1,2).

Example 2:

For a ring size of 11 we have the following array.

2-partitions	10,1	9,2	8,3	7,4	6,5
3-partitions	(9,1),1				
	(8,2),1	(8,1),2			
	(7,3),1	(7,2),2	(7,1),3		
	(6,4),1	(6,3),2	(6,2),3	(6,1),4	
	(5,5),1	(5,4),2	(5,3),3	(5,2),4	(5,1),5
			(4,4),3	(4,3),4	(4,2),5
					(3,3),5

The 3-partitions highlighted in yellow are the unique 3-partitions of an 11-membered ring.

Even-membered rings will always terminate in a 2-partition equal to $\left(\frac{r}{2},\frac{r}{2}\right)$.

Odd-membered rings will always terminate in a 2-partition equal to $\left(\frac{r+1}{2},\frac{r-1}{2}\right)$.

If r is even and divisible by 3, then

$$\text{total number of elements in array} = \left\{\frac{3}{16}r^2 - \frac{1}{4}r - \frac{1}{4}\right\}$$

$$\text{number of redundancies in array} = \left\{\frac{5}{48}r^2 - \frac{1}{4}r - \frac{1}{4}\right\}$$

$$\text{number of unique 3-partitions} = \frac{1}{12}r^2$$

where {} denotes the nearest integer.

If r is even and not divisible by 3, then

$$\text{total number of elements in array} = \left\{\frac{3}{16}r^2 - \frac{1}{4}r\right\}$$

$$\text{number of redundancies in array} = \left\{\frac{5}{48}r^2 - \frac{1}{4}r + \frac{1}{3}\right\}$$

$$\text{number of unique 3-partitions} = \frac{1}{12}r^2 - \frac{1}{3}$$

where {} denotes the nearest integer.

If r is odd and divisible by 3, then

$$\text{total number of elements in array} = \left\{\frac{3}{16}r^2 - \frac{19}{48}r + \frac{1}{2}\right\}$$

$$\text{number of redundancies in array} = \left\{\frac{5}{48}r^2 - \frac{19}{48}r + \frac{1}{4}\right\}$$

$$\text{number of unique 3-partitions} = \frac{1}{12}r^2 + \frac{1}{4}$$

where {} denotes the nearest integer.

If *r* is odd and not divisible by 3, then

$$\text{total number of elements in array} = \left\{ \frac{9}{48} r^2 - \frac{3}{8} r + \frac{5}{12} \right\}$$

$$\text{number of redundancies in array} = \left\{ \frac{5}{48} r^2 - \frac{3}{8} r + \frac{1}{3} \right\}$$

$$\text{number of unique 3-partitions} = \frac{1}{12} r^2 - \frac{1}{12}$$

where {} denotes the nearest integer.

Enumeration of 4-partitions of monocyclic rings:

Step 1: For a given ring size begin with a horizontal list of 3-partitions (*n*, *m, l*), where $n \geq m \geq l$ as determined by the method of enumeration of 3-partitions described above.

Step 2. Under each (*n*, *m*, *l*), write out all 2-partitions of *n* in descending order in a column as follows: (*u*, *v*), *m*, *l* where $u \geq v$.

Step 3: Repeat step 2 for the *m* values.

Step 4: Select unique 4-partitions from array. For 4-partitions containing two identical digits, such as (*x*, *x*, *y*, *z*), ensure that the form (*x*, *y*, *x*, *z*) is also present in the unique set.

Example 1:

For a ring size of 12 we have the following array.

3-partitions	10,1,1	9,2,1	8,3,1	7,4,1	6,5,1	8,2,2	7,3,2	6,4,2	5,5,2	6,3,3	5,4,3	4,4,4
4-partitions	(9,1),1,1											
	(8,2),1,1	(8,1),2,1										
	(7,3),1,1	(7,2),2,1	(7,1),3,1			(7,1),2,2						
	(6,4),1,1	(6,3),2,1	(6,2),3,1	(6,1),4,1		(6,2),2,2	(6,1),3,2					
	(5,5),1,1	(5,4),2,1	(5,3),3,1	(5,2),4,1	(5,1),5,1	(5,3),2,2	(5,2),3,2	(5,1),4,2		(5,1),3,3		
			(4,4),3,1	(4,3),4,1	(4,2),5,1	(4,4),2,2	(4,3),3,2	(4,2),4,2	(4,1),5,2	(4,2),3,3	(4,1),4,3	
					(3,3),5,1			(3,3),4,2	(3,2),5,2	(3,3),3,3	(3,2),4,3	(3,1),4,4
												(2,2),4,4
							(4,3),2,3					
		9,(1,1),1	8,(2,1),1	7,(3,1),1	6,(4,1),1	8,(1,1),2	7,(2,1),2	6,(3,1),2	5,(4,1),2	6,(2,1),3	5,(3,1),3	4,(3,1),4
				7,(2,2),1	6,(3,2),1			6,(2,2),2	5,(3,2),2		5,(2,2),3	4,(2,2),4

The 4-partitions highlighted in yellow are the unique 4-partitions of a 12-membered ring.

Example 2:

For a ring size of 11 we have the following array.

3-partitions	9,1,1	8,2,1	7,3,1	6,4,1	5,5,1	7,2,2	6,3,2	5,4,2	5,3,3	4,4,3
4-partitions	**(8,1),1,1**									
	**(7,2),1,1**	**(7,1),2,1**								
	**(6,3),1,1**	**(6,2),2,1**	**(6,1),3,1**			(6,1),2,2				
	**(5,4),1,1**	**(5,3),2,1**	**(5,2),3,1**	**(5,1),4,1**		**(5,2),2,2**	(5,1),3,2			
		**(4,4),2,1**	**(4,3),3,1**	**(4,2),4,1**	(4,1),5,1	**(4,3),2,2**	**(4,2),3,2**	(4,1),4,2	(4,1),3,3	
				(3,3),4,1	(3,2),5,1		**(3,3),3,2**	(3,2),4,2	(3,2),3,3	(3,1),4,3
										(2,2),4,3
		8,(1,1),1	7,(2,1),1	6,(3,1),1	5,(4,1),1	7,(1,1),2	**6,(2,1),2**	**5,(3,1),2**	5,(2,1),3	**4,(3,1),3**
				6,(2,2),1	5,(3,2),1			5,(2,2),2		4,(2,2),3

The 4-partitions highlighted in yellow are the unique 4-partitions of an 11-membered ring.

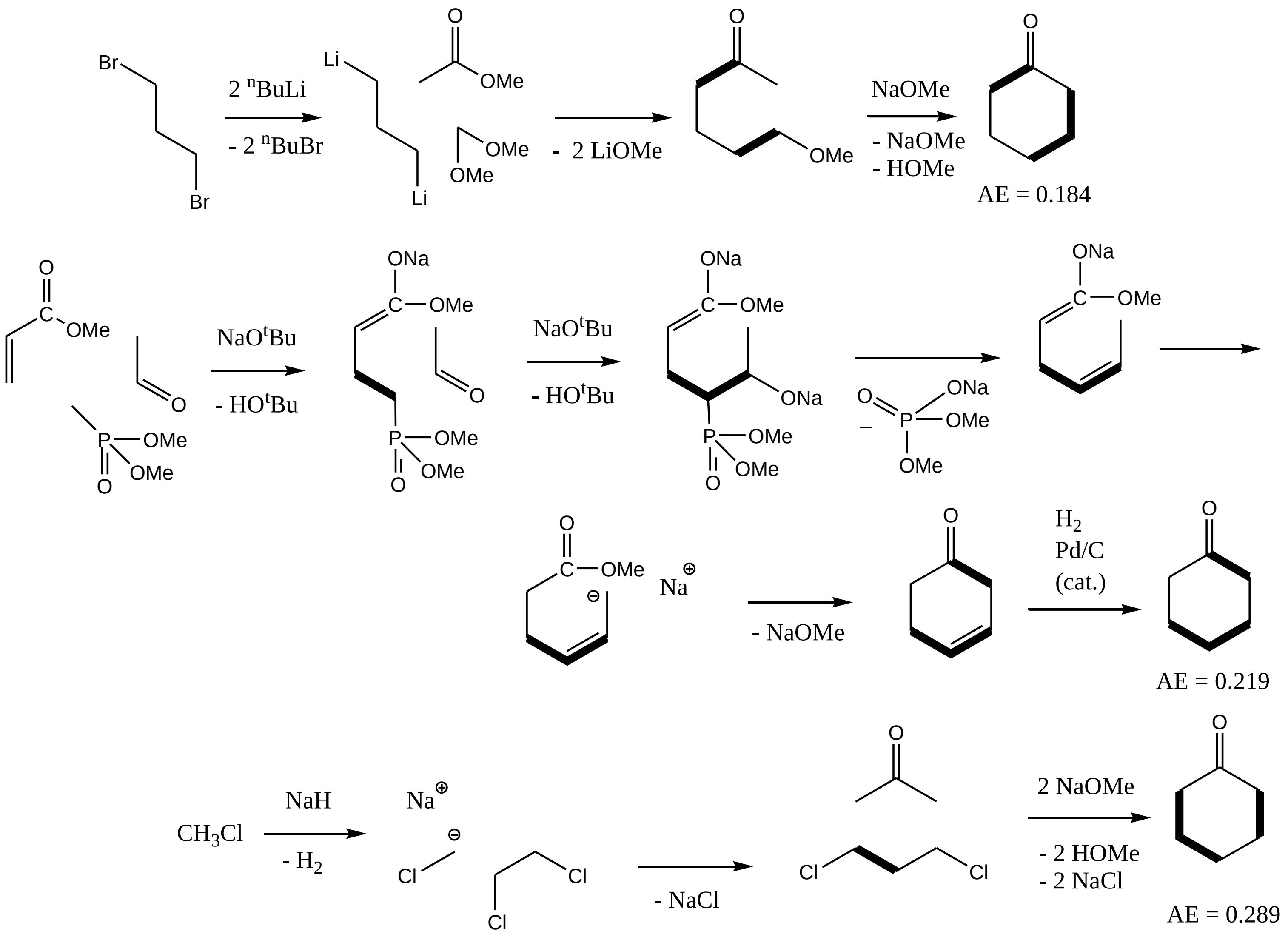

Scheme S1a. Three-component coupling sequences to make cyclohexanone via the [3 + 2 + 1] strategy.

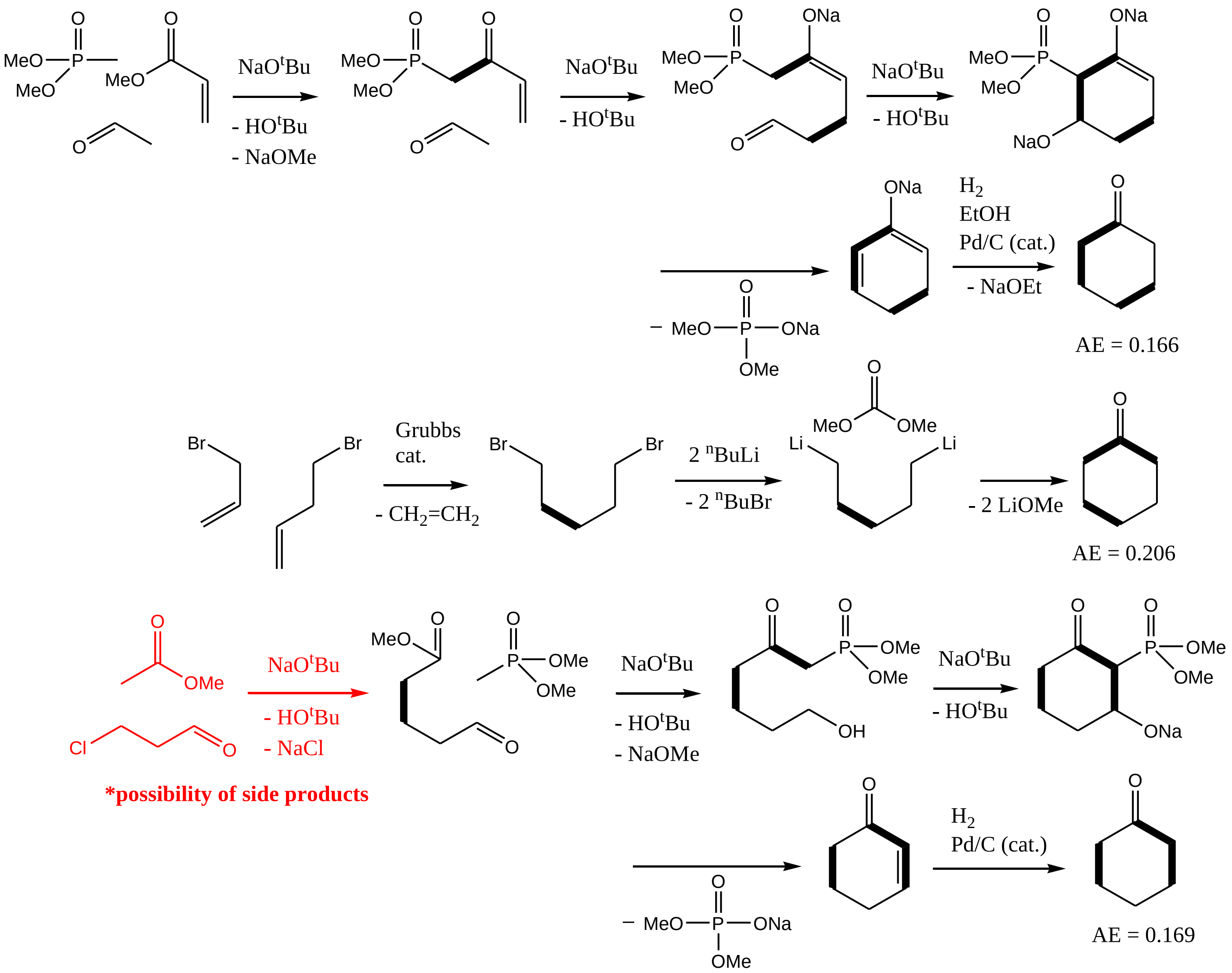

Scheme S1b. Three-component coupling sequences to make cyclohexanone via the [3 + 2 + 1] strategy. (continued)

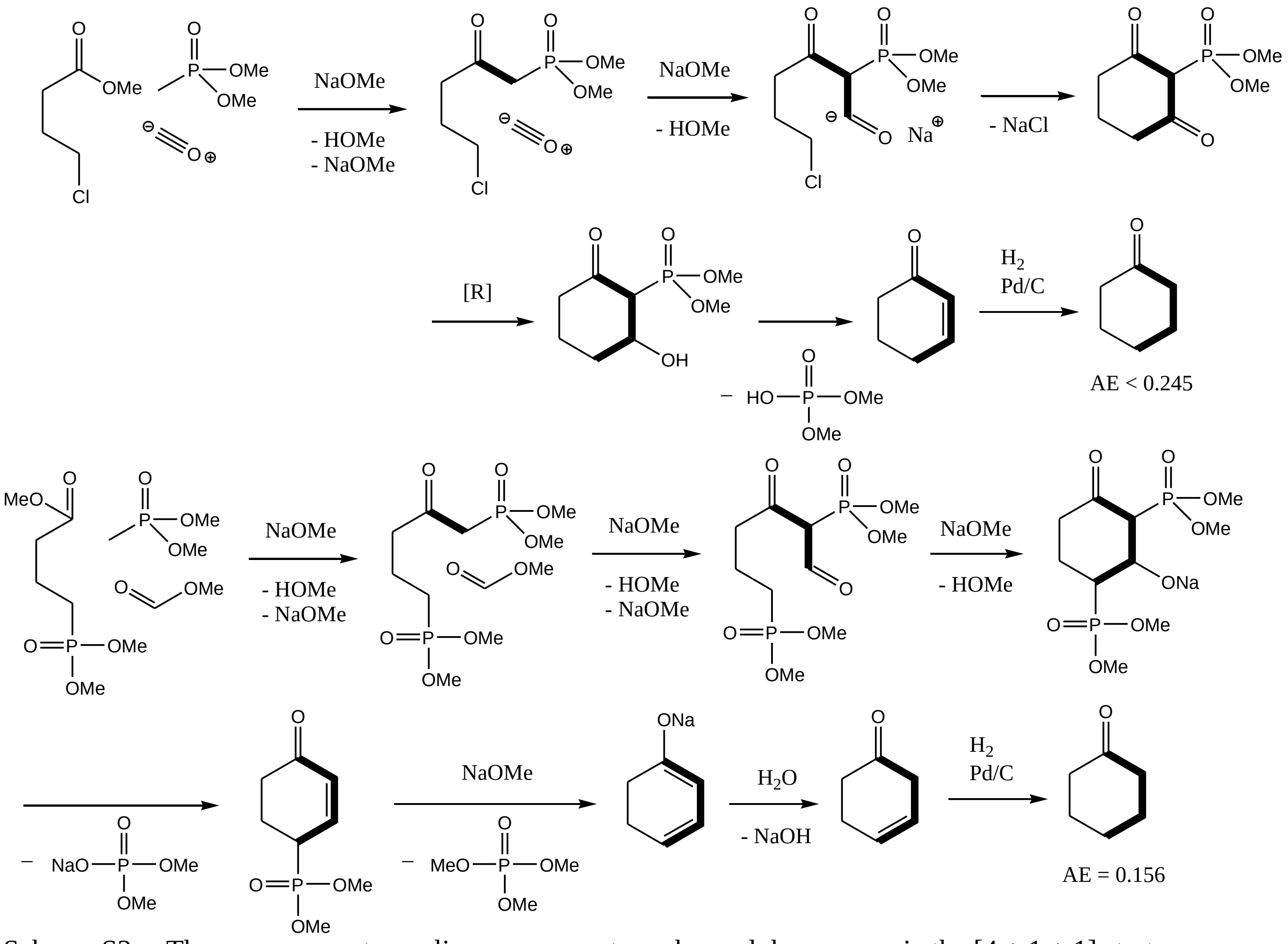

Scheme S2a. Three-component coupling sequences to make cyclohexanone via the [4 + 1 + 1] strategy.

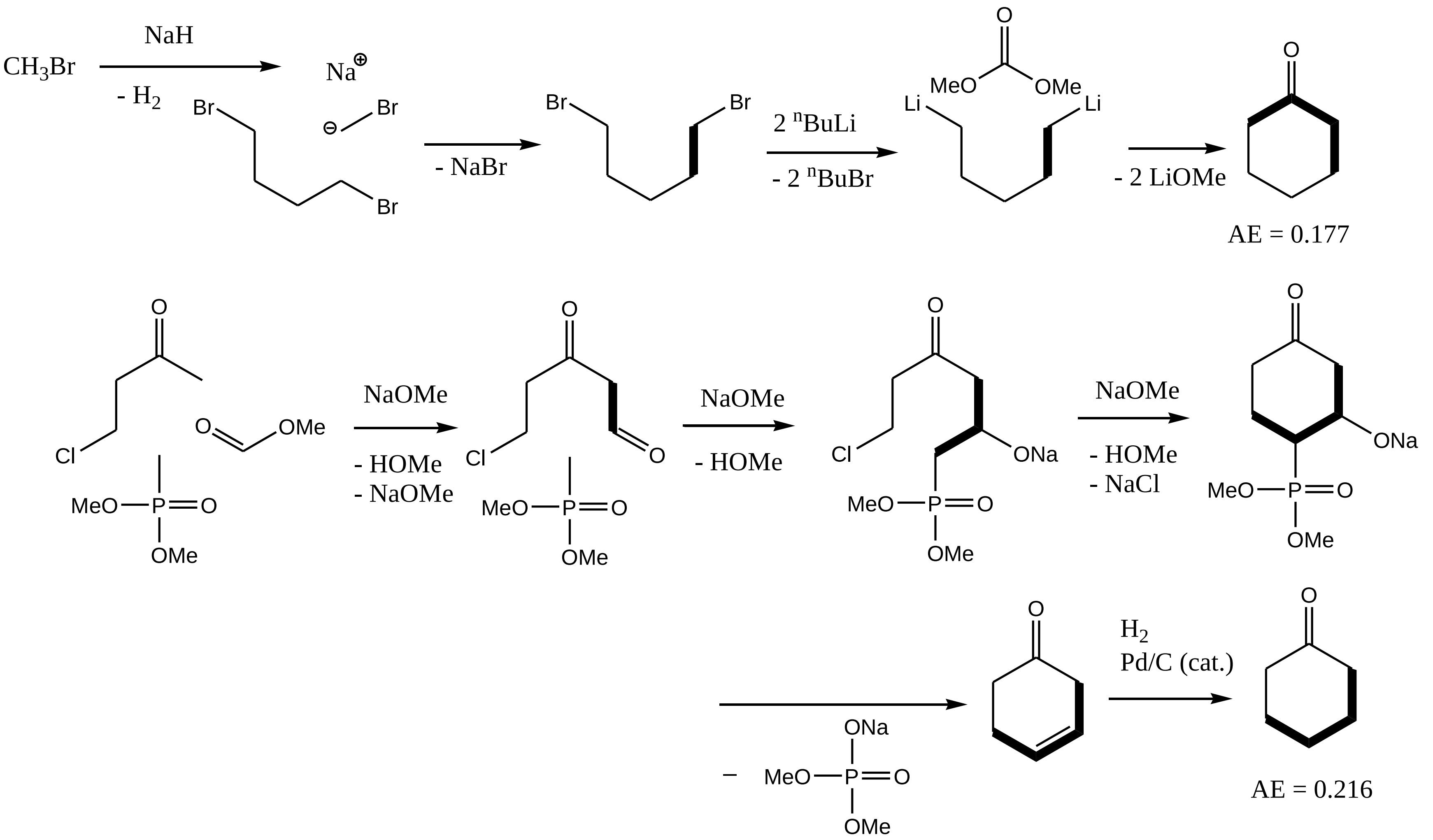

Scheme S2b. Three-component coupling sequences to make cyclohexanone via the [4 + 1 + 1] strategy. (continued)

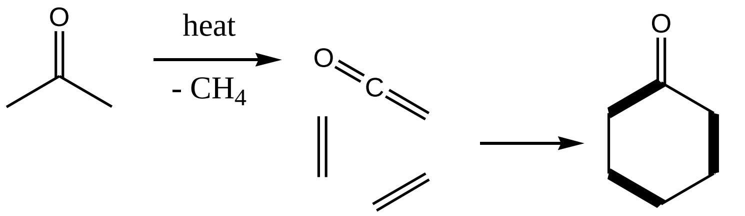

AE = 0.860

Scheme S3. Three-component coupling sequence to make cyclohexanone via the [2 + 2 + 2] strategy.

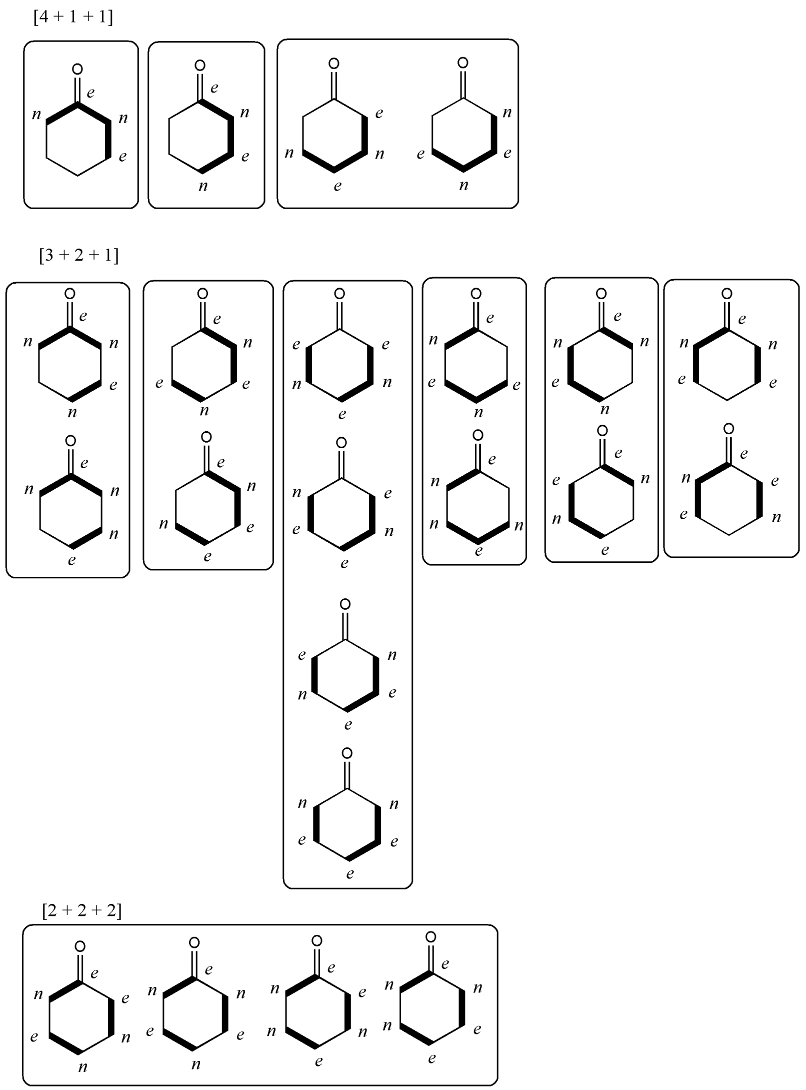

Figure S1. Nucleophilic-electrophilic labelling of centres in 3-partition fragments of cyclohexanone.

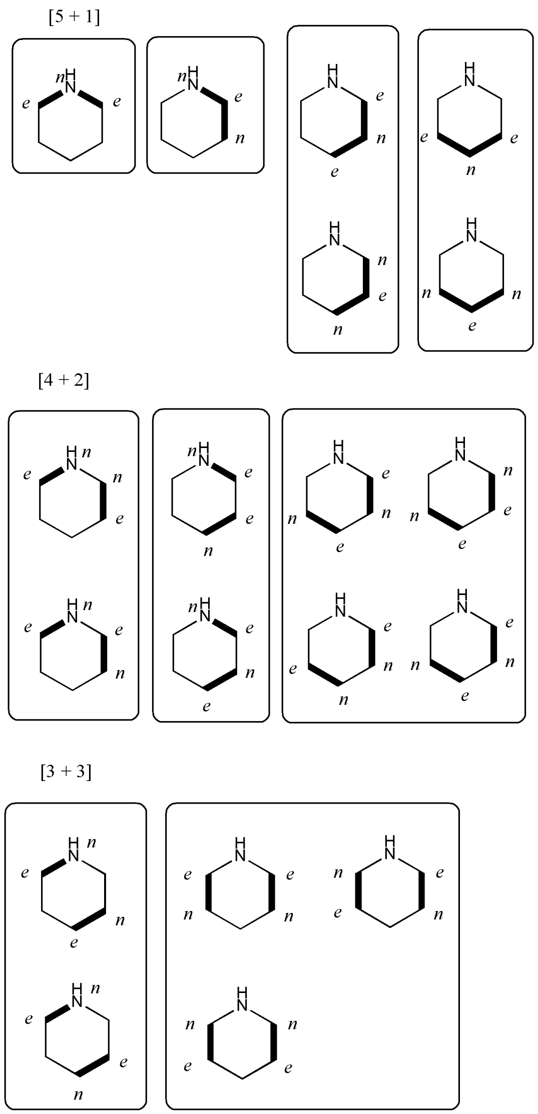

Figure S2. Nucleophilic-electrophilic labelling of centres in 2-partition fragments of piperidine.

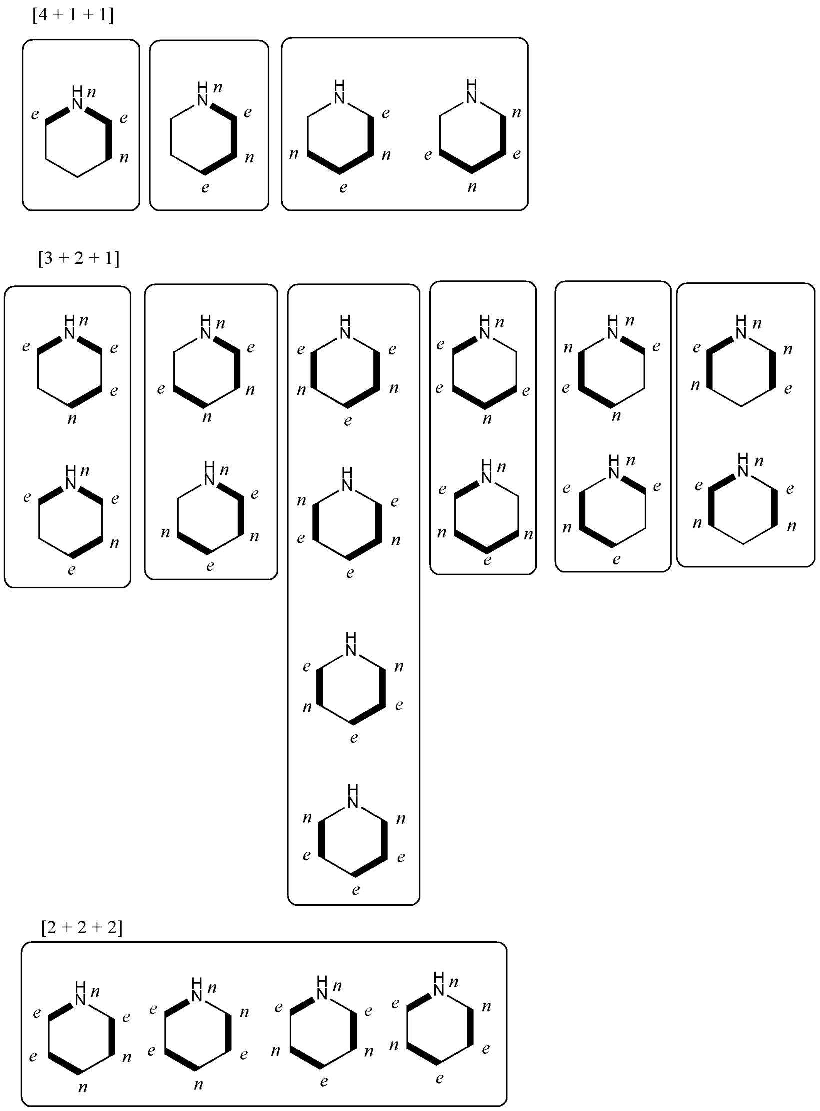

Figure S3. Nucleophilic-electrophilic labelling of centres in 3-partition fragments of piperidine.

[4 + 1]

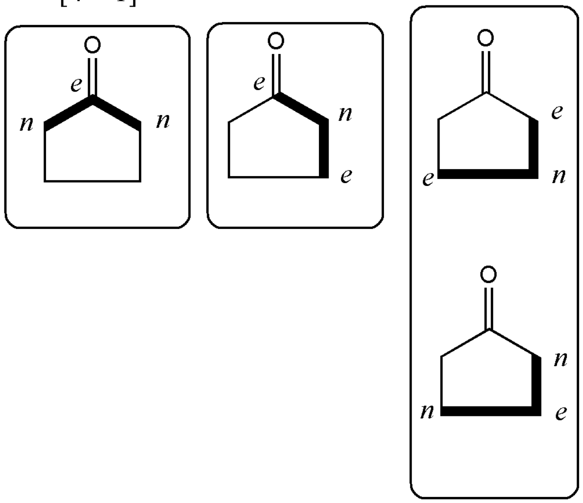

[3 + 2]

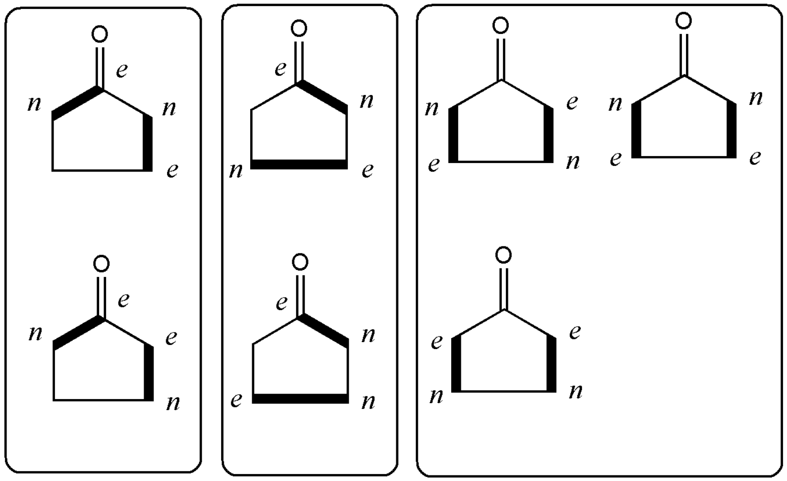

Figure S4. Nucleophilic-electrophilic labelling of centres in 2-partition fragments of cyclopentanone.

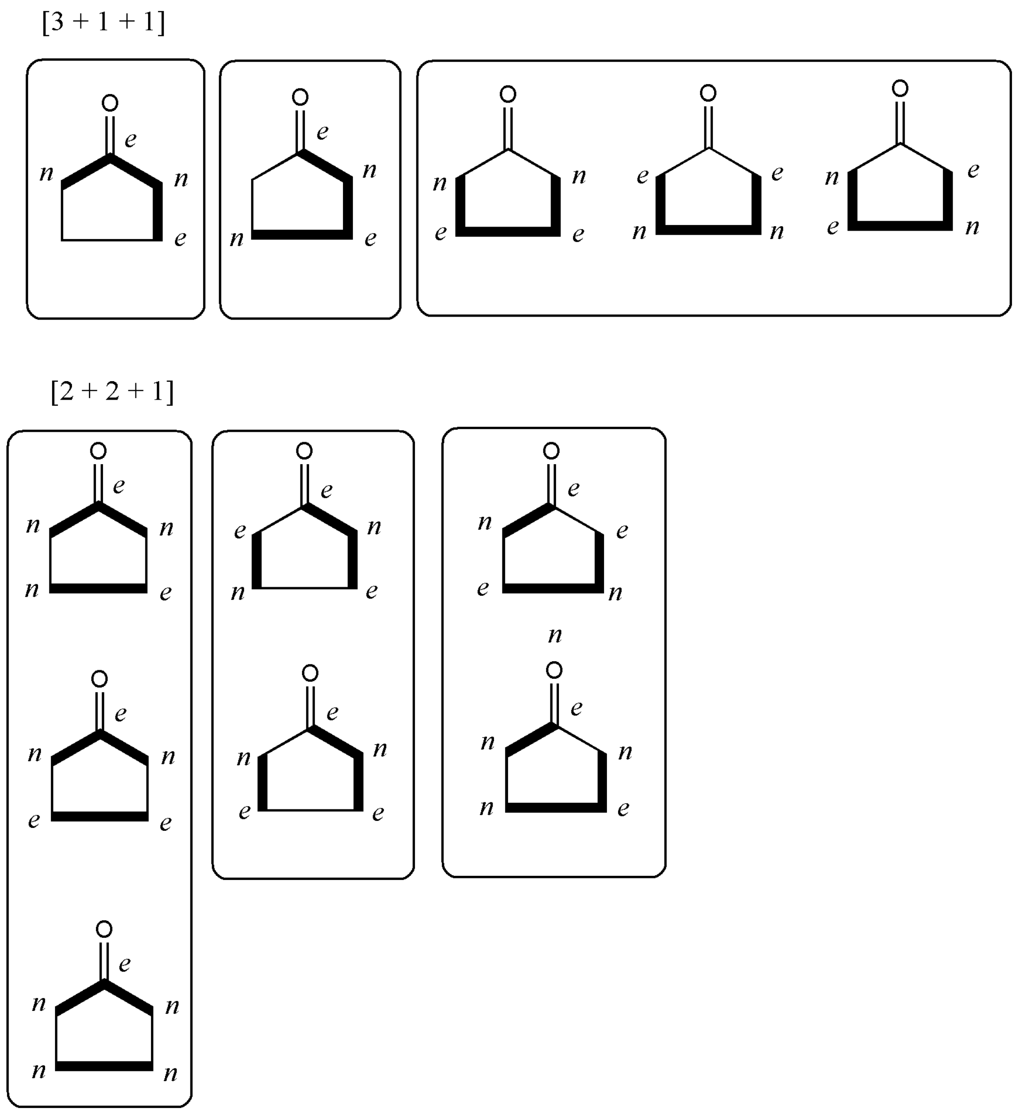

Figure S5. Nucleophilic-electrophilic labelling of centres in 3-partition fragments of cyclopentanone.

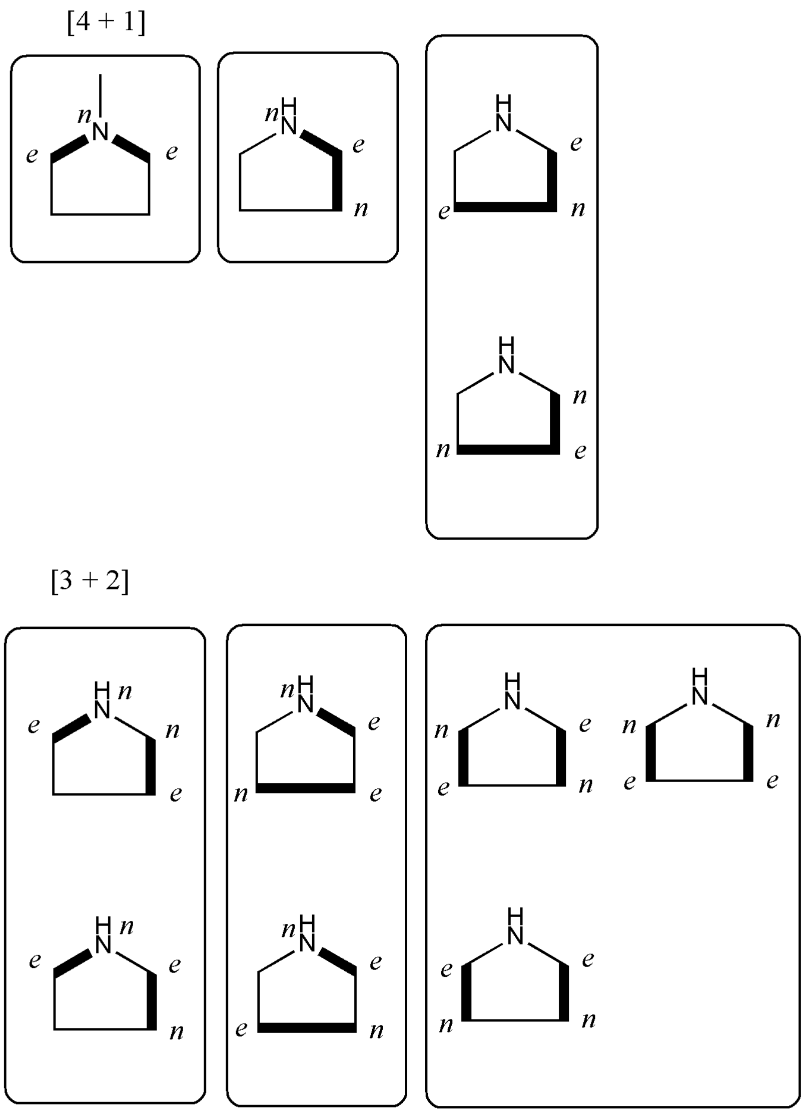

Figure S6. Nucleophilic-electrophilic labelling of centres in 2-partition fragments of pyrrolidine.

[3 + 1 + 1]

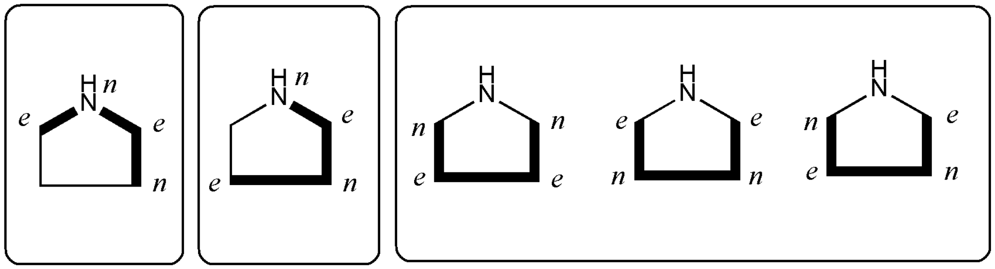

[2 + 2 + 1]

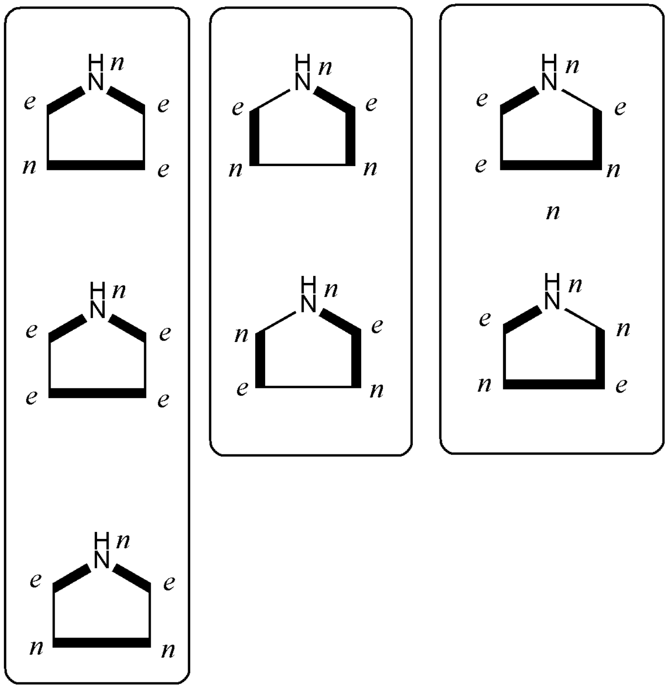

Figure S7. Nucleophilic-electrophilic labelling of centres in 3-partition fragments of pyrrolidine.

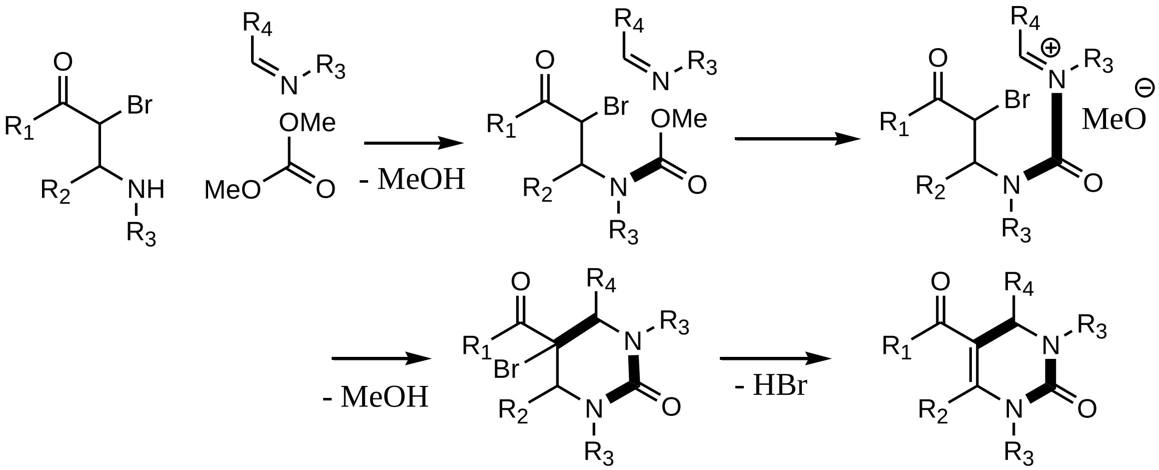

AE(min) = 0.537
p = 0.67

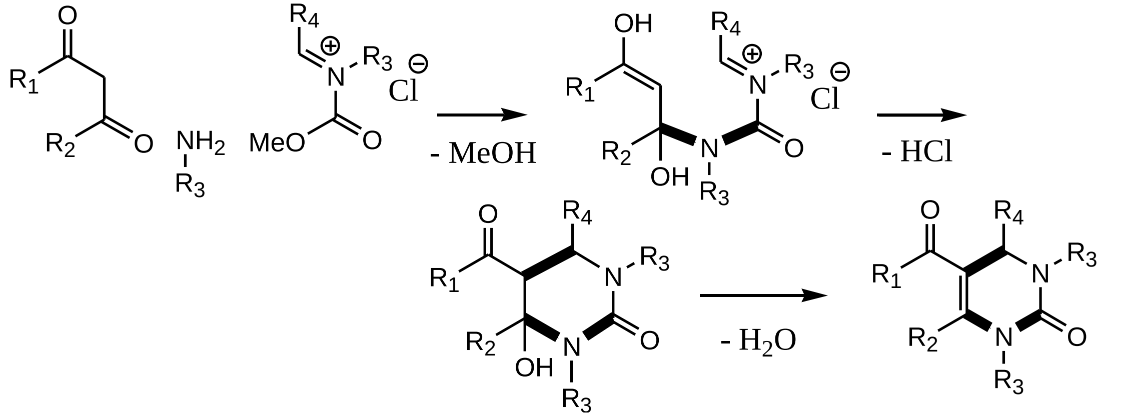

AE(min) = 0.660
p = 0.893

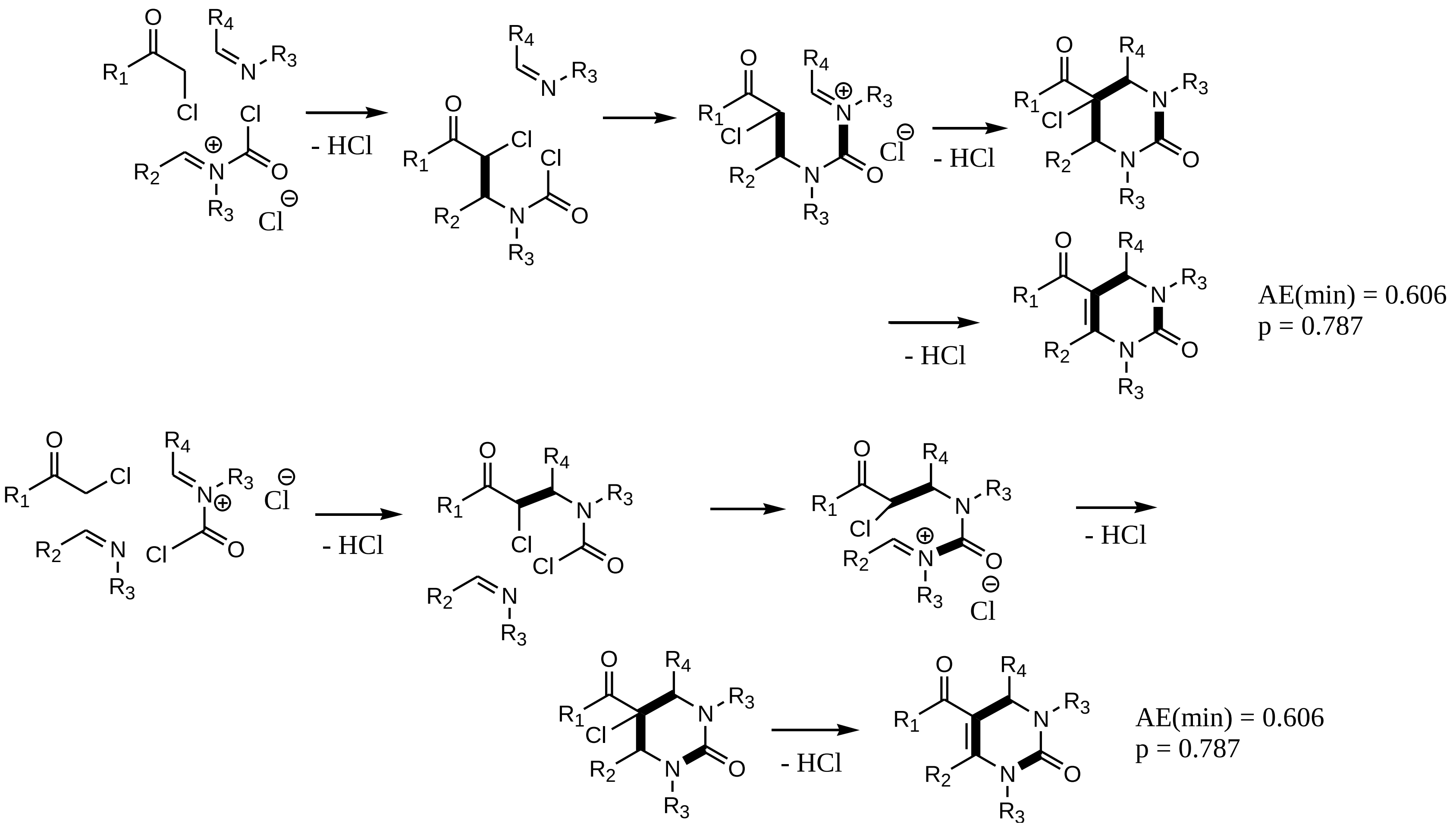

Scheme S4. Conjectured syntheses of the Biginelli adduct via new [3 + 2 + 1] mapping strategies.

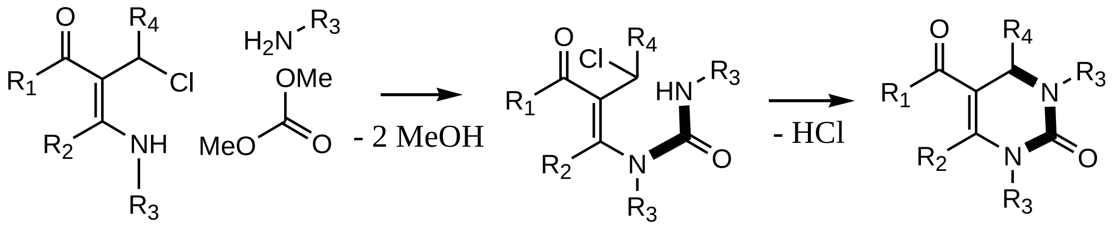

AE(min) = 0.626
p = 0.829

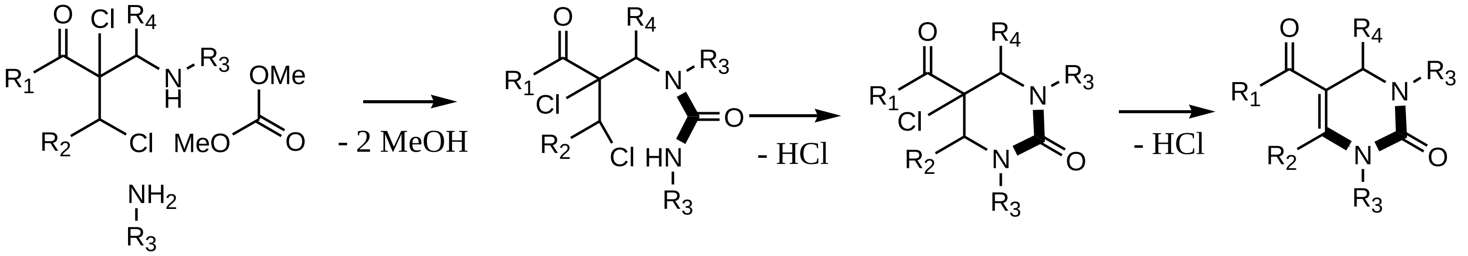

AE(min) = 0.551
p = 0.691

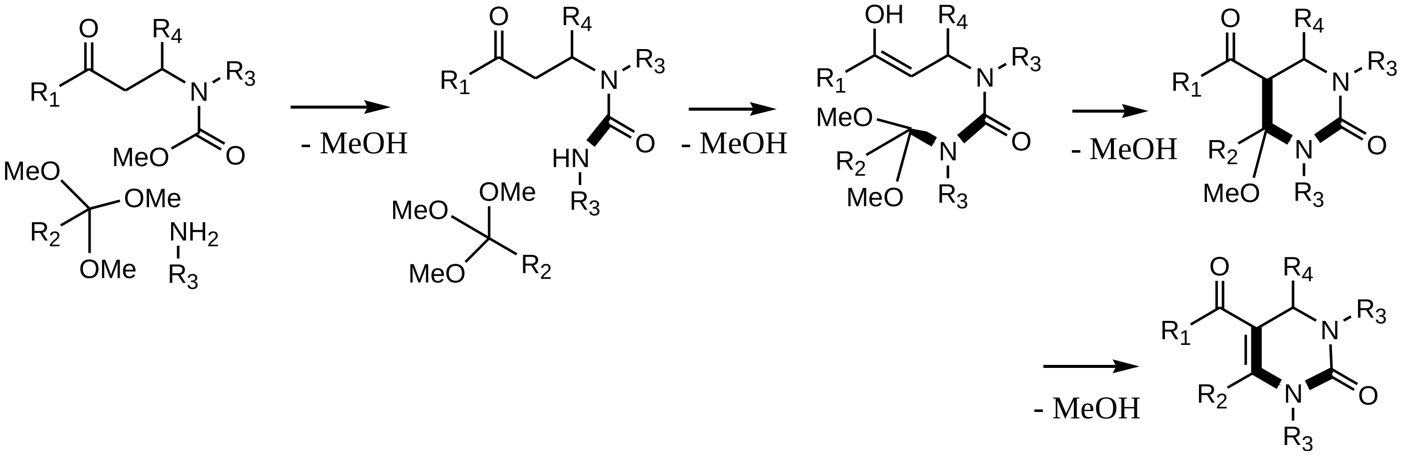

AE(min) = 0.546
p = 0.684

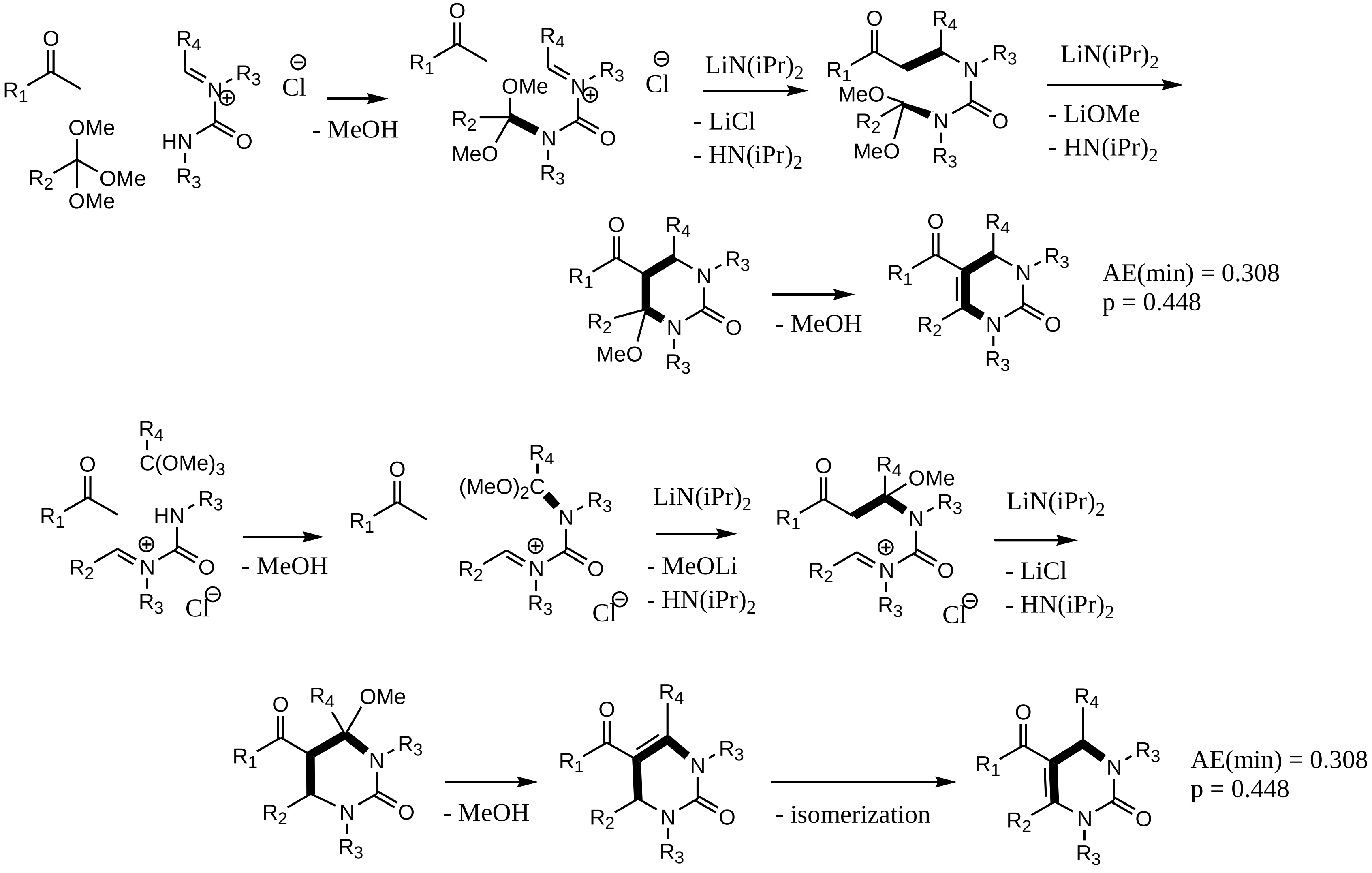

Scheme S5. Conjectured syntheses of the Biginelli adduct via new [4 + 1 + 1] mapping strategies.

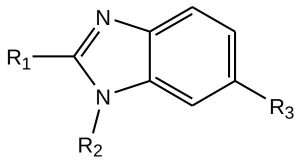

benzimidazole

[2 + 2 + 1]

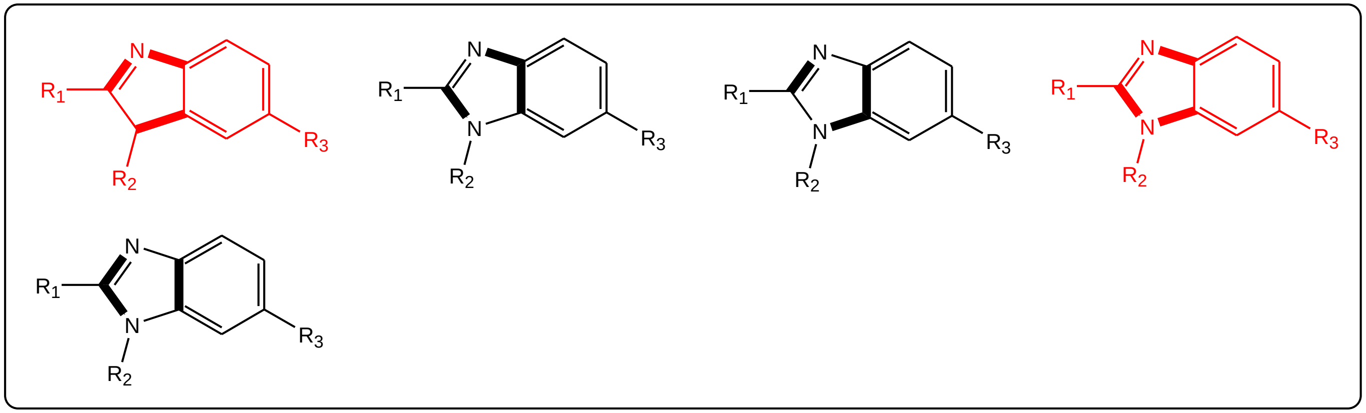

[3 + 1 + 1]

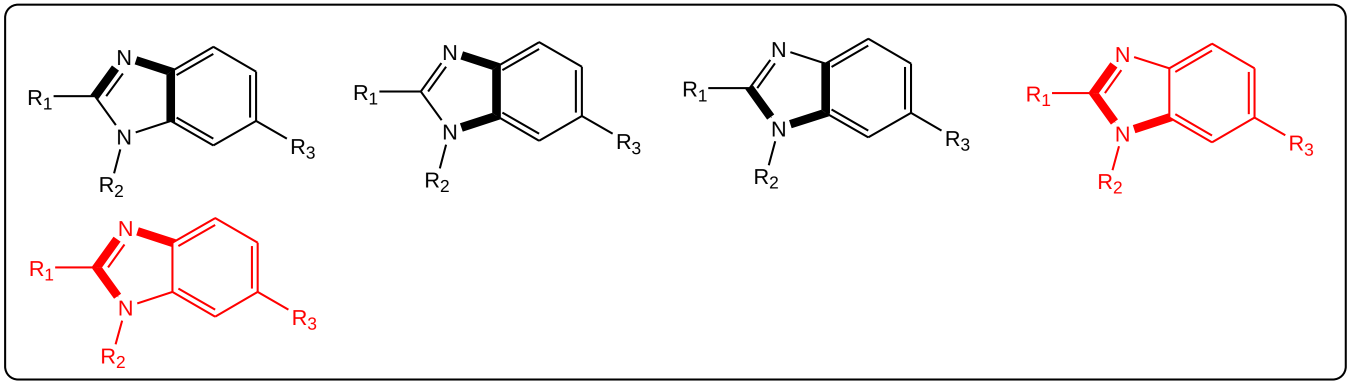

Schemes S6. Superposition of 3-partition templates for benzimidazole.

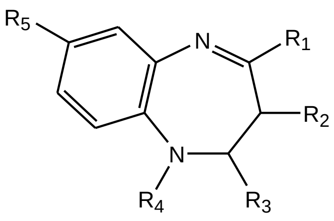

2,3-Dihydro-1*H*-benzo[*b*][1,4]diazepine

three bond cuts

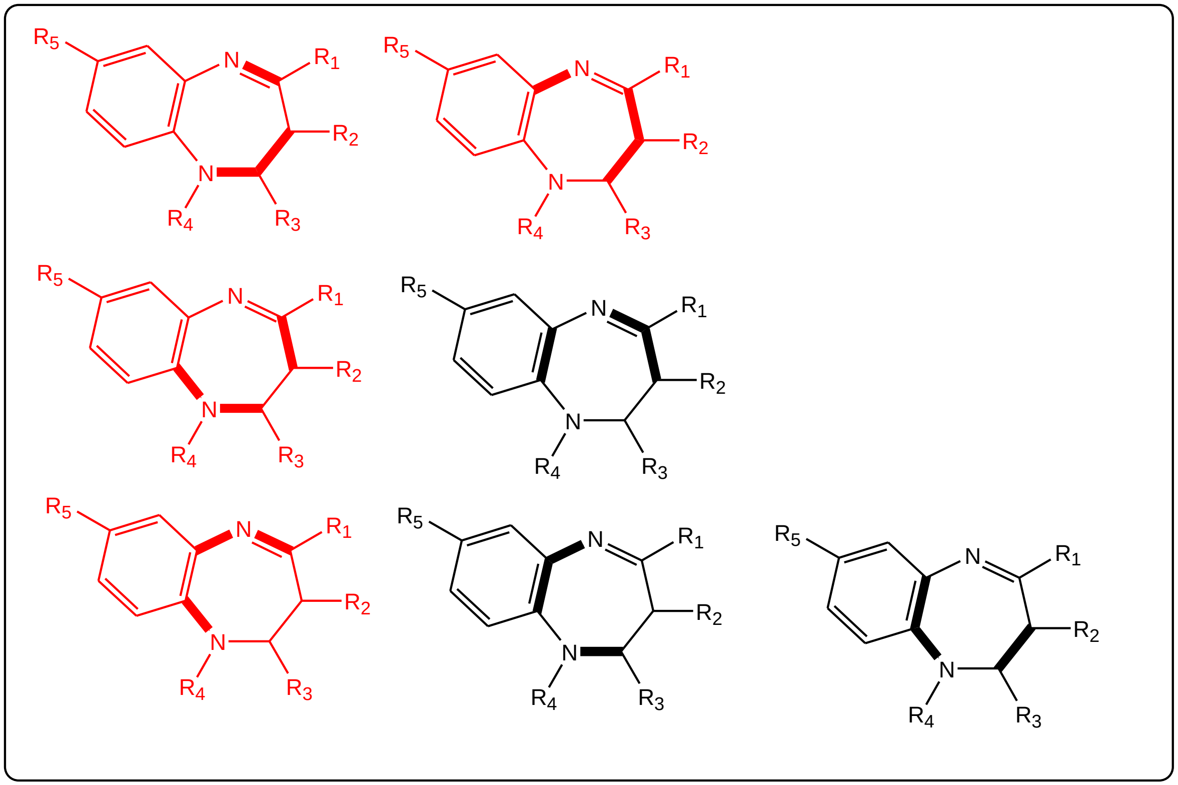

4 + 2 + 1

Schemes S7a. Superposition of [4+2+1] 3-partition templates for benzodiazepine.

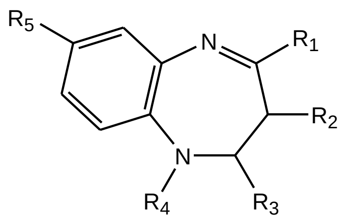

2,3-Dihydro-1*H*-benzo[*b*][1,4]diazepine

three bond cuts

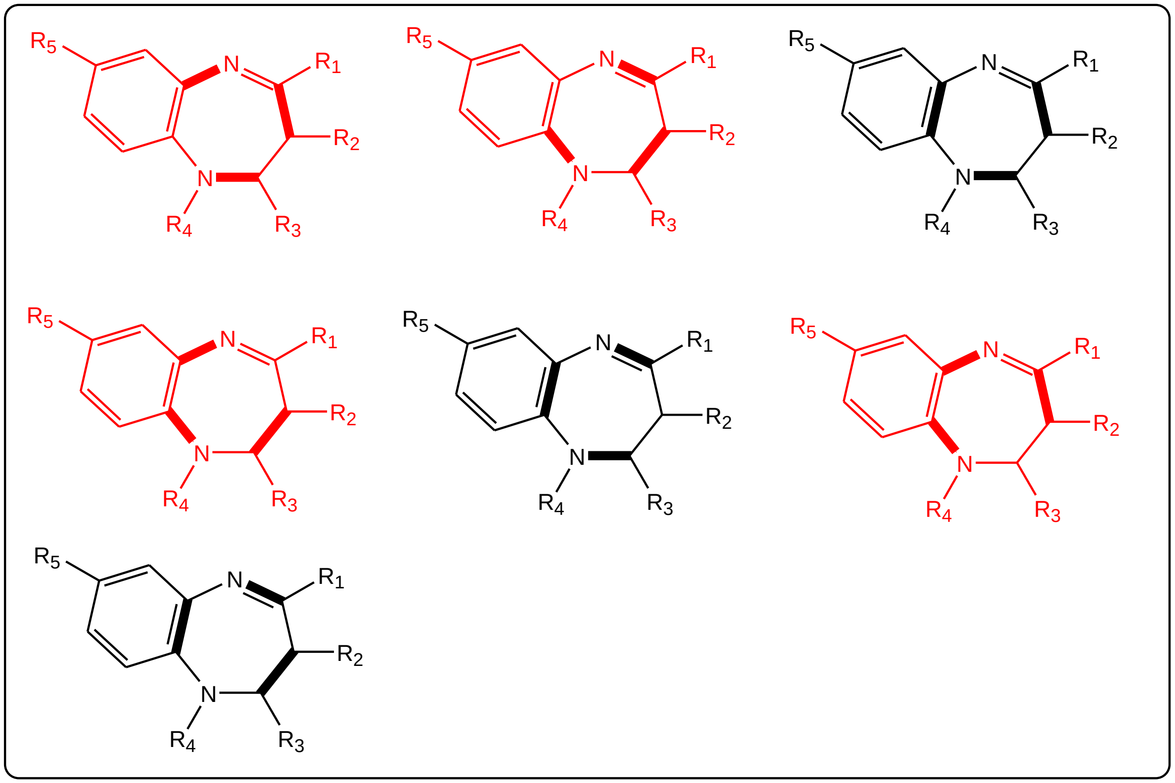

3 + 2 + 2

Schemes S7b. Superposition of [3+2+2] 3-partition templates for benzodiazepine.

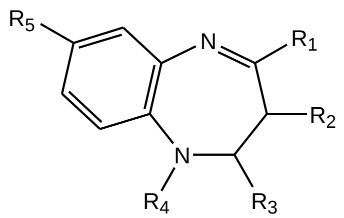

2,3-Dihydro-1*H*-benzo[*b*][1,4]diazepine

three bond cuts

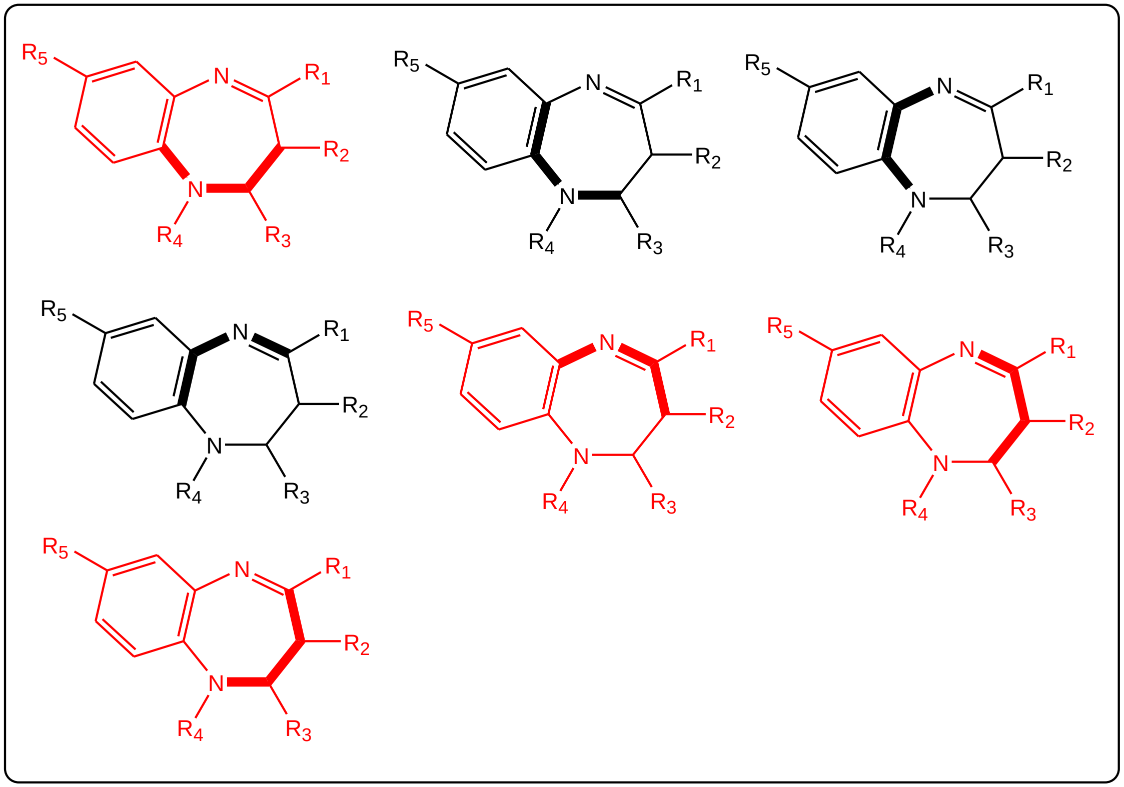

5 + 1 + 1

Schemes S7c. Superposition of [5+1+1] 3-partition templates for benzodiazepine.

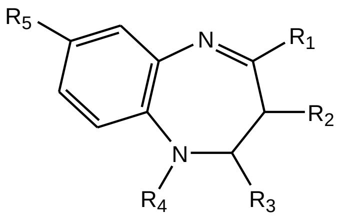

2,3-Dihydro-1*H*-benzo[*b*][1,4]diazepine

<u>three bond cuts</u>

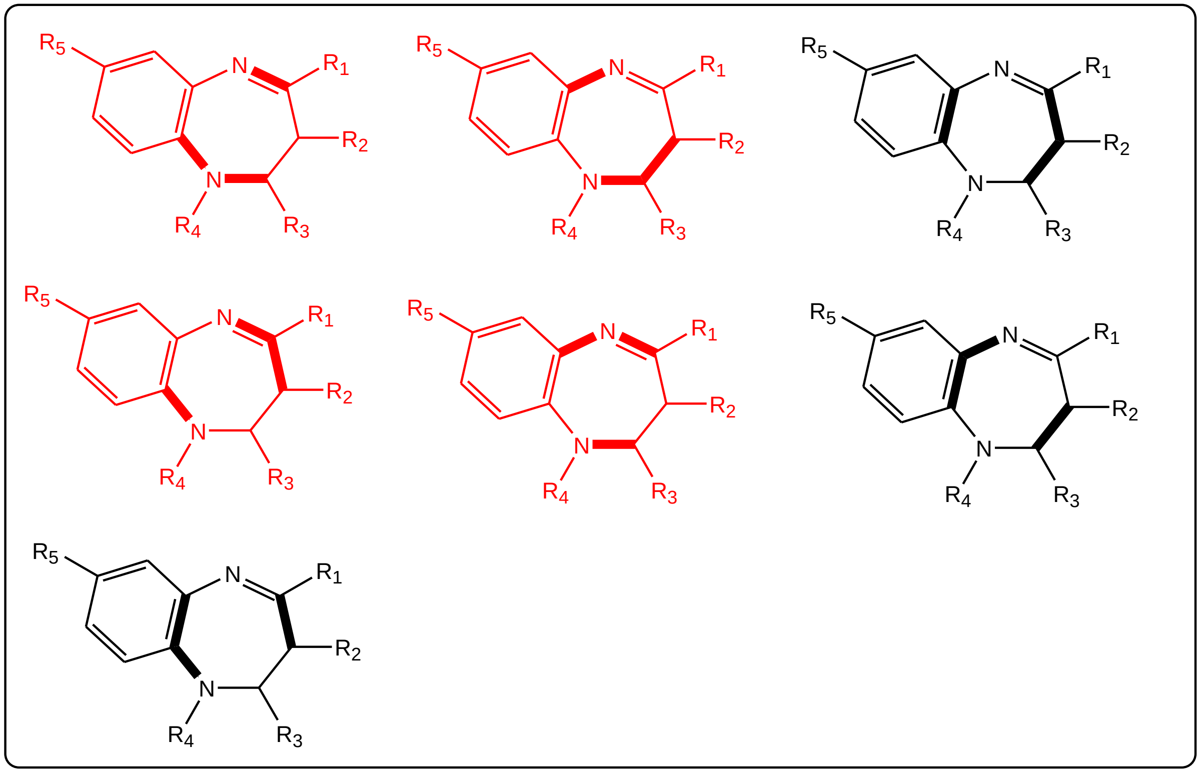

3 + 3 + 1

Schemes S7d. Superposition of [3+3+1] 3-partition templates for benzodiazepine.

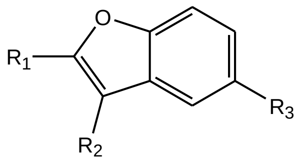

benzofuran

[2 + 2 + 1]

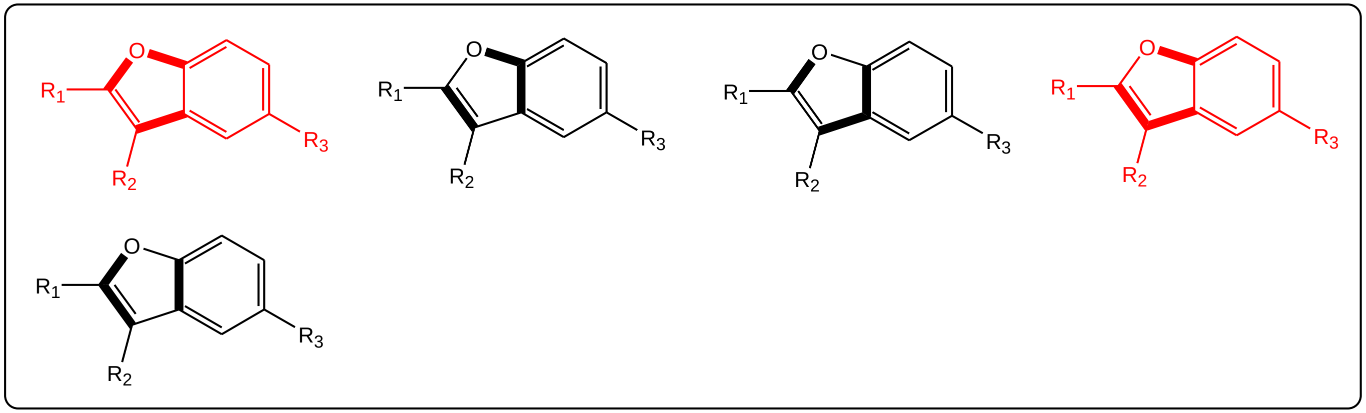

[3 + 1 + 1]

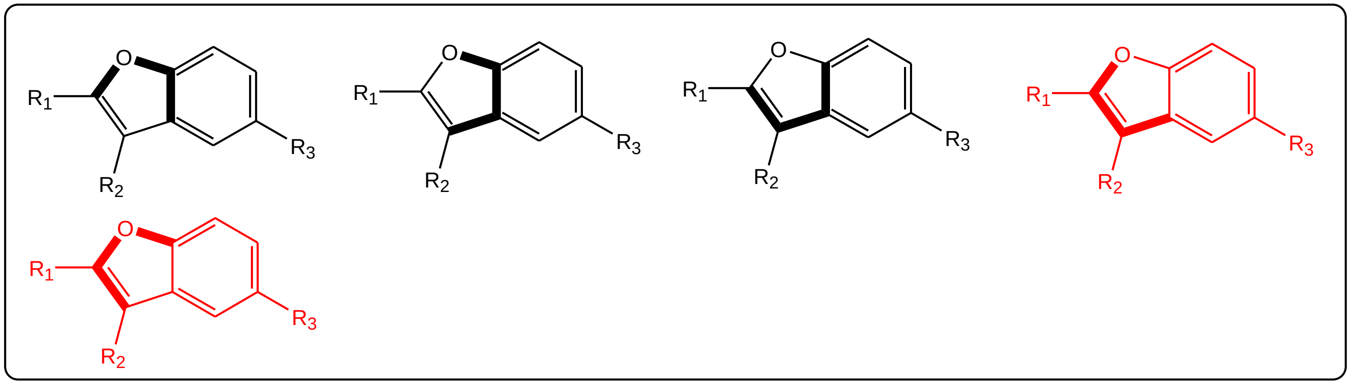

Schemes S8. Superposition of 3-partition templates for benzofuran.

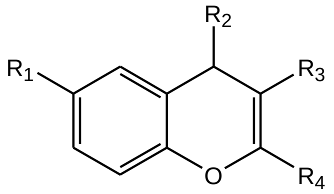

benzopyran

[4 + 1 + 1]

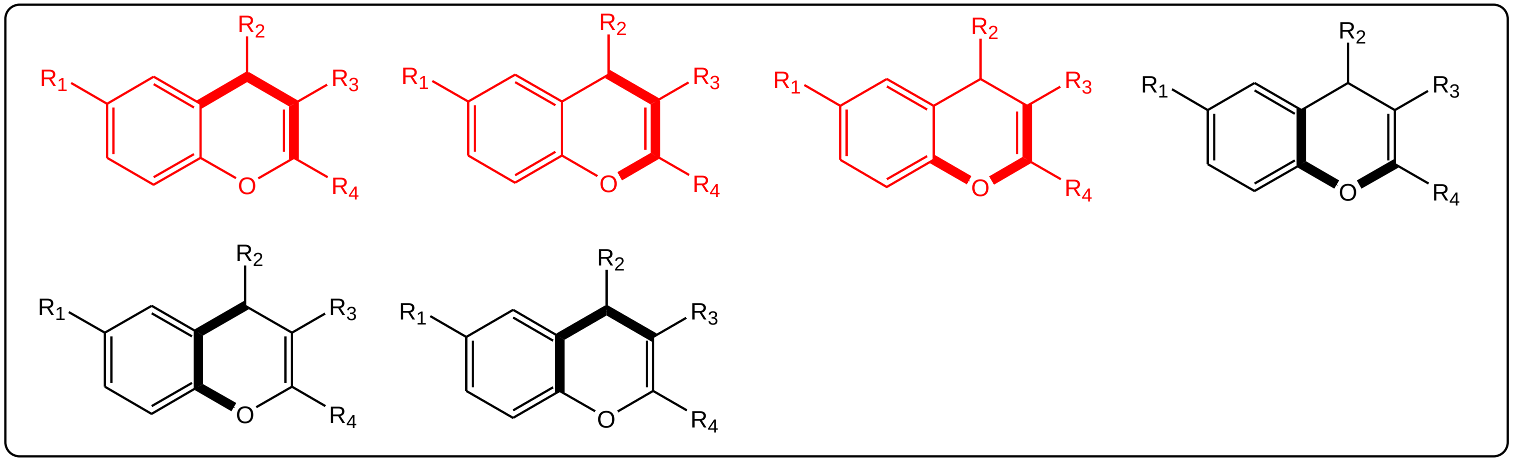

[2 + 2 + 2]

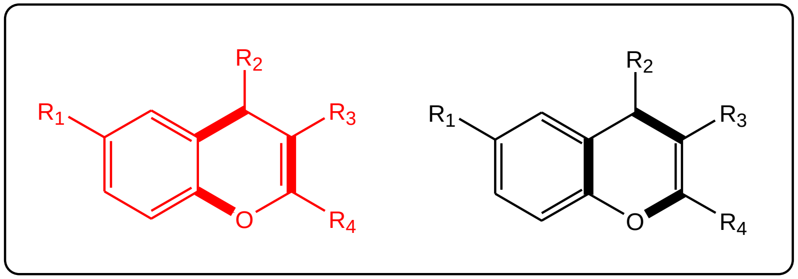

Schemes S9a. Superposition of [4 + 1 + 1] and [2 + 2 + 2] 3-partition templates for benzofuran.

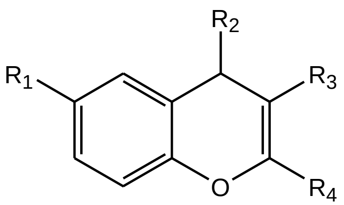

benzopyran

[3 + 2 + 1]

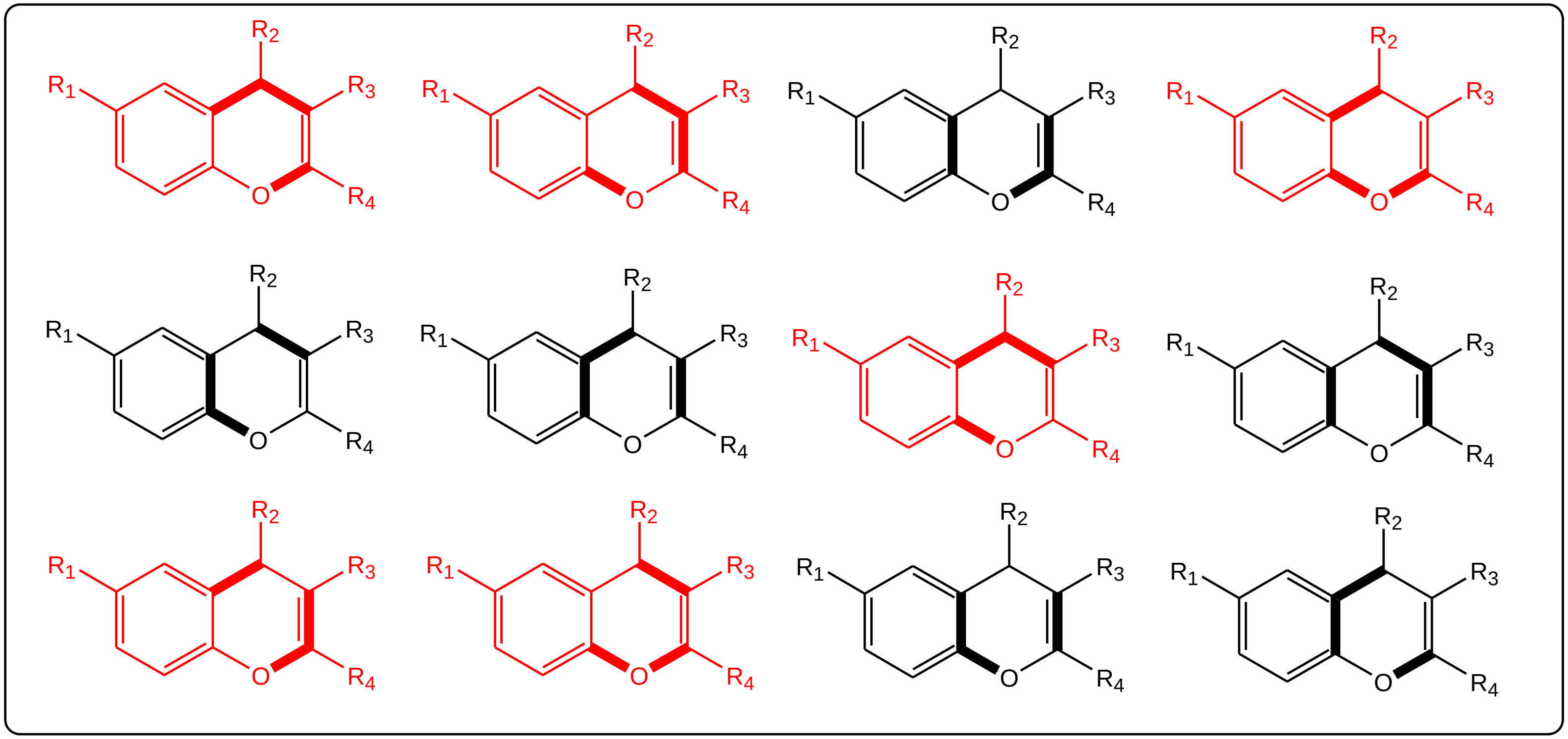

Schemes S9b. Superposition of [3 + 2 + 1] 3-partition templates for benzofuran.

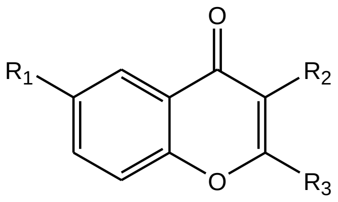

chromene-4-one

[4 + 1 + 1]

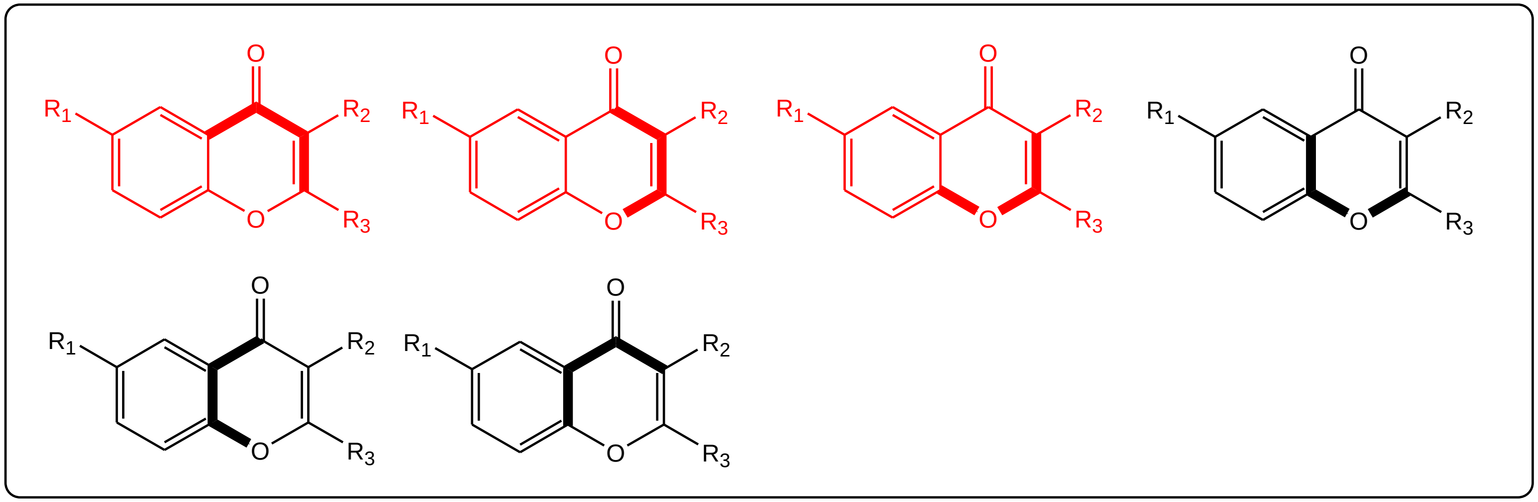

[2 + 2 + 2]

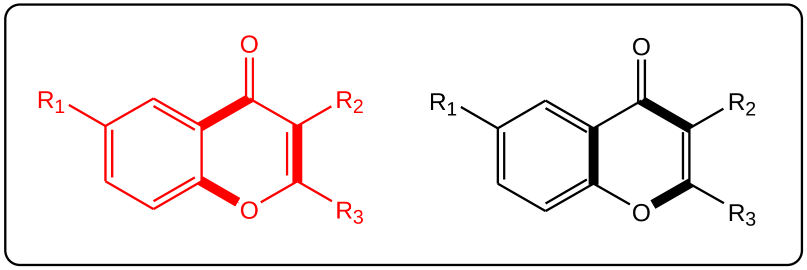

Schemes S10a. Superposition of [4 + 1 + 1] and [2 + 2 + 2] 3-partition templates for chromene-4-one.

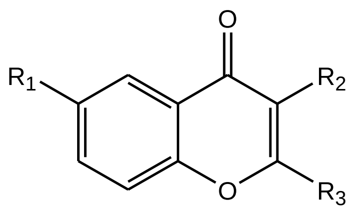

chromene-4-one

[3 + 2 + 1]

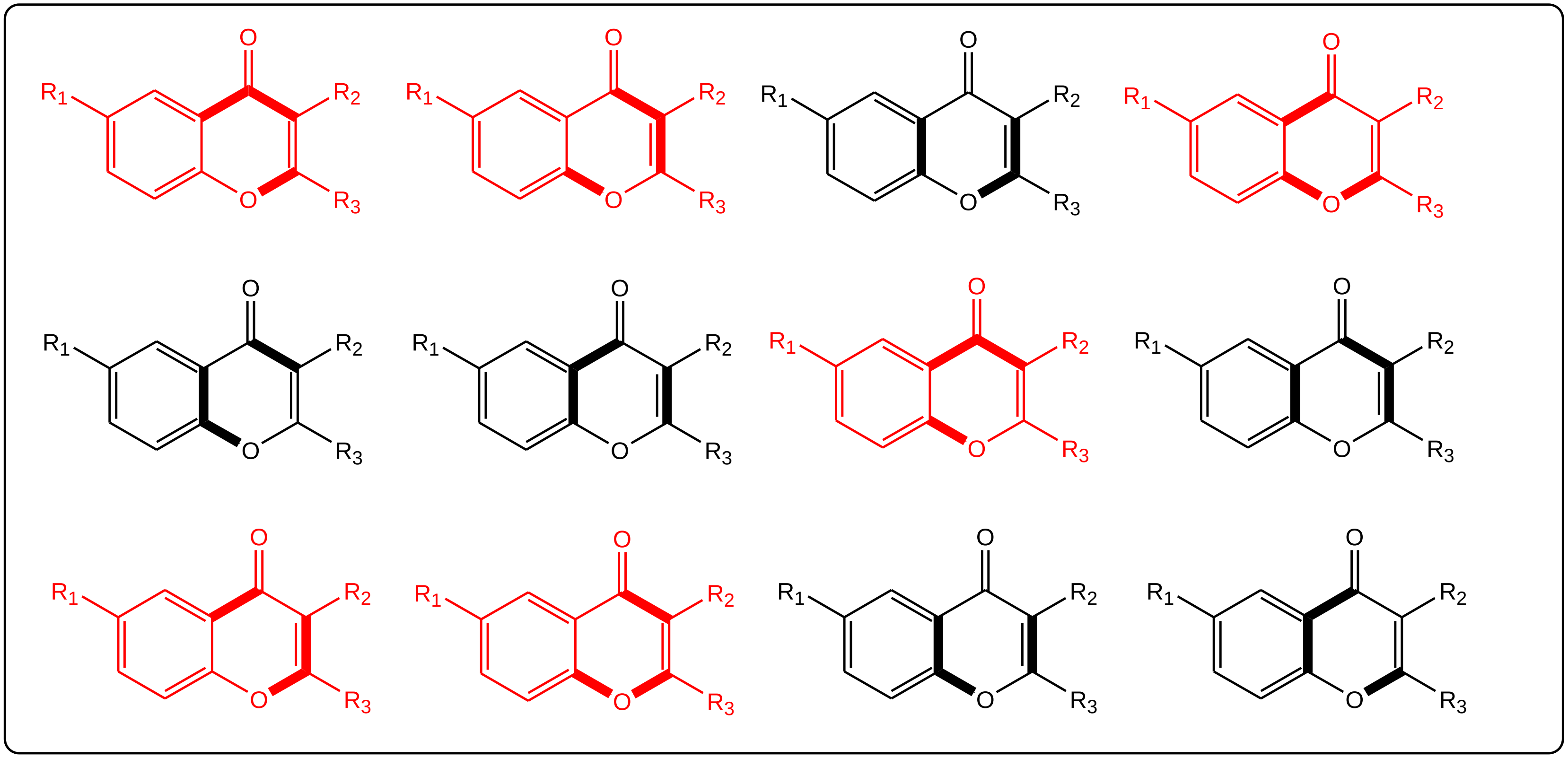

Schemes S10b. Superposition of [3 + 2 + 1] 3-partition templates for chromene-4-one.

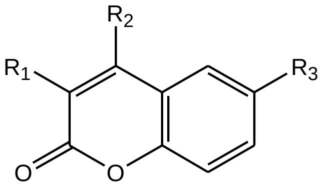

coumarin

[4 + 1 + 1]

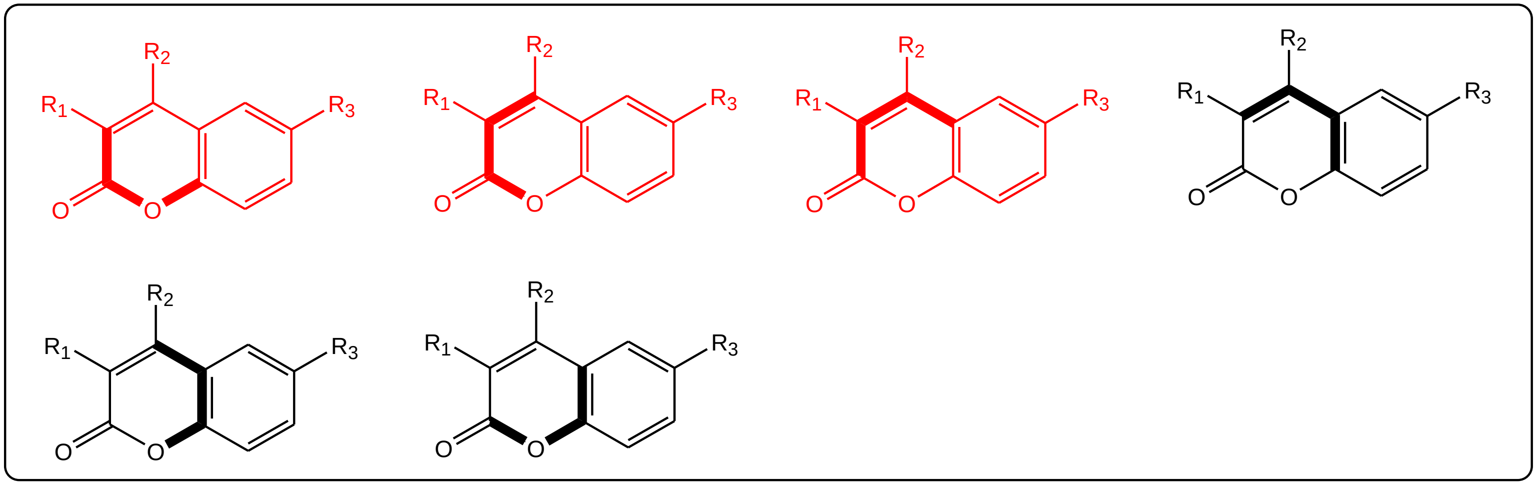

[2 + 2 + 2]

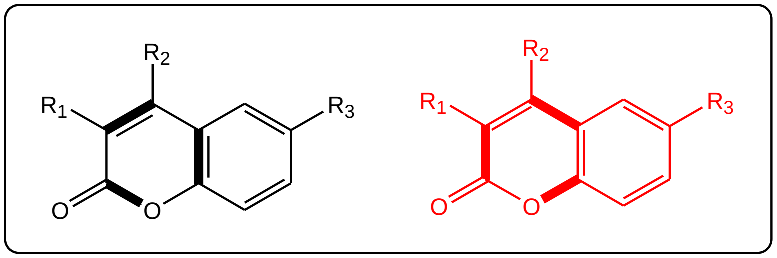

Schemes S11a. Superposition of [4 + 1 + 1] and [2 + 2 + 2] 3-partition templates for coumarin.

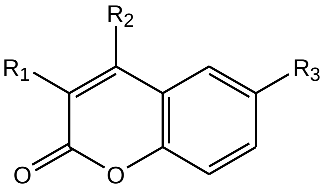

coumarin

3 + 2 + 1

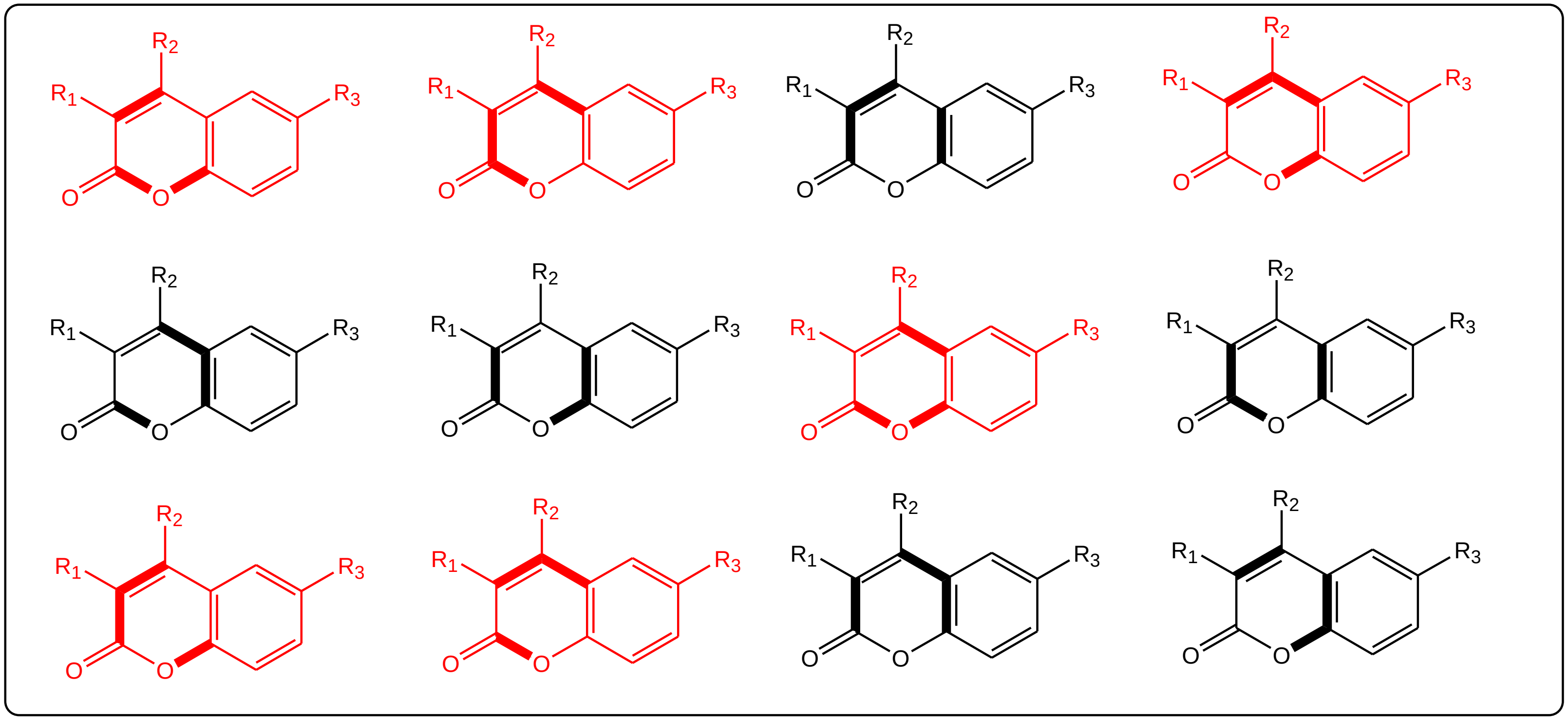

Schemes S11b. Superposition of [3 + 2 + 1] 3-partition templates for coumarin.

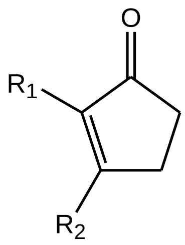

cyclopent-2-enone

<u>three bond cuts</u>

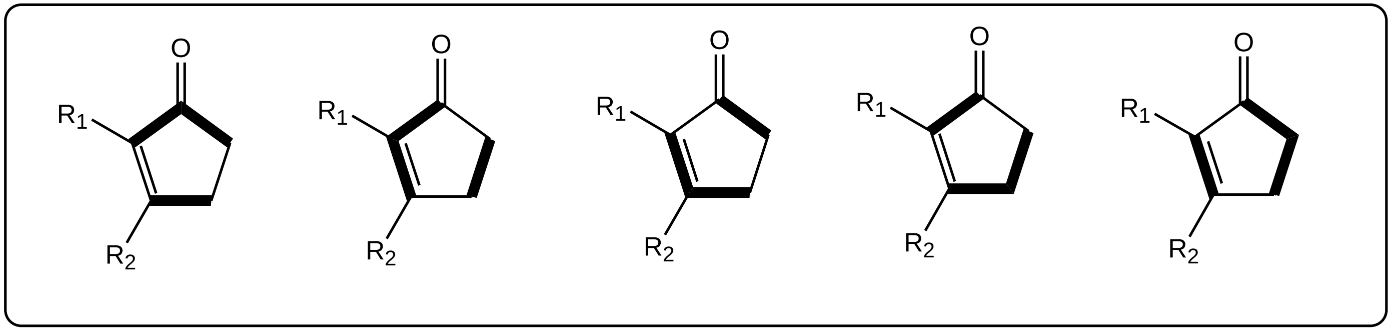

2 + 2 + 1

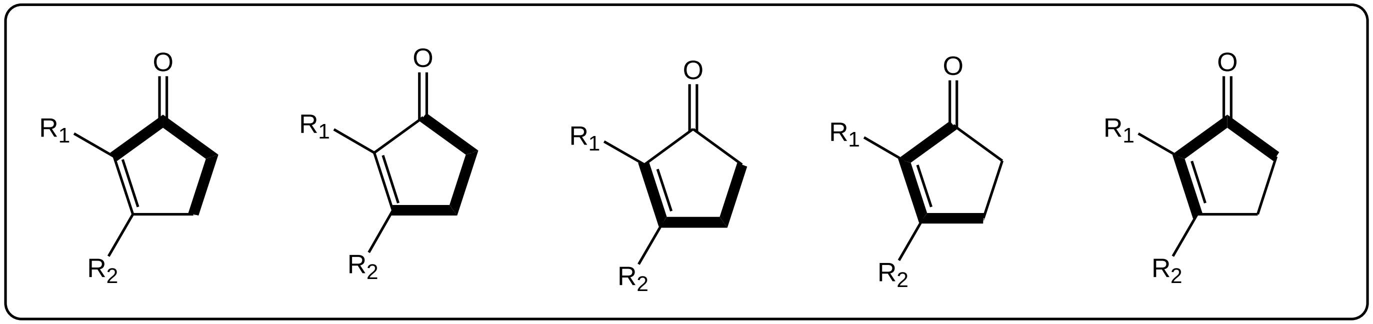

3 + 1 + 1

Schemes S12. Superposition of 3-partition templates for cyclopent-2-enone.

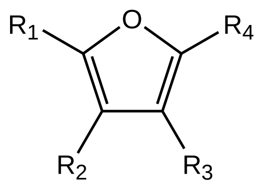

furan

three bond cuts

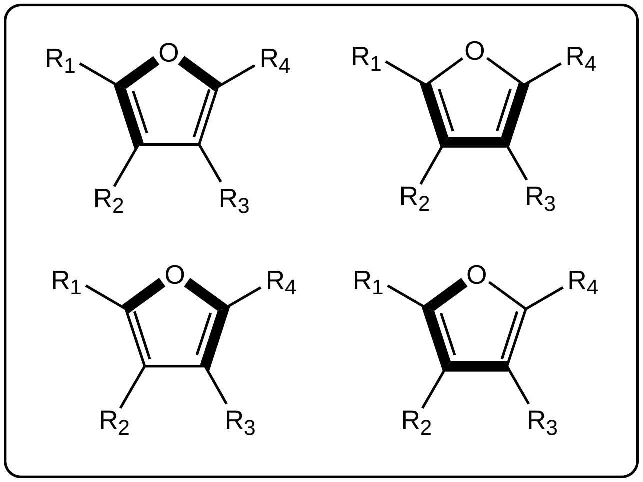

[3 + 1 + 1]

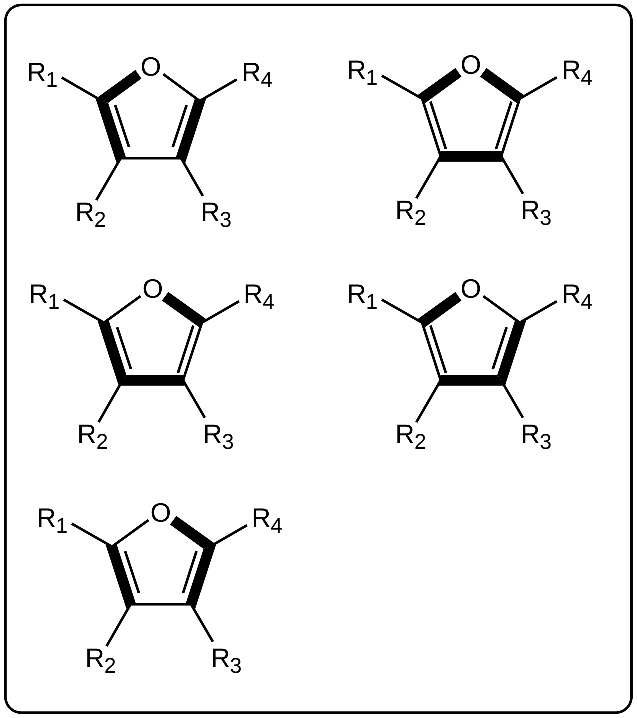

[2 + 2 + 1]

Schemes S13. Superposition of 3-partition templates for furan.

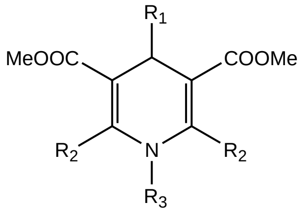

Hantzsch dihydropyridine

<u>three bond cuts</u> 4 + 1 + 1

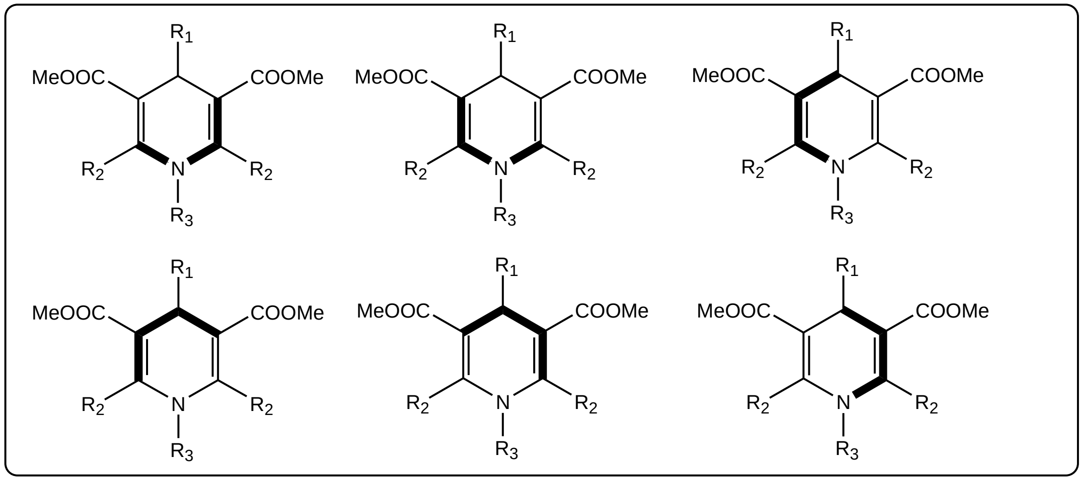

2 + 2 + 2

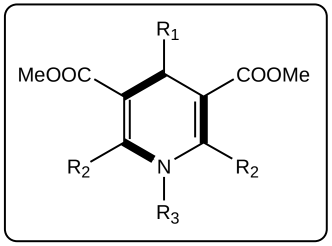

Schemes S14a. Superposition of [4 + 1 + 1] and [2 + 2 + 2] 3-partition templates for Hantzsch dihydropyridine.

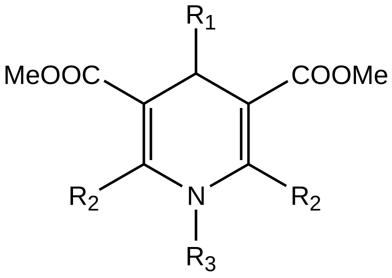

Hantzsch dihydropyridine

<u>three bond cuts</u>

3 + 2 + 1

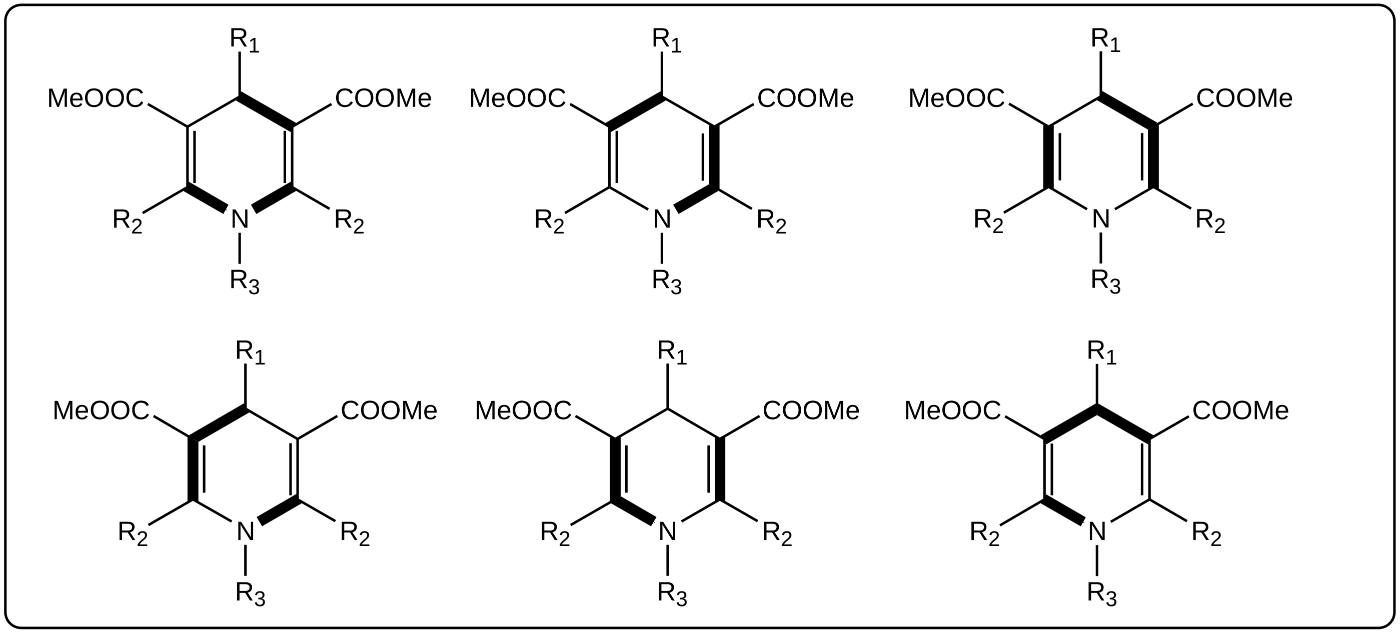

Schemes S14b. Superposition of [3 + 2 + 1] 3-partition templates for Hantzsch dihydropyridine.

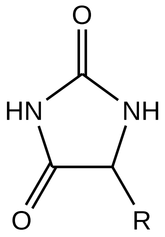

hydantoin

<u>three bond cuts</u>

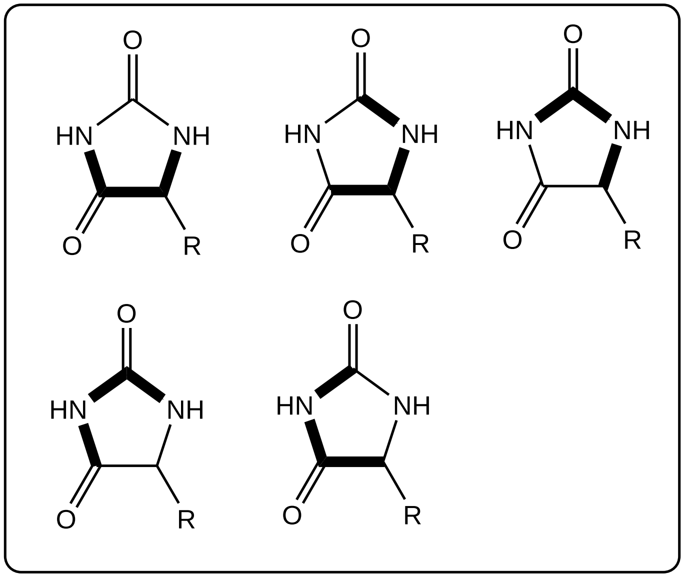

[3 + 1 + 1]

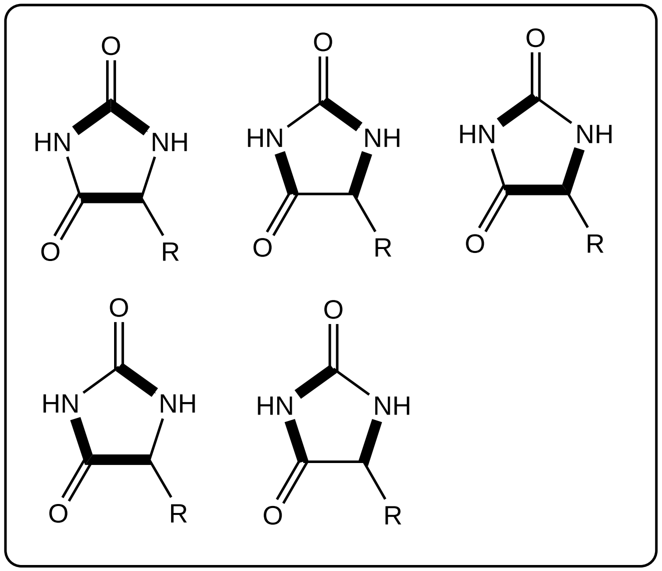

[2 + 2 + 1]

Schemes S15. Superposition of 3-partition templates for hydantoin.

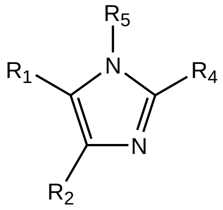

imidazole

three bond cuts

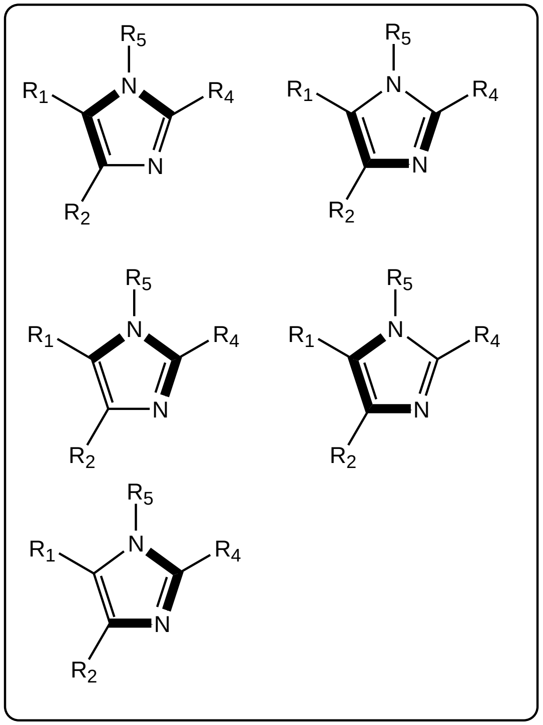

[3 + 1 + 1]

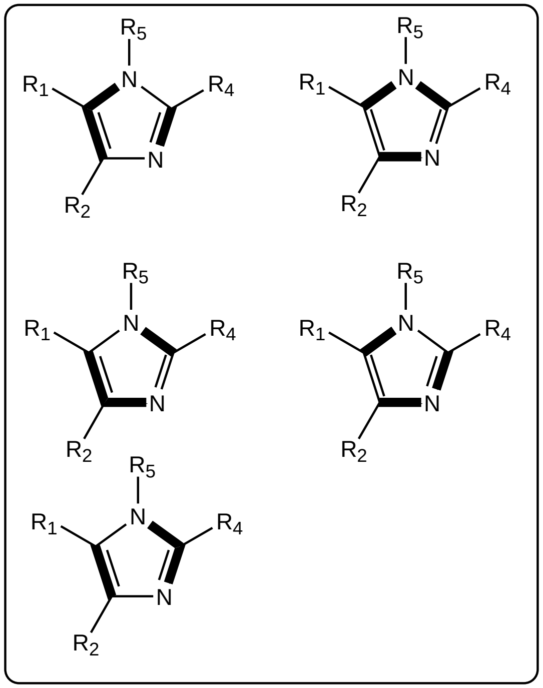

[2 + 2 + 1]

Schemes S16. Superposition of 3-partition templates for imidazole.

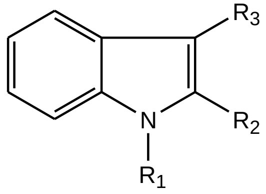

indole

<u>three bond cuts:</u>

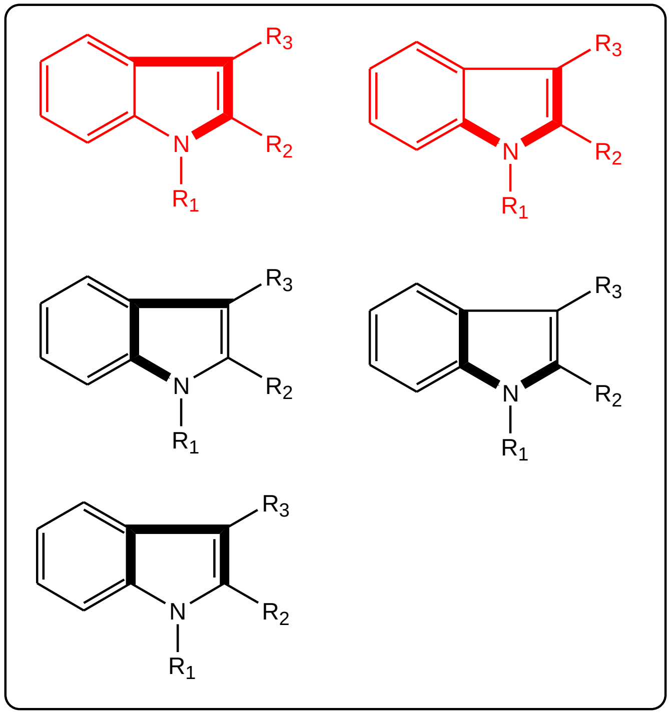

[3 + 1 + 1]

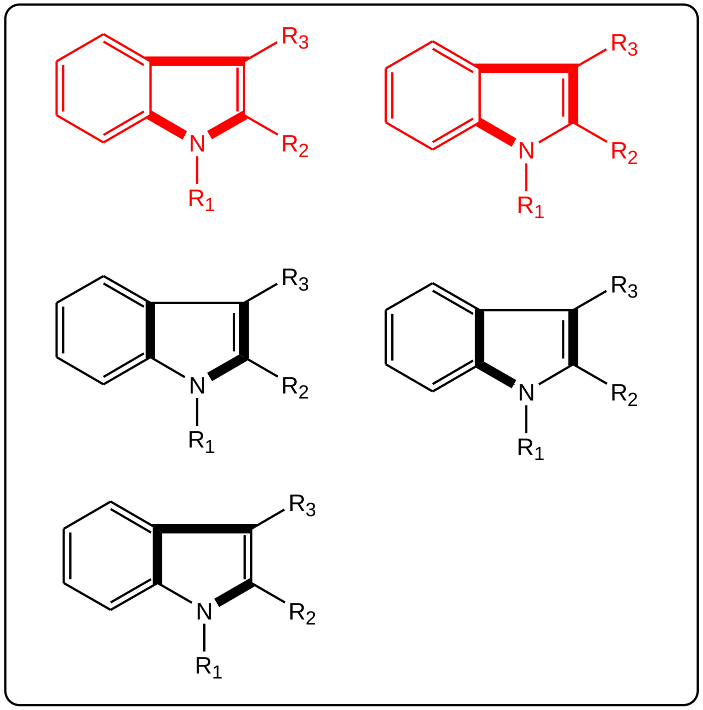

[2 + 2 + 1]

Schemes S17. Superposition of 3-partition templates for indole.

isoquinoline 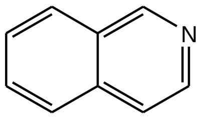

[4+1+1]

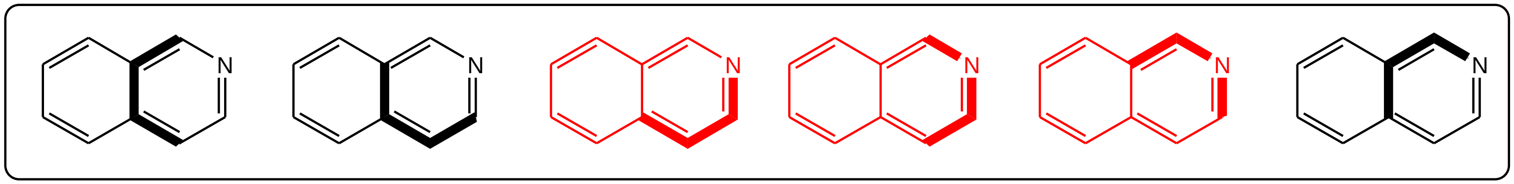

[3+2+1]

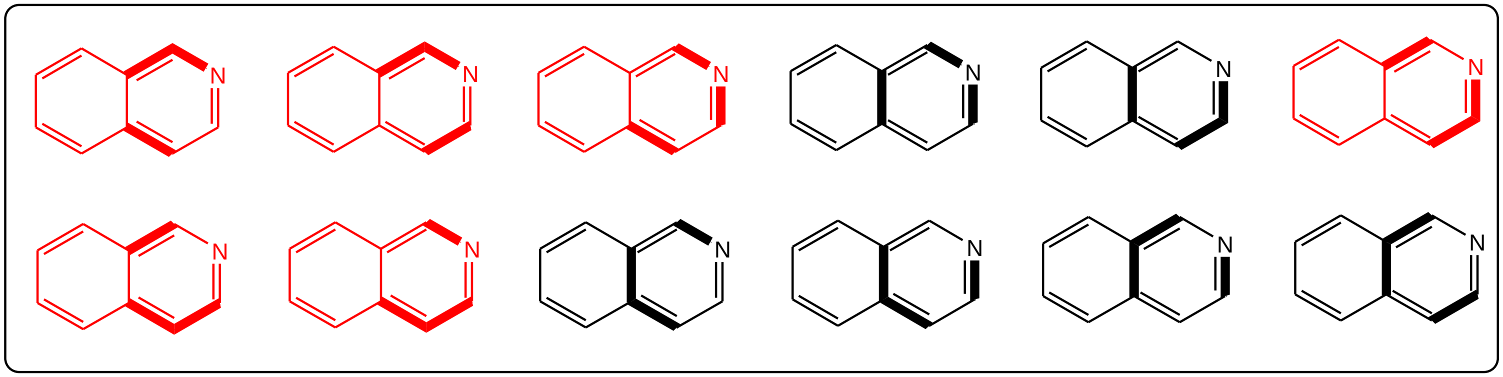

[2+2+2]

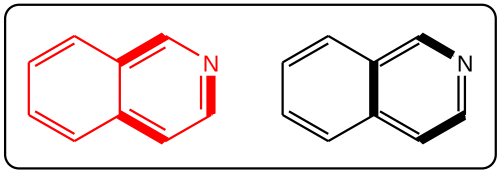

Schemes S18. Superposition of 3-partition templates for isoquinoline.

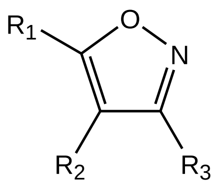

isoxazole

three bond cuts

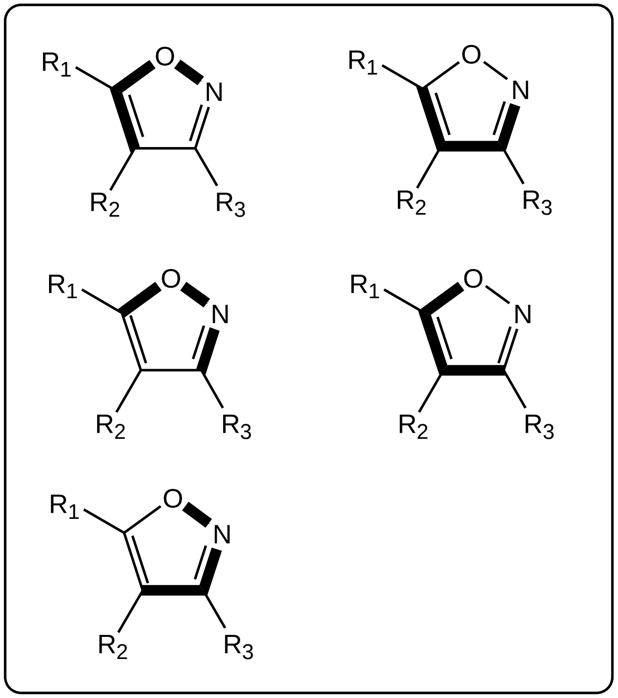

[3 + 1 + 1]

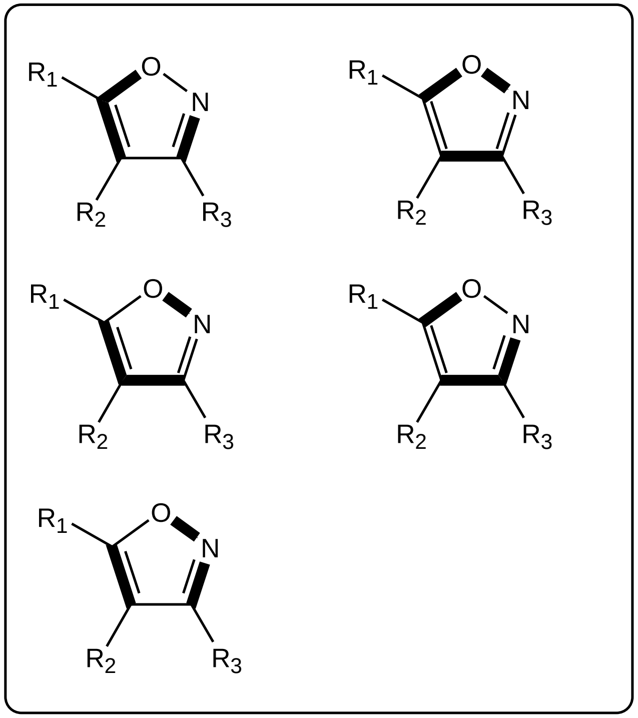

[2 + 2 + 1]

Schemes S19. Superposition of 3-partition templates for isoxazole.

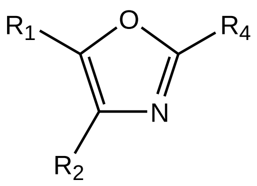

oxazole

<u>three bond cuts</u>

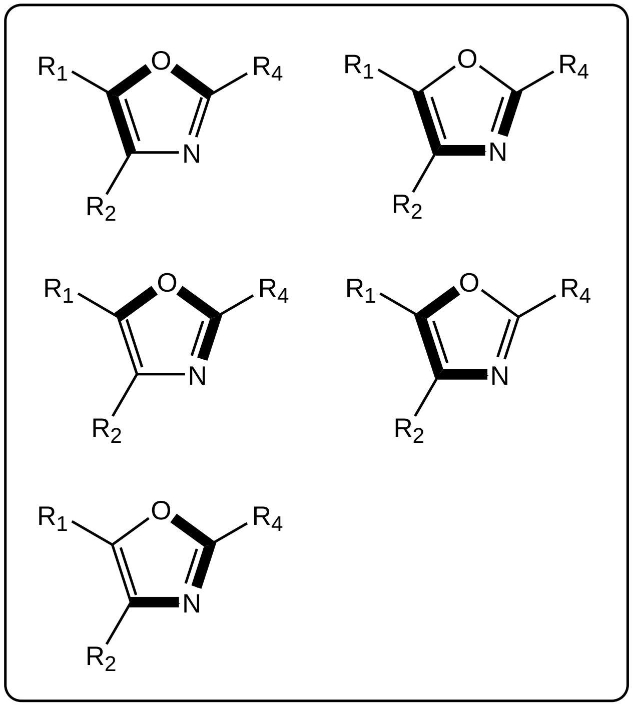

[3 + 1 + 1]

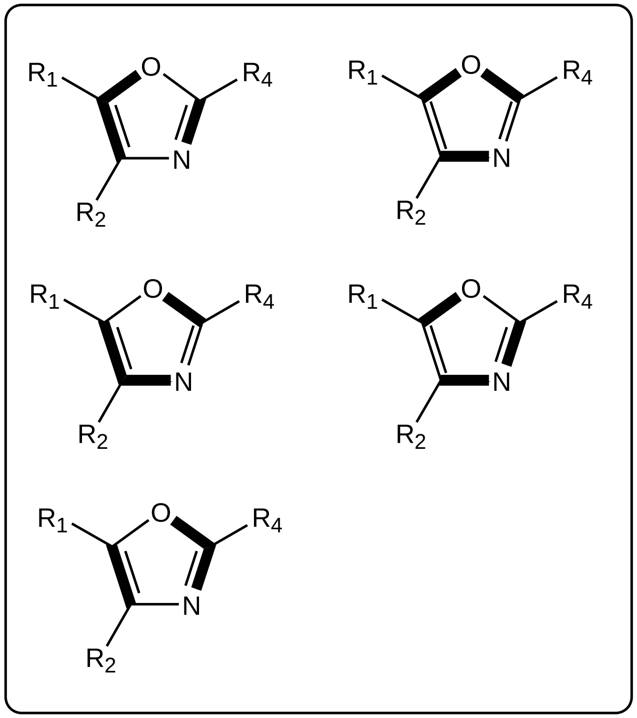

[2 + 2 + 1]

Schemes S20. Superposition of 3-partition templates for oxazole.

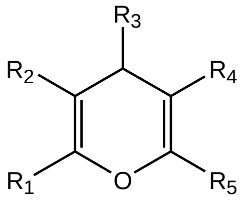

4*H*-Pyran

2 + 2 + 2

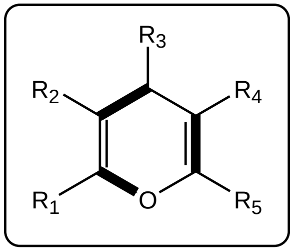

4 + 1 + 1

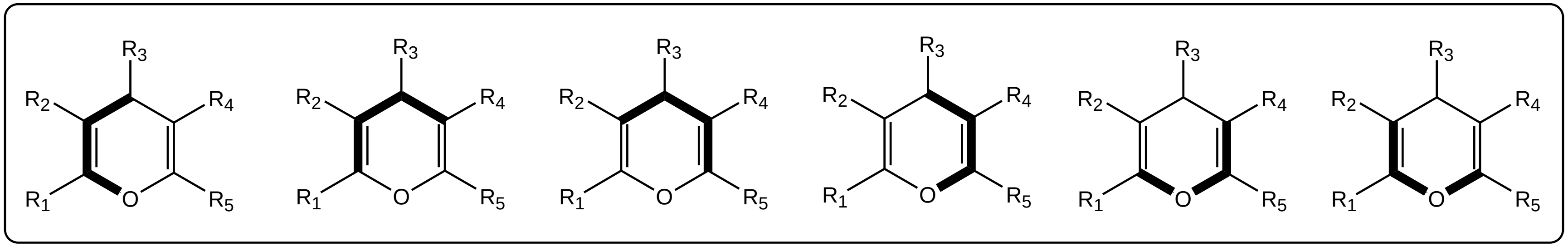

3 + 2 + 1

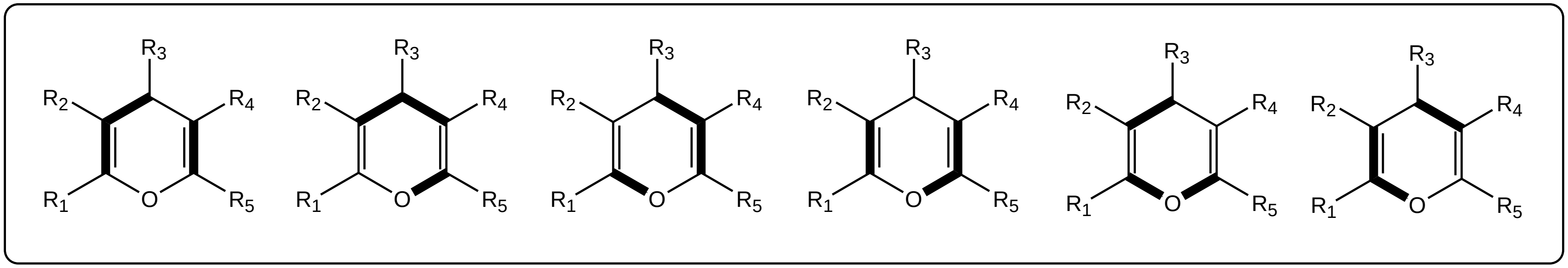

Schemes S21. Superposition of 3-partition templates for pyran.

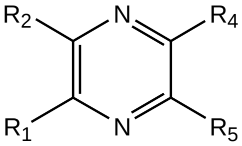

pyrazine

[2+2+2]

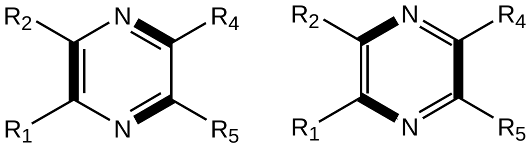

[3+2+1]

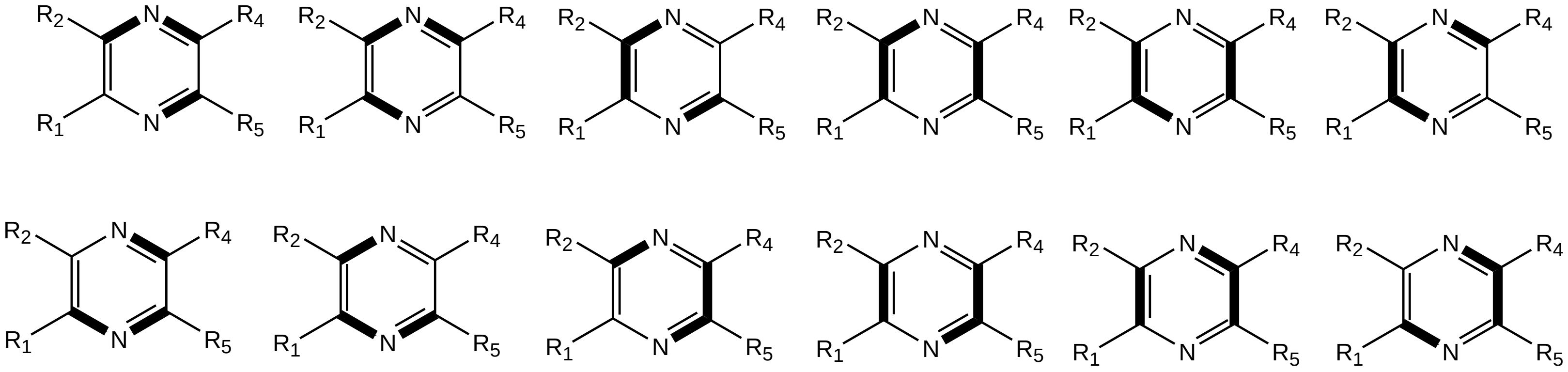

Schemes S22a. Superposition of [2 + 2 + 2] and [3 + 2 + 1] 3-partition templates for pyrazine.

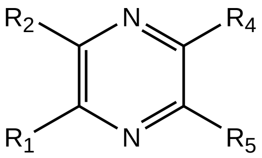

pyrazine

[4+1+1]

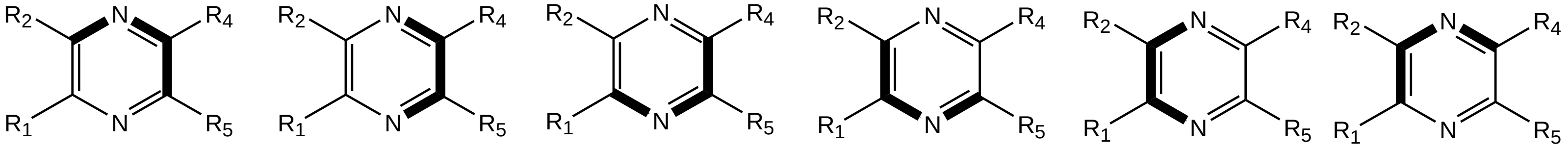

Schemes S22b. Superposition of [4 + 1 + 1] 3-partition templates for pyrazine.

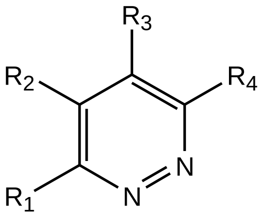

pyridazine

[4+1+1]

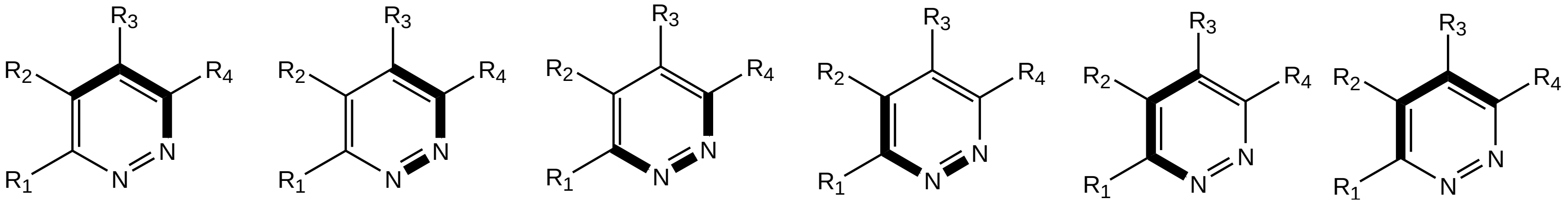

Schemes S23a. Superposition of [4 + 1 + 1] 3-partition templates for pyridazine.

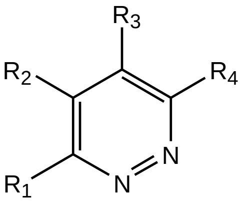

pyridazine

[2+2+2]

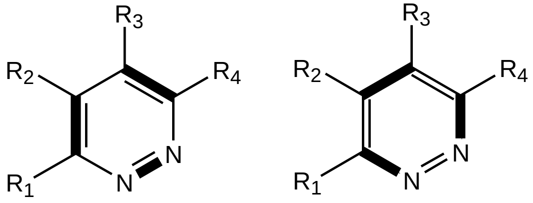

[3+2+1]

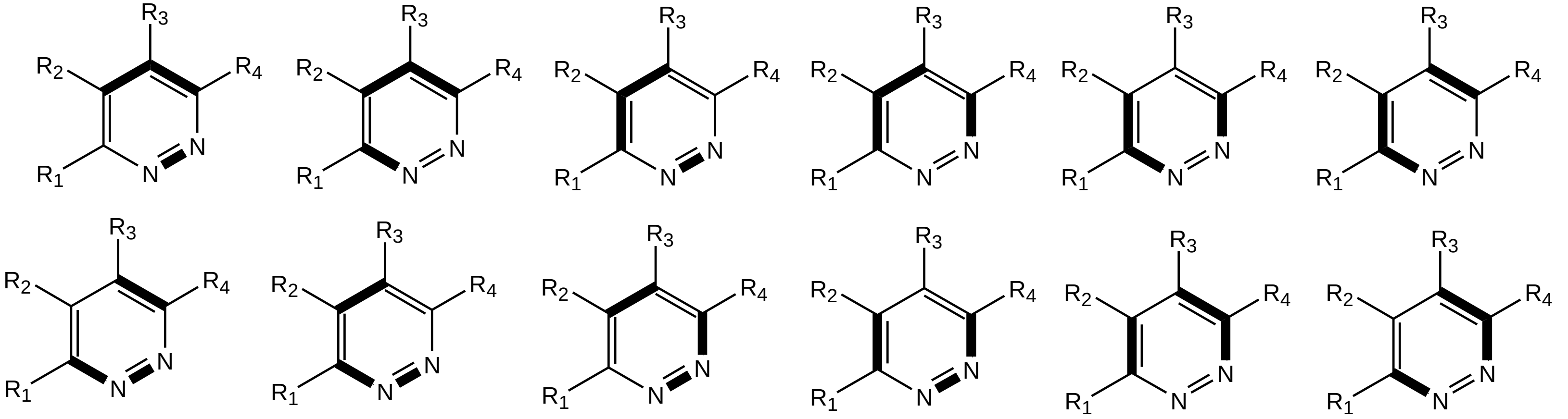

Schemes S23b. Superposition of [2 + 2 + 2] and [3 + 2 + 1] 3-partition templates for pyridazine.

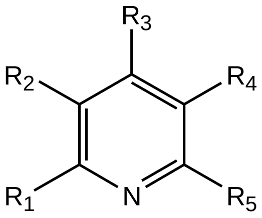

pyridine

[4+1+1]

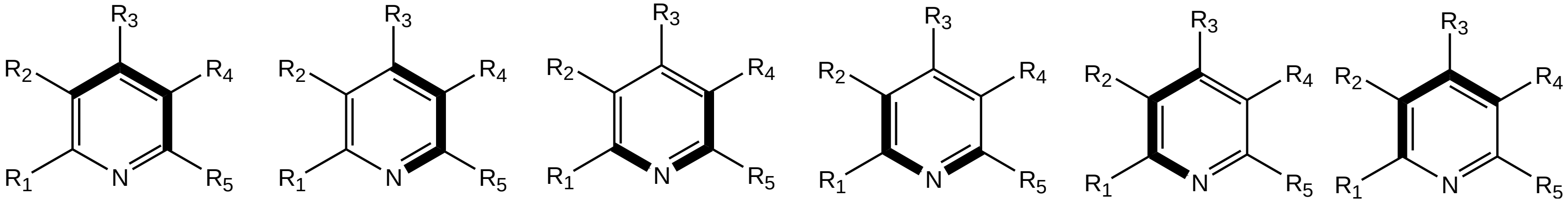

[2+2+2]

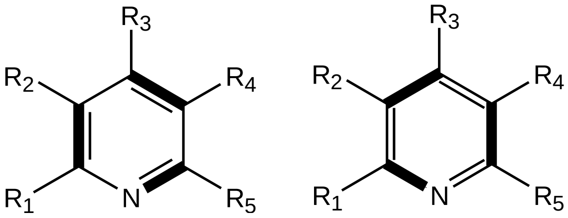

Schemes S24a. Superposition of [2 + 2 + 2] and [4 + 1 + 1] 3-partition templates for pyridine.

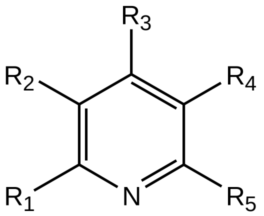

pyridine

[3+2+1]

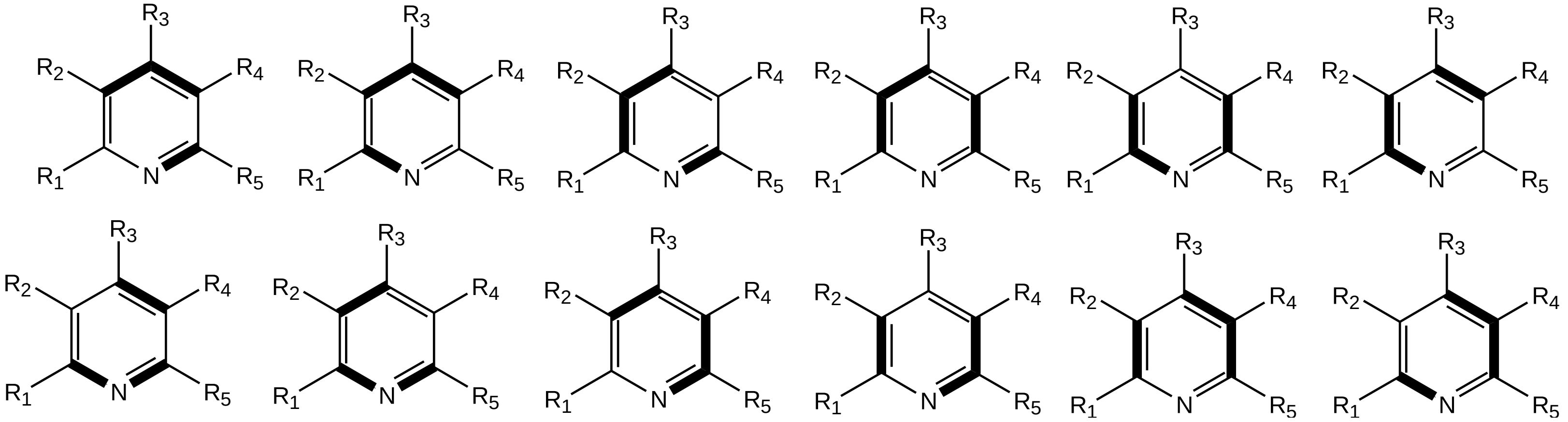

Schemes S24b. Superposition of [3 + 2 + 2] 3-partition templates for pyridine.

pyridinone

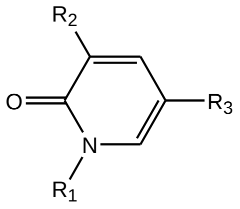

[2+2+2]

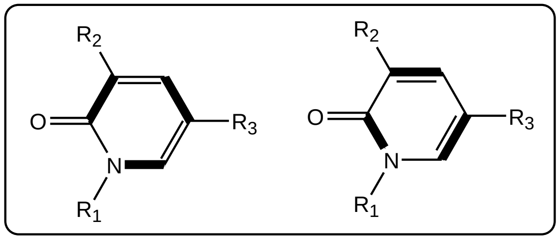

[4+1+1]

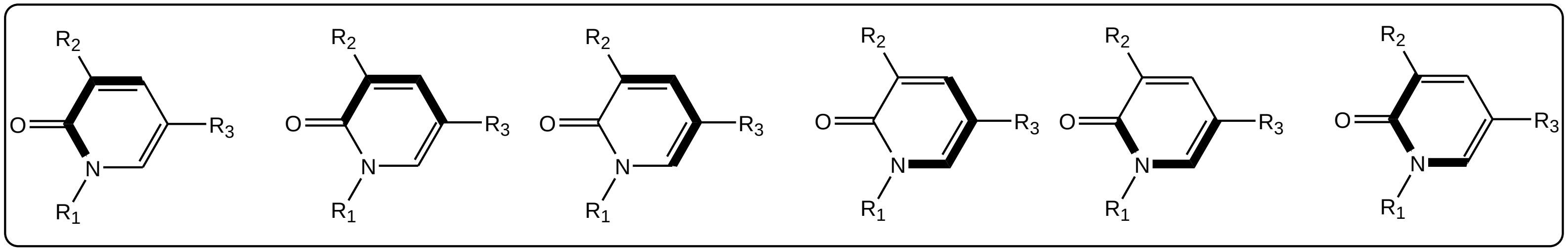

[3+2+1]

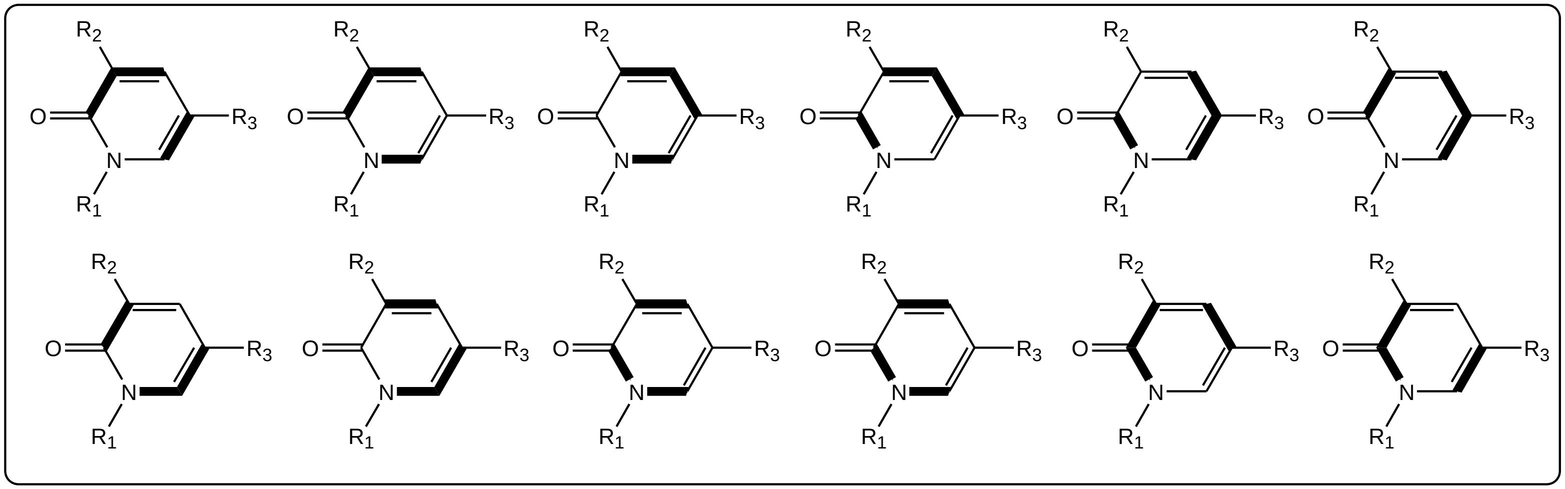

Schemes S25. Superposition of 3-partition templates for pyridinone.

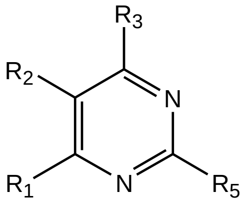

pyrimidine

[2+2+2]

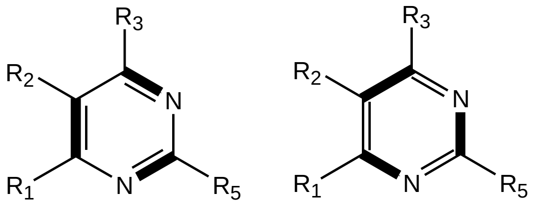

[3+2+1]

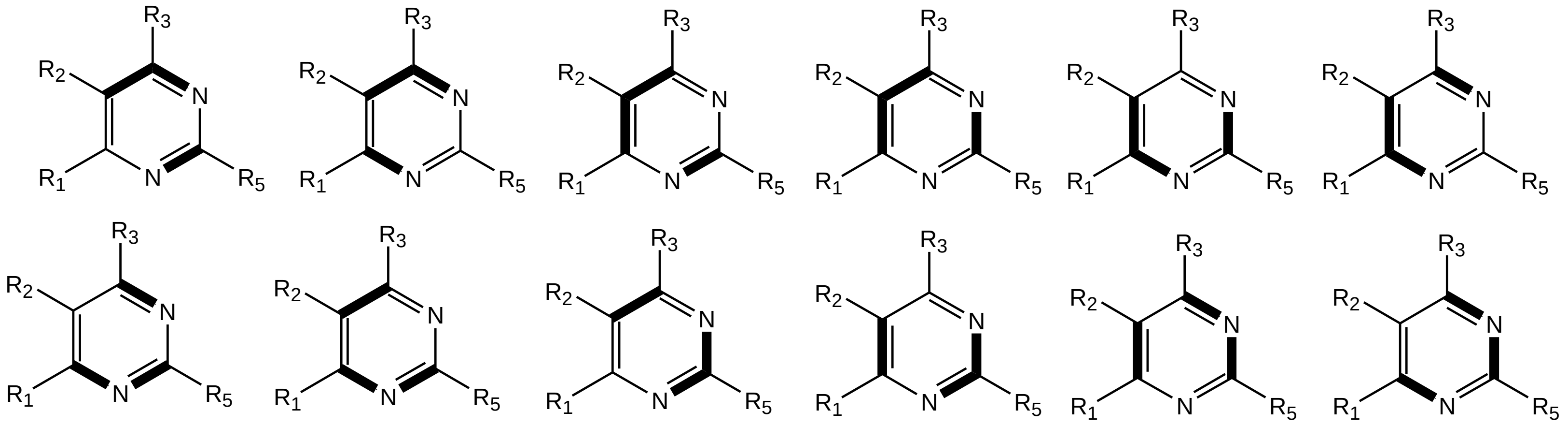

Schemes S26a. Superposition of [2 + 2 + 2] and [3 + 2 + 1] 3-partition templates for pyrimidine.

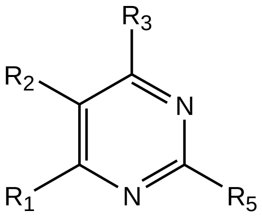

pyrimidine

[4+1+1]

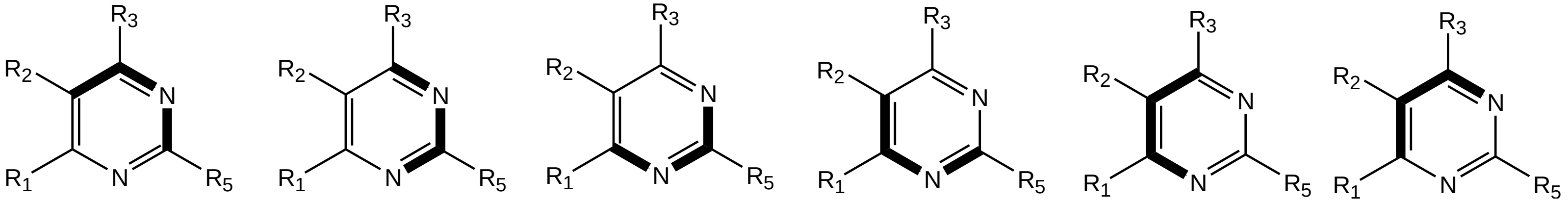

Schemes S26b. Superposition of [4 + 1 + 2] 3-partition templates for pyrimidine.

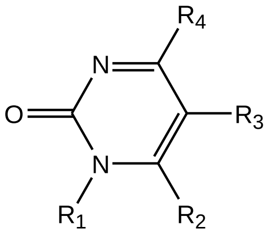

pyrimidone

[4 + 1 + 1]

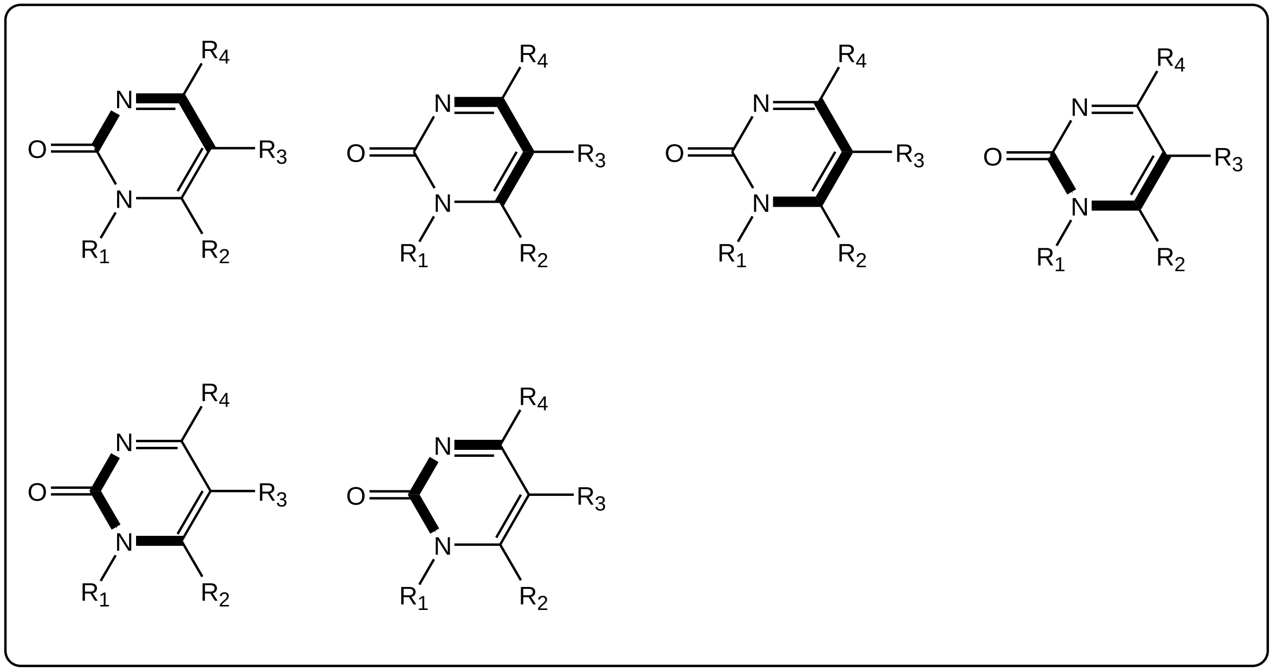

[2 + 2 + 2]

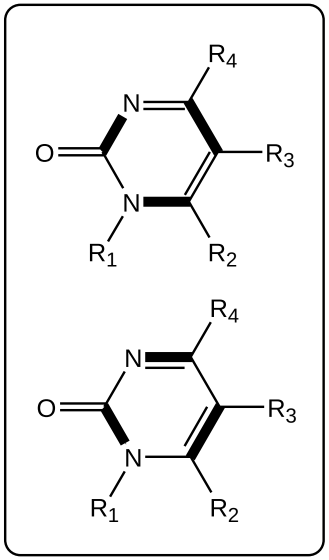

Schemes S27a. Superposition of [4 + 1 + 2] and [2 + 2 + 2] 3-partition templates for pyrimidone.

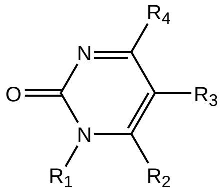

pyrimidone

[3 + 2 + 1]

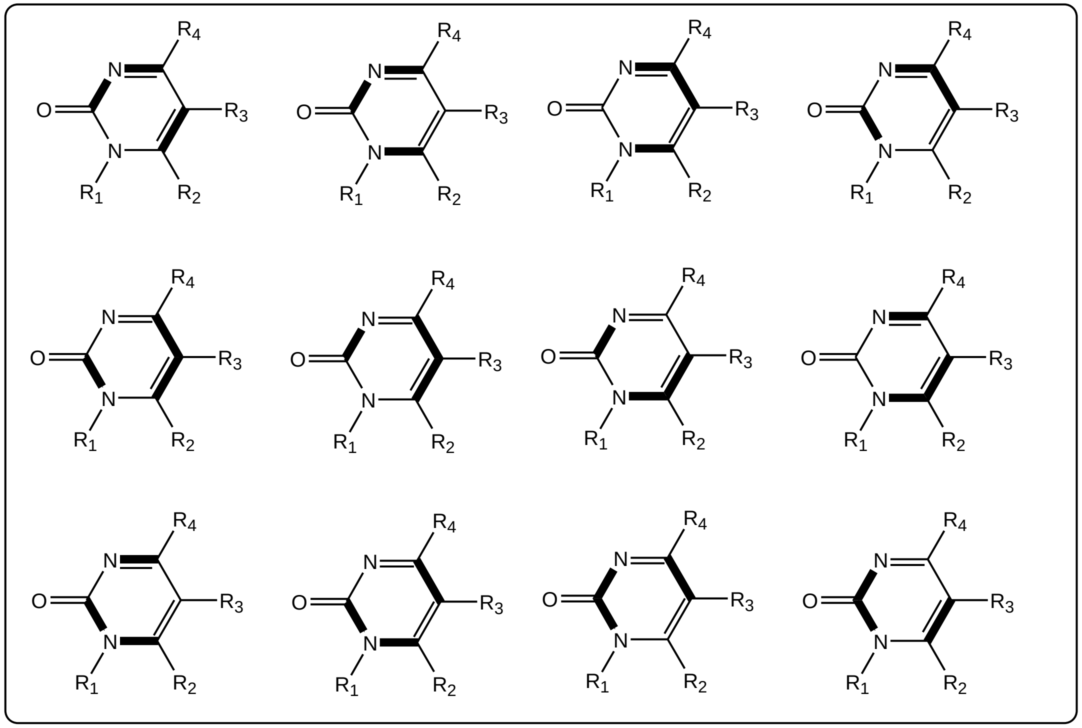

Schemes S27b. Superposition of [3 + 2 + 1] 3-partition templates for pyrimidone.

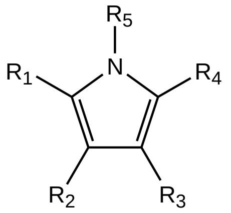

pyrrole

<u>three bond cuts</u>

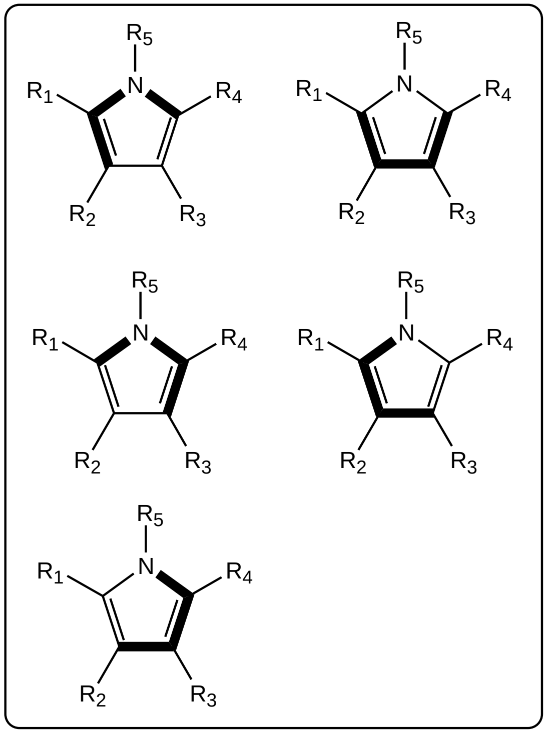

[3 + 1 + 1]

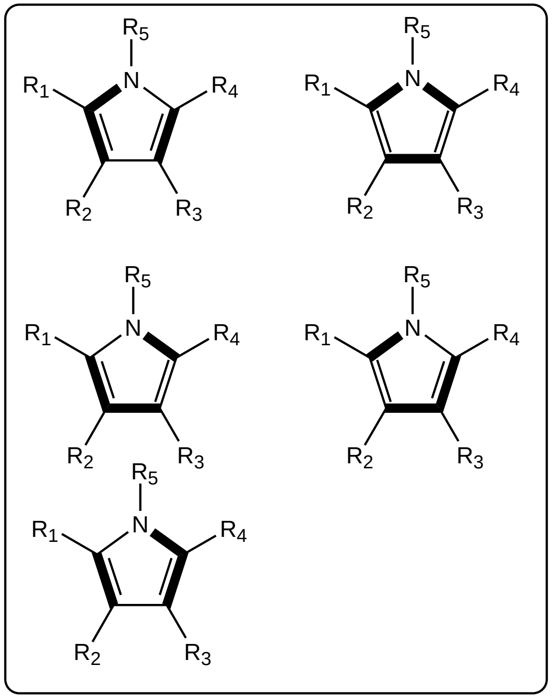

[2 + 2 + 1]

Schemes S28. Superposition of 3-partition templates for pyrrole.

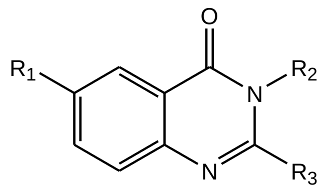

3*H*-Quinazolin-4-one

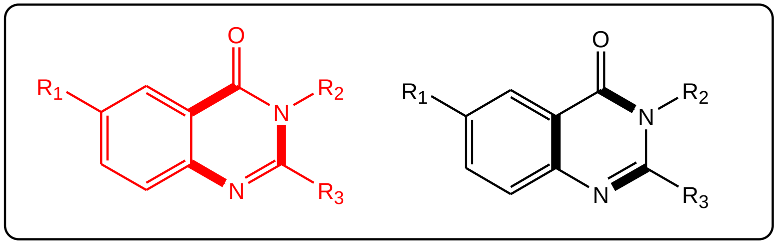

2 + 2 + 2

4 + 1 + 1

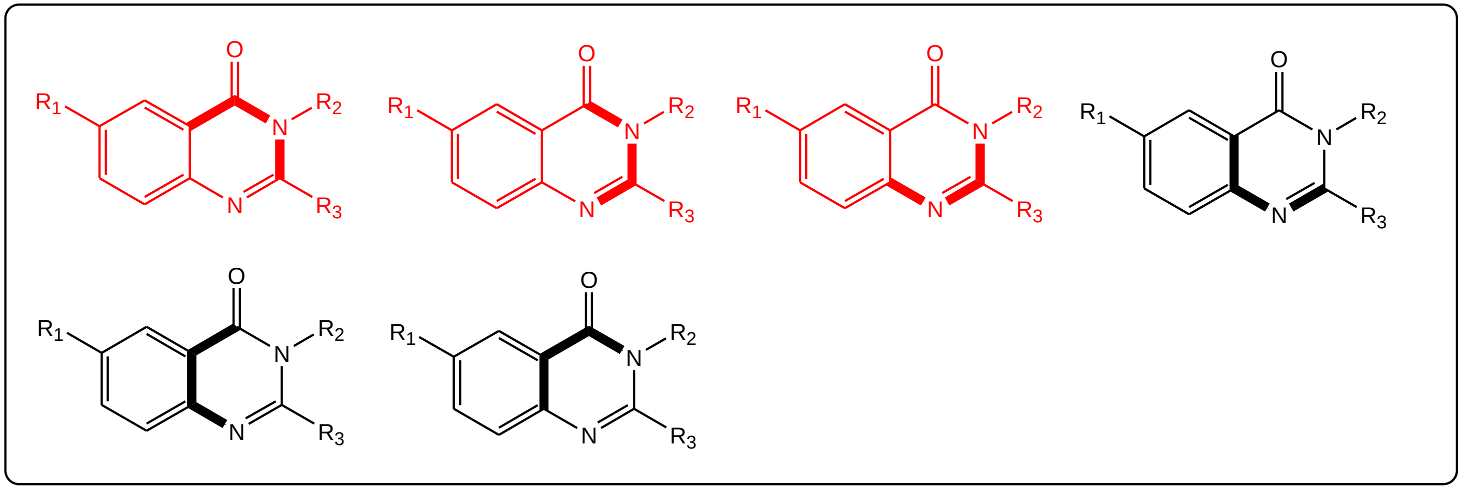

Schemes S29a. Superposition of [2 + 2 + 2] and [4 + 1 + 1] 3-partition templates for quinazolin-4-one.

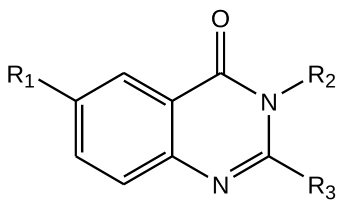

3*H*-Quinazolin-4-one

3 + 2 + 1

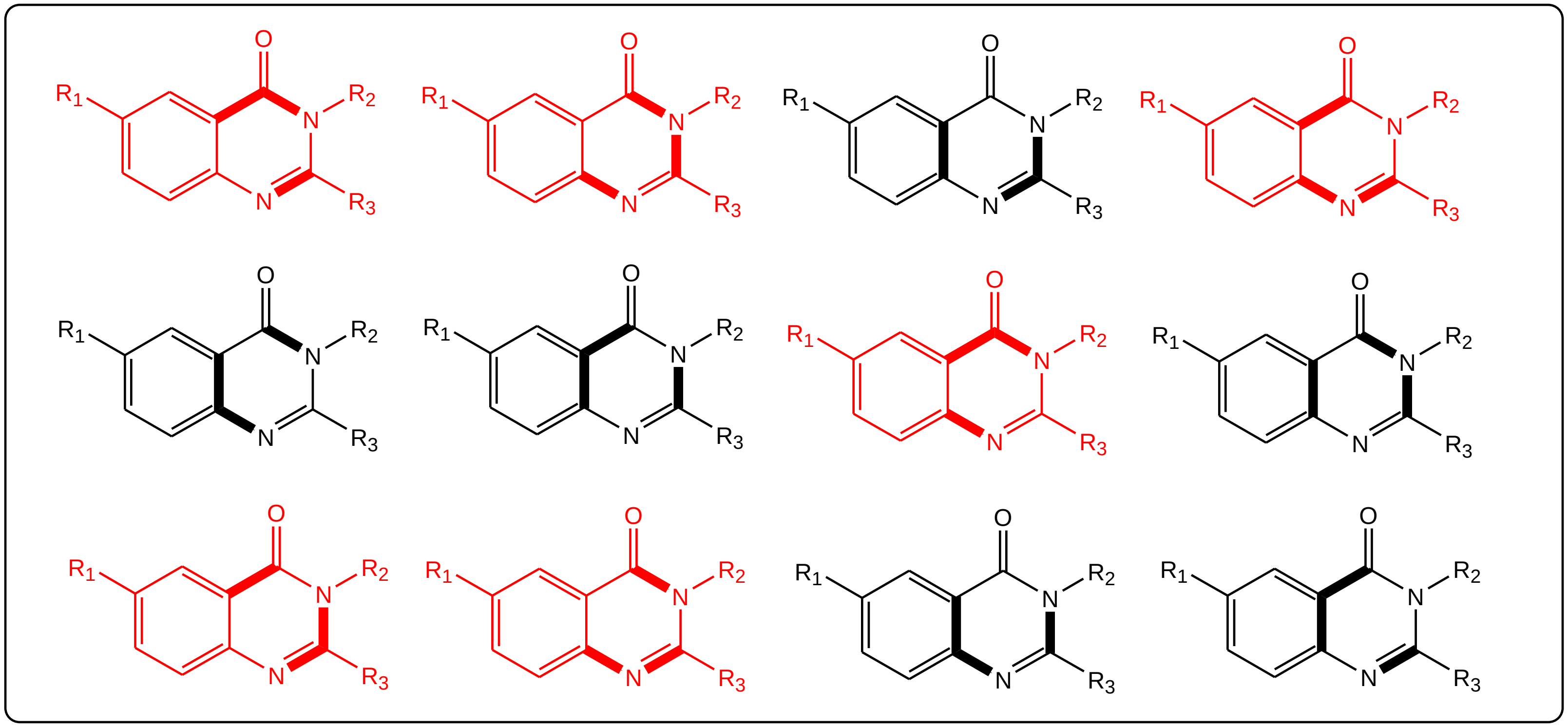

Schemes S29b. Superposition of [3 + 2 + 1] 3-partition templates for quinazolin-4-one.

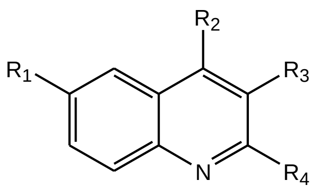

Quinoline

<u>three bond cuts</u>

3 + 2 + 1

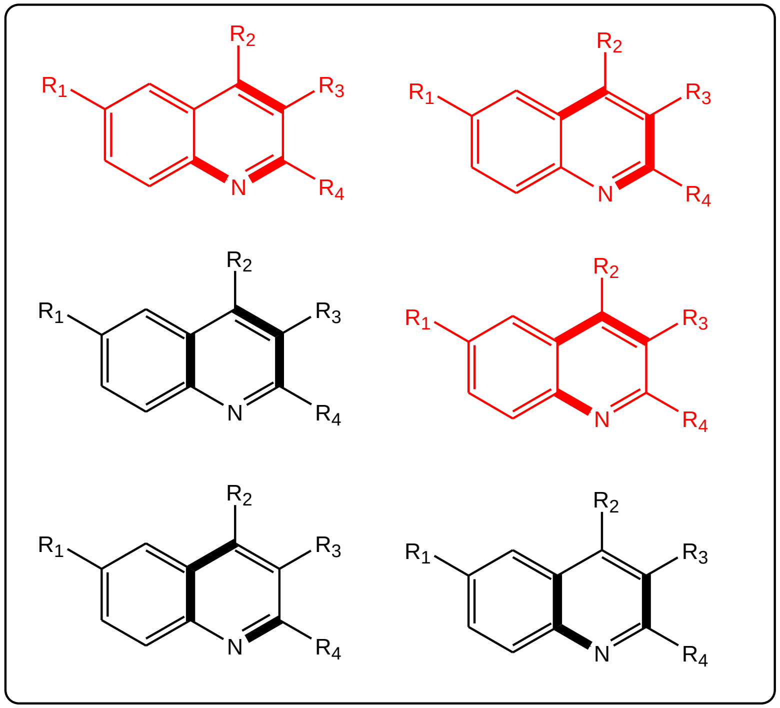

Schemes S30a. Superposition of [3 + 2 + 1] 3-partition templates for quinoline.

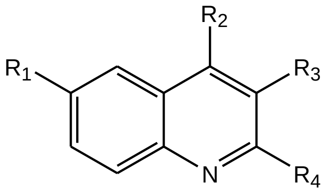

Quinoline

three bond cuts

4 + 1 + 1

2 + 2 + 2

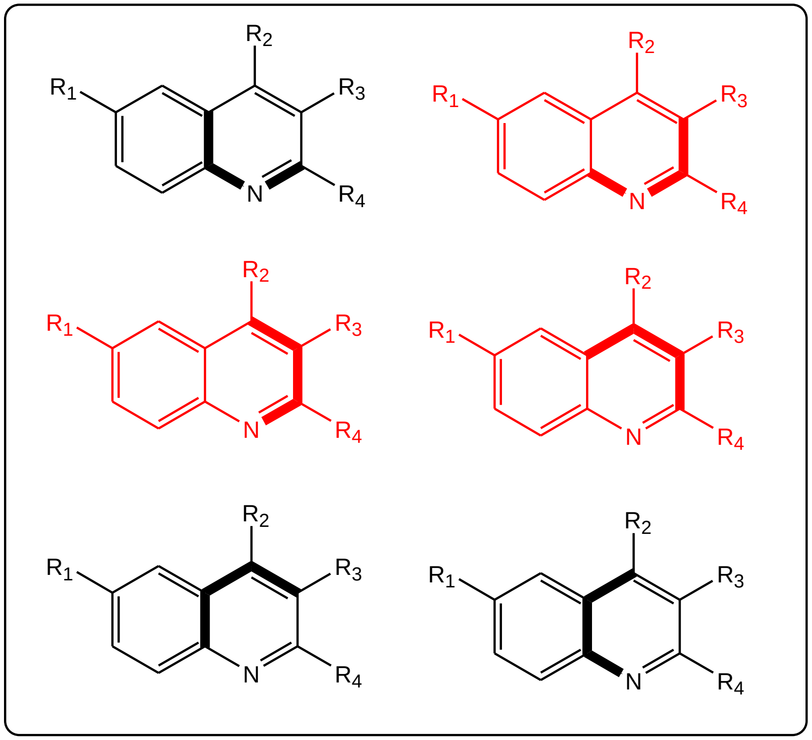

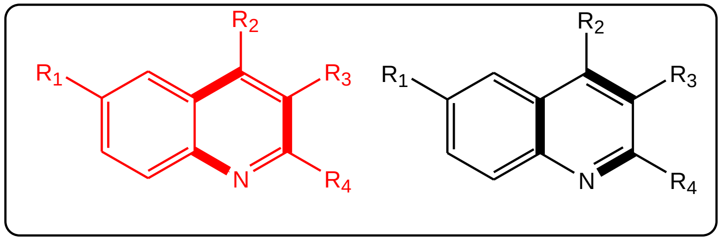

Schemes S30b. Superposition of [3 + 2 + 1] 3-partition templates for quinoline.

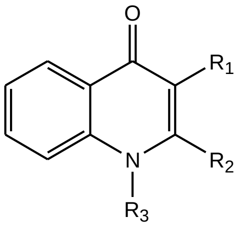

1*H*-Quinolin-4-one

4 + 1 + 1

<u>three bond cuts</u>

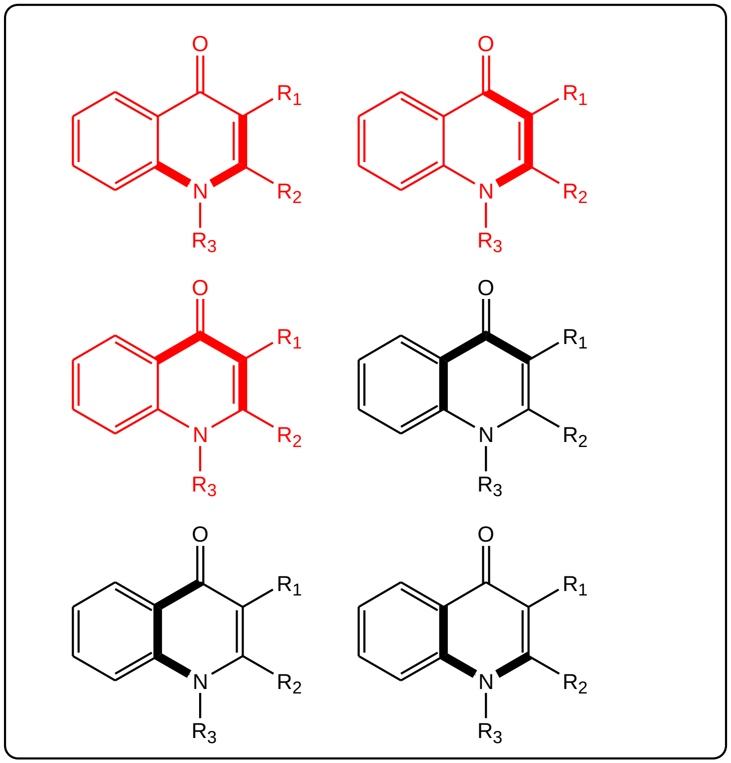

Schemes S31a. Superposition of [4 + 1 + 1] 3-partition templates for quinolinone.

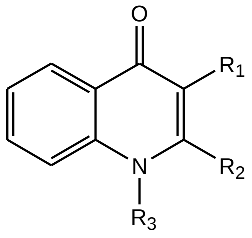

1*H*-Quinolin-4-one

three bond cuts

3 + 2 + 1

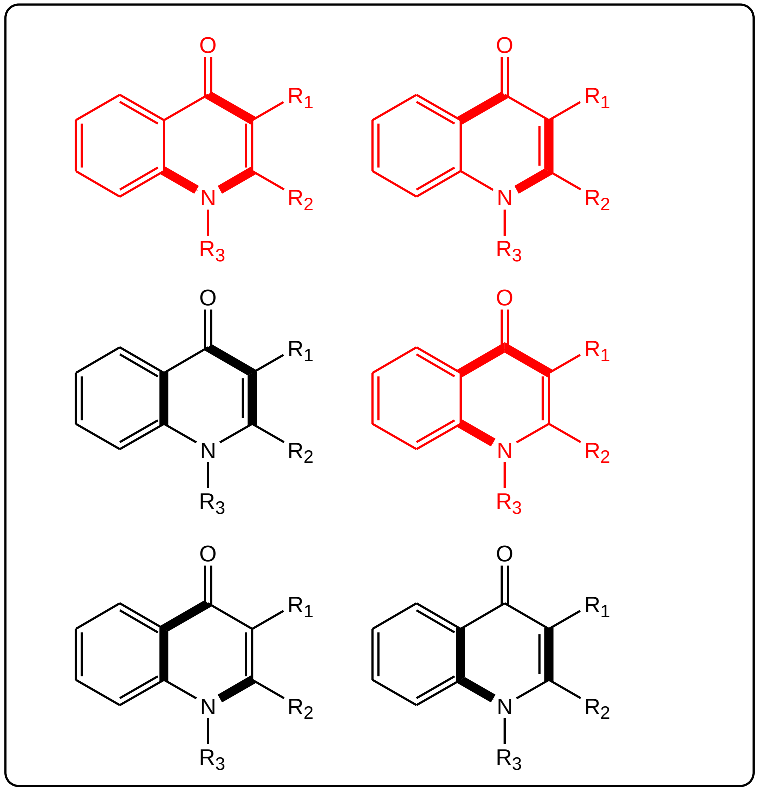

2 + 2 + 2

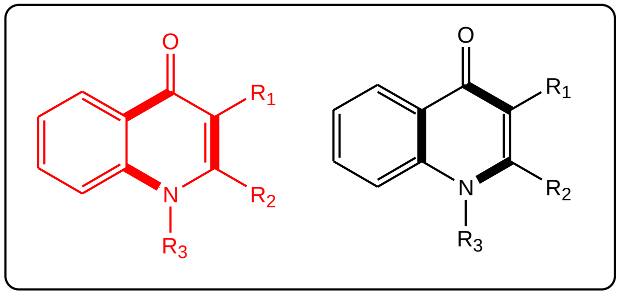

Schemes S31b. Superposition of [3 + 2 + 1] and [2 + 2 + 2] 3-partition templates for quinolinone.

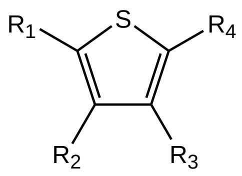

thiophene

three bond cuts

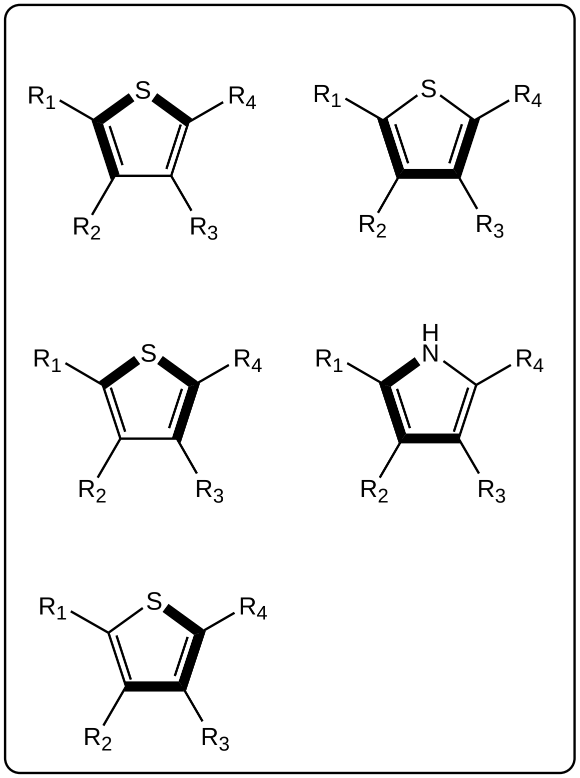

[3 + 1 + 1]

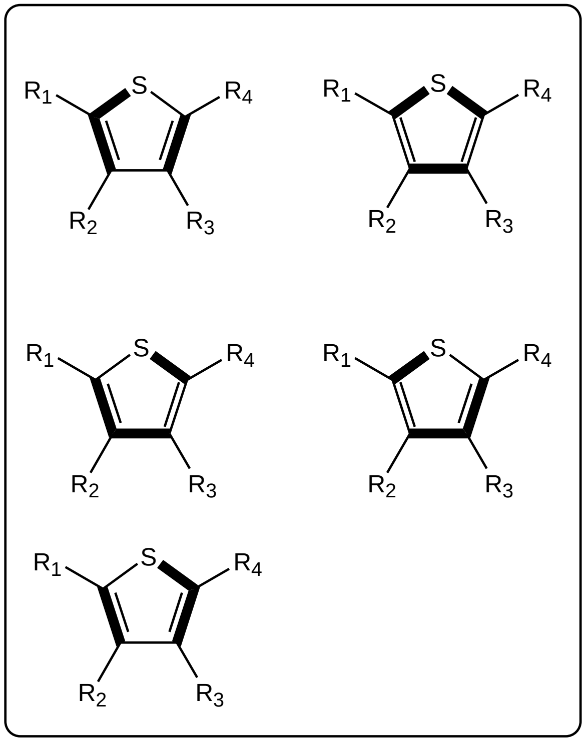

[2 + 2 + 1]

Schemes S32. Superposition of 3-partition templates for thiophene.
